# On the Nature of Guest Complexation in Water: Triggered Wetting–Water-Mediated Binding

**DOI:** 10.1021/acs.jpcb.2c00628

**Published:** 2022-04-19

**Authors:** Paolo Suating, Nicholas E. Ernst, Busayo D. Alagbe, Hannah A. Skinner, Joel T. Mague, Henry S. Ashbaugh, Bruce C. Gibb

**Affiliations:** † Department of Chemistry, Tulane University, New Orleans, Louisiana 70118, United States; ‡ Department of Chemical and Biomolecular Engineering, Tulane University, New Orleans, Louisiana 70118, United States

## Abstract

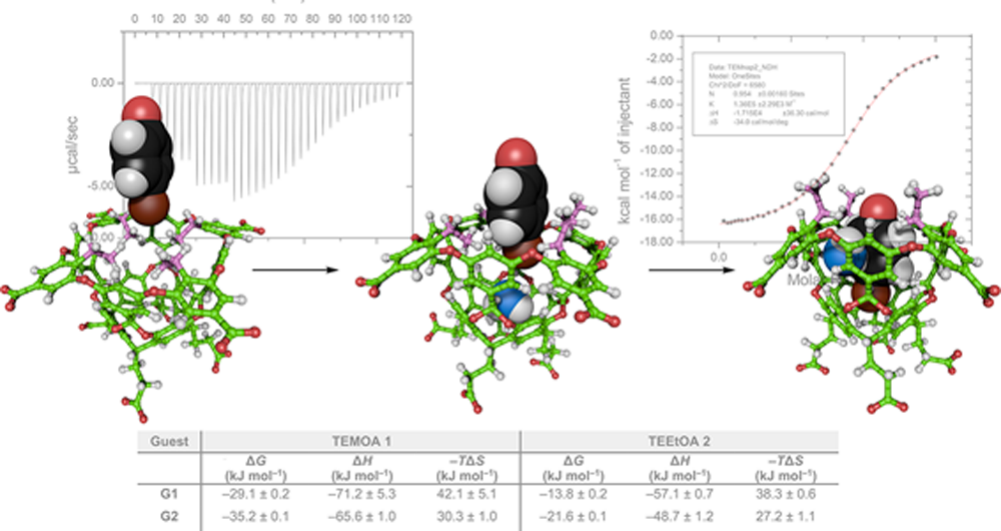

The complexity of macromolecular surfaces means that there are still many open questions regarding how specific areas are solvated and how this might affect the complexation of guests. Contributing to the identification and classification of the different possible mechanisms of complexation events in aqueous solution, and as part of the recent SAMPL8 exercise, we report here on the synthesis and conformational properties of TEEtOA **2**, a cavitand with conformationally flexible ethyl groups at its portal. Using a combination of ITC and NMR spectroscopy, we report the binding affinities of a series of carboxylates to **2** and compare it to a related cavitand TEMOA **1**. Additionally, we report MD simulations revealing how the wetting of the pocket of **2** is controlled by the conformation of its rim ethyl groups and, correspondingly, a novel triggered wetting, guest complexation mechanism, whereby the approaching guest opens up the pocket of the host, inducing its wetting and ultimately allows the formation of a hydrated host–guest complex (H·G·H_2_O). A general classification of complexation mechanisms is also suggested.

## Introduction

The complexity of the macromolecular surface means that there are still many open questions regarding how specific areas are hydrated and how this might affect the complexation of binding partners, ligands, or guests.^1,2^ For example, although the gross form of a protein is convex, below the ∼2 nm scale, the surface possesses many protuberances and concavities. Even if these surfaces were only composed of uncharged, hard (purely repulsive) surfaces, the small size and high cohesivity of water means that the solvation of the different types of surfaces would be varied and complex.^3−7^ Add some softness to the surface in the form of potential van der Waals interactions, add some hydrogen bond acceptors and donors, add some proximal charge groups, and it is exceedingly difficult to predict whether a nonpolar channel or concavity is dry or hydrated.^8−22^ Complicating things further, molecular dynamic (MD) simulations reveal that although the dynamic solvation of a surface might be temporally consistent, a whole new solvation regime opens up with the approach of a guest or ligand; the proximity of the guest induces water reorganization and water displacement.^23−28^ A simple bifurcation of the gamut of possibilities is that water can either attenuate or accentuate ligand affinity. Simple competition for the pocket by bound water(s) can account for guest affinity attenuation; however, we do not yet have a clear picture of the different mechanisms that can operate in cases of ligand affinity increase; such water-mediated binding events can be envisioned to occur in a myriad of different ways. It is little wonder, then, that the design of ligands for proteinaceous binding sites, and the estimation of their binding affinity, is so difficult.^1,2,7,29−33^

One way to approach the complexities of water solvation, and how it changes with ligand/guest complexation, is with structurally more straightforward model systems. Following this strategy, both wholly artificial constructs^26,27^ and host macrocycles^34−41^ have been investigated. With respect to the latter, one component of the Statistical Assessment of Modeling of Proteins and Ligands (SAMPL) is a series of blind predictive challenges focused on the thermodynamics of host–guest complexation.^29−31,42,43^ Each cycle begins with the release of a carefully designed set of hosts and guests. Subsequently, as research teams determine the different host–guest affinities using spectroscopic or calorimetric approaches, a community-wide exercise is opened for computational chemists to predict the strength of binding a priori. Finally, once all computational determinations have been submitted, the empirical data is released and the similarities/differences between the predicted and empirical data analyzed. The overarching goal of the SAMPL exercise is to push the boundaries of computational chemistry and advance computational techniques as predictive tools in drug design. At the same time, the use of small well-defined hosts and guests can provide exquisite details of host solvation and how this changes with guest complexation and, hence, the mechanisms by which water-mediated guest complexation can promote affinity.

In an ongoing project exploring the wetting of nonpolar surfaces, we recently demonstrated how, despite its relative openness, the pocket of tetra-*endo*-methyl octa acid **1** (TEMOA)^38^ is primarily dry. The absence of water within the cavity, water is essentially a poor guest for the pocket, means that guest binding is near maximal, that is, akin to the gas phase. A combination of MD simulations and densimetry revealed the dryness of the pocket, while guest affinity studies revealed the enhanced guest binding arising from this. As anticipated, wetting of the pocket occurs at elevated pressure, and in follow-on collaborative work with the Ashbaugh group, it was demonstrated how changes in functionality around the rim could also affect wettability.^35^ These results point to a two-state capillary evaporation model to describe the equilibrium between wet and dry states of concavities.

**Figure 1:**
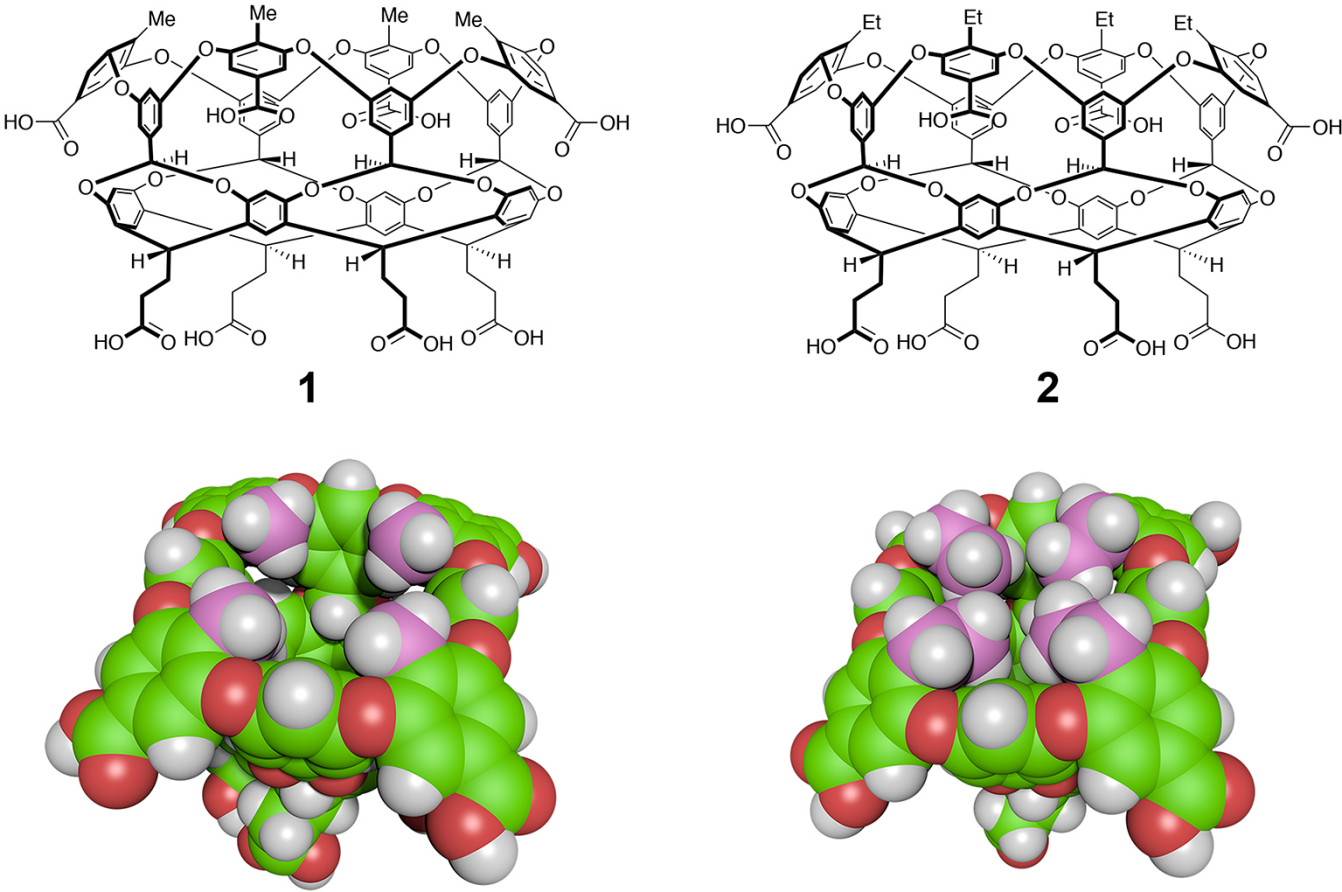
Structures and space-filling models of the two hosts used in this study: tetra-*endo*-methyl octa-acid (TEMOA, **1**), and tetra-endoethyl octa-acid (TEEtOA, **2**). Rim groups in both hosts are highlighted in pink. van der Waals structures were generated using ePMV for Cinema4D.^44^

To probe macrocyclic host hydration further, and as our part of the SAMPL8 exercise, we developed host **2**, tetra-*endo*-ethyl octa acid (TEEtOA, Figure 1). The overall shape of the host is the same as that of **1**, save for the extension of the rim methyl groups in **1** to ethyl groups in **2**. This change has multiple implications. First, it adds flexibility to the host; the ethyl groups can either point into or out of the pocket, which raises the possibility of an induced fit mechanism of guest binding. Relatedly, this flexibility leads to changes in the shape of the pocket. When the ethyl groups point into the cavity, the host will have a smaller pocket than that of **1** and might be expected to be primarily dry. Alternatively, when the ethyl groups are pointing out of the pocket the binding site is very similar to that of **1**. We describe here the synthesis of host **2**, assess its conformational preferences, compare its binding properties to that of host **1**, and use MD simulations to probe its solvation and the solvation changes during guest binding. These reveal a novel triggered wetting–guest complexation mechanism, whereby the approaching guest opens up the pocket of the host, induces its wetting, and ultimately allows the formation of a hydrated host–guest complex (H·G·H_2_O). Based on the observations here and elsewhere, we conclude with a general classification of complexation mechanisms in aqueous supramolecular chemistry.

## Results and Discussion

**Scheme 1:**
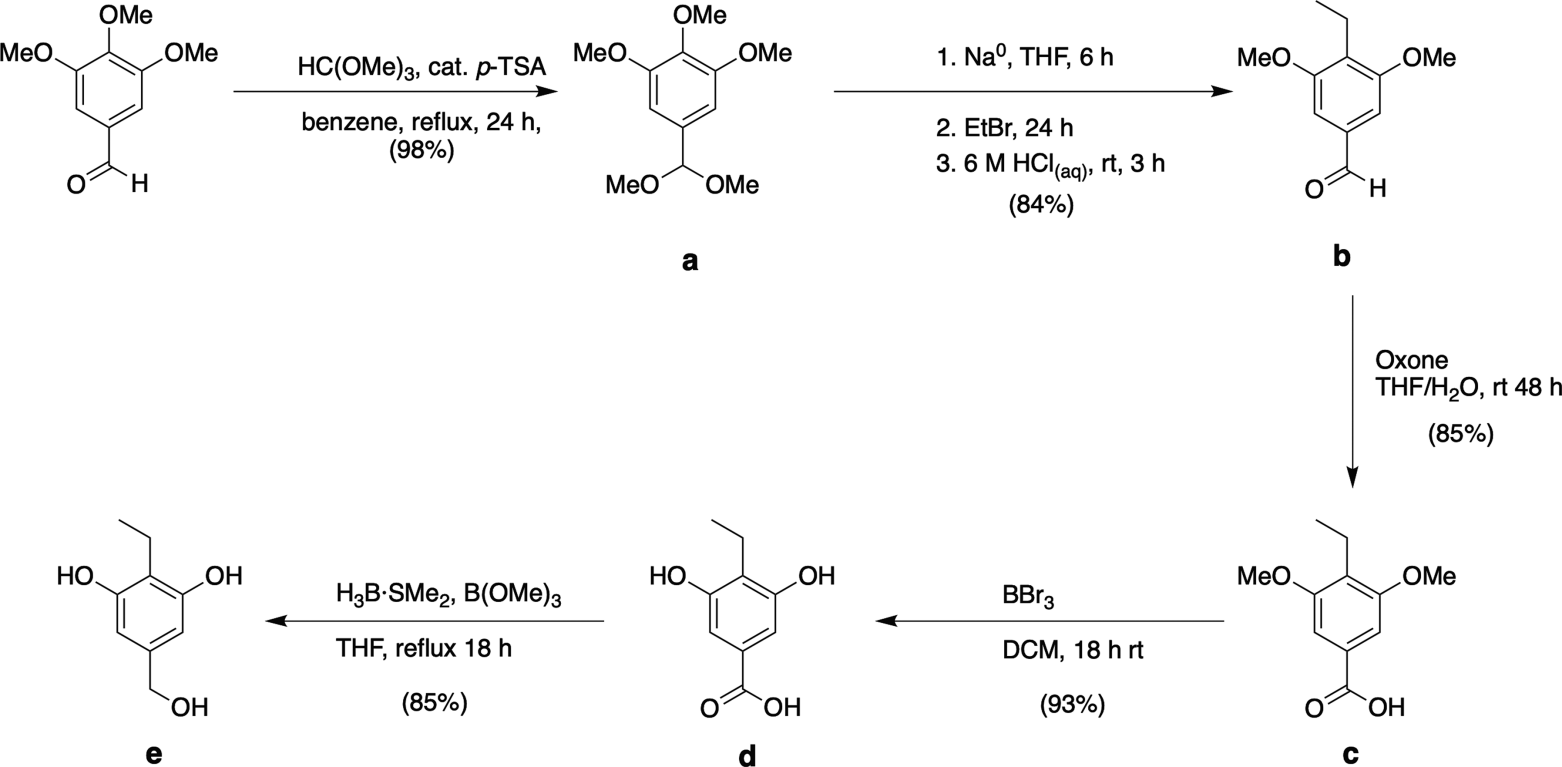
Synthesis of “Weaving” Material “**e**” from 3,4,5-Trimethoxybenzaldehyde

The formation of **2** requires the “weaving material” **e** (Scheme 1), the synthesis of which began with the conversion of 3,4,5-trimethoxybenzaldehyde to its dimethyl acetal **a** by reaction with trimethyl orthoformate in the presence of catalytic *p*-toluenesulfonic acid. Acetal **a** was then subjected to a reductive metalation and alkylation to selectively replace the 4-methoxy group with an ethyl substituent.^45^ After regeneration of the aldehyde moiety (**b**), oxidation with Oxone afforded carboxylic acid **c**, the structure of which was confirmed by X-ray crystallography.^46^ Subsequent demethylation with boron tribromide gave **d**, which was smoothly reduced to the benzyl alcohol **e** by reaction with borane-dimethyl sulfide complex.

**Scheme 2:**
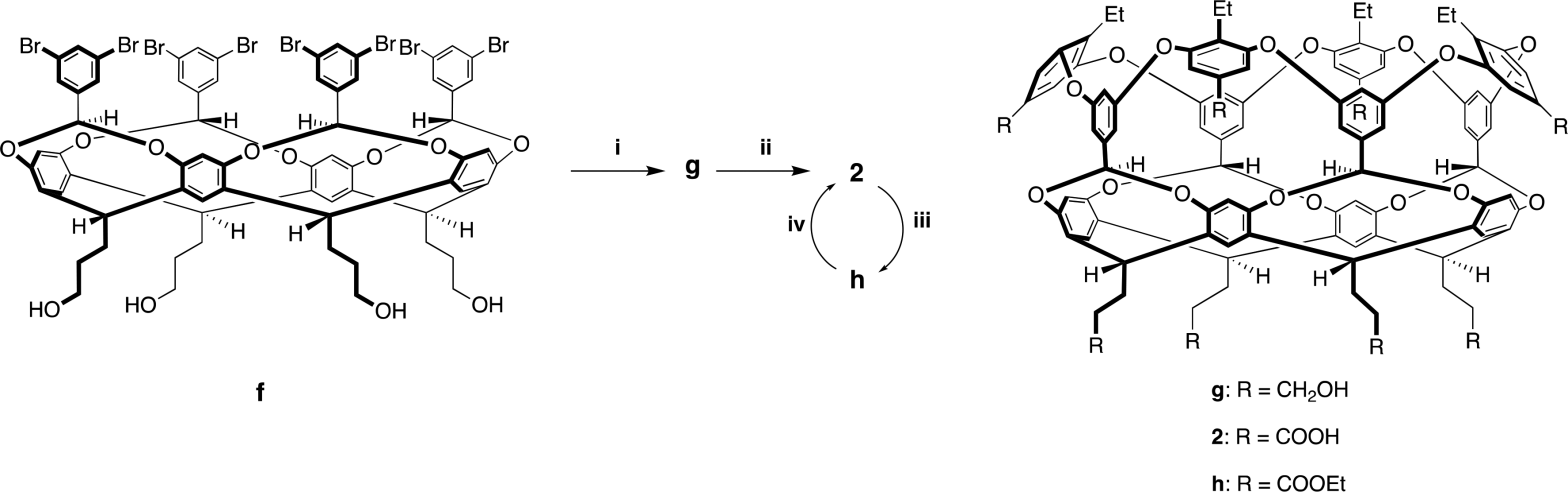
“Weaving” of Cavitand **f** with **e** (Scheme 1) and the Synthesis of Host **2** Conditions are **i**: K_2_CO_3_, CuBr·SMe_2_, pyridine, reflux 21 d (83% crude); **ii**: KMnO_4_, DMA/t-BuOH, 60 °C, 4 d (73% crude); **iii**: HCl, EtOH/CHCl_3_, reflux, 4 d (78%); **iv**: LiOH, DMA/H_2_O, 60 °C, 24 h (97%).

With benzyl alcohol **e** in hand, a weaving reaction, an 8-fold Ullman biaryl-ether coupling with octa-bromide **f**,^47−49^ gave octol cavitand **g** (Scheme 2). Without purification of this poorly soluble cavitand, KMnO_4_ was directly used to generate crude **2**. Finally, conversion to the octa-ethyl ester **h**, purification by column chromatography, and subsequent base-catalyzed hydrolysis, afforded pure cavitand **2**.

**Figure 2:**
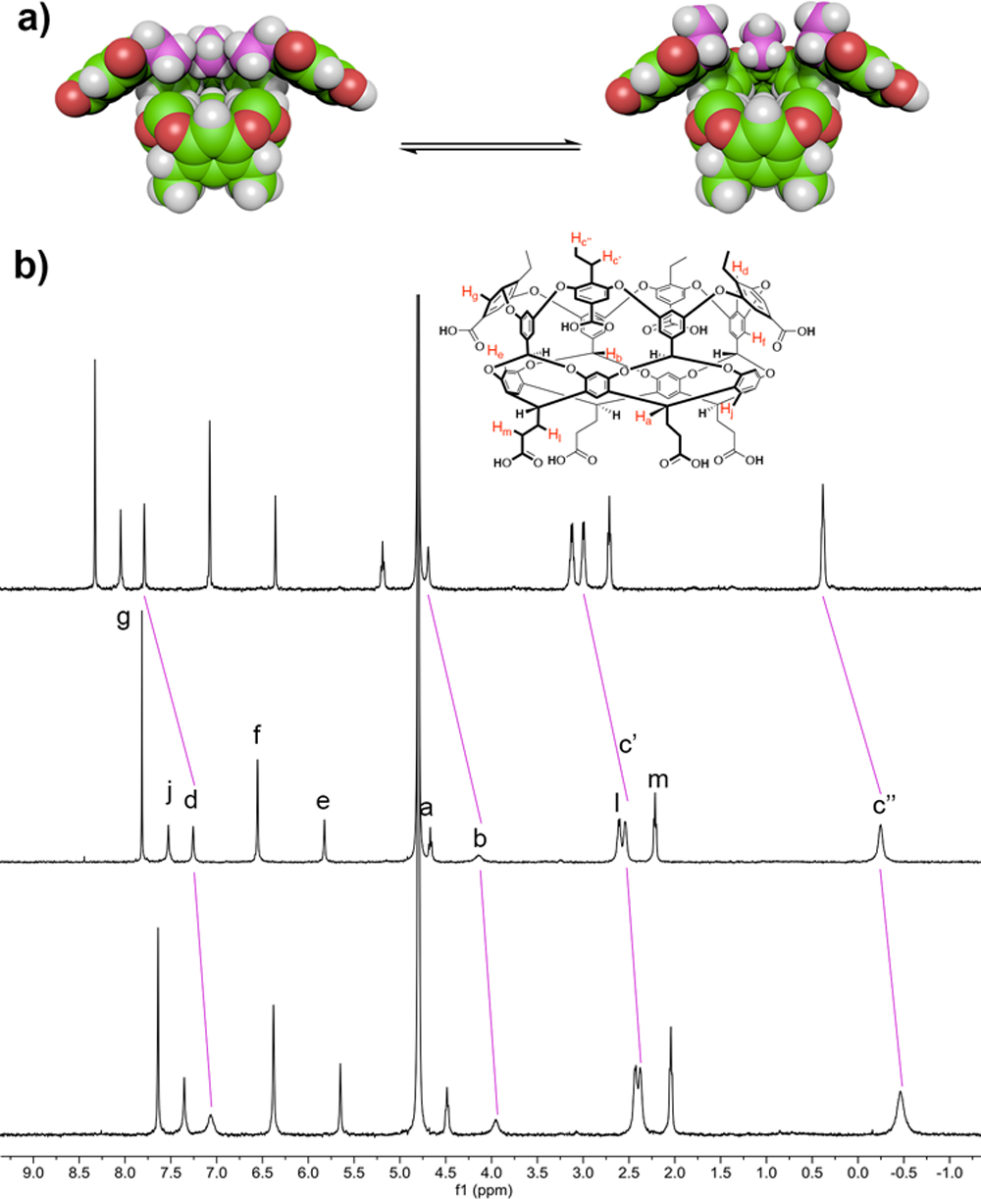
(a) Space-filling models of host **2** (with cut-away) showing the equilibrium between the in and out conformations of the rim ethyl groups. (b) Selected VT ^1^H NMR spectra of the host in 10 mM phosphate-buffered D_2_O, pD 11.45: 5 °C (lower), 23 °C (middle) and 55 °C (upper).

Models suggest that the rim ethyl groups of **2** are free to rotate, and at a rudimentary level one can envision a two-state model with all four ethyl groups either pointing into or out of the cavity (Figure 2a). We assume that when the host is in the free state the four groups are oriented inward to minimize exposure to bulk water. We designate this conformer as **2**, and the one with four ethyl groups pointing out as **2**–**4*****o***. The resting conformation of the host (**2**) has a minimally sized pocket, and hence, we envisioned that for guests larger than five nonhydrogen atoms to bind, some or all of the rim ethyl groups must adopt an “out” orientation. In the extreme (**2**–**4*****o***), models suggest a binding pocket of comparable volume to host **1** (albeit with a more prominent “collar” to the portal).

To attempt to quantify the barrier to ethyl group rotation, we utilized variable temperature ^1^H NMR spectroscopy (Figure 2b). Four broadened host signals are seen in the spectrum at 5 °C: those for H_d_ and H_b_ and those from rim ethyl groups H_c′_ and H_c″_. Located, respectively, at the rim and inside the pocket, protons H_d_ and H_b_ are proximal to the ethyl groups, and so evidently are themselves reporters for their in–out dynamics. This broadening of all four signals suggests a fast dynamic equilibrium between states and one that is perhaps not too far from the (500 MHz) NMR time scale.^50,51^ At 23 °C, H_d_ and H_b_ were observed to sharpen considerably, but there was still some broadening of the signals from H_c′_ and H_c″_. However, at 55 °C H_c′_ and H_c″_ began to resolve into a quartet and a triplet, respectively, demonstrating fast exchange at this temperature. It is noteworthy that as the temperature was raised, the H_c′_ and H_c″_ signals indicated a deshielding of their corresponding protons that was quite distinct from the average temperature dependent chemical shift (Δδ) of host protons distal to H_c′_ and H_c″_ (Table S2 and Figure S32 in the SI). Our interpretation of this is that although temperature does influence the chemical shift of all host signals, the changes in conformational preference of the ethyl groups brought about by increasing temperature has an additional influence. This is consistent with the notion that the frequency difference between the in and out positions is considerable and that **2**–**4*****o*** is the higher energy conformer that becomes more prevalent with increasing temperature.

As the barrier to ethyl group rotation was lower than that which could be probed by ^1^H NMR spectroscopy, we performed gas-phase computational analysis on a theoretical variant of **2** in which the pendent propanoic acid groups were truncated to methyls (SI). By carrying out stepwise (simultaneous) rotation of the four ethyl groups into the out position, we found a free energy difference between **2** and **2**–**4*****o*** of Δ*G* = 10.3 kJ mol^–1^, and a barrier between them of Δ*G*^‡^ = 46.5 kJ mol^–1^.^52^ Correspondingly, when a similar calculation was carried out in which only one ethyl was rotated out (**2** → **2**–**1*****o***), a free energy difference of Δ*G* = 2.17 kJ mol^–1^ and a barrier of Δ*G*^‡^ = 11.4 kJ mol^–1^ were calculated. This energy barrier is considerably higher than that of the rotation of the ethyl in ethylbenzene (calculated to be 4 kJ mol^–1^) but, nevertheless, corresponds to a *T*_coal_ below −200 °C. The broadness of the ^1^H NMR signals from H_c′_ and H_c″_ suggest that in water this barrier is likely considerably higher, but for practical purposes, both VT NMR spectroscopic analysis and gas phase calculations demonstrate the ethyl groups are essentially free to rotate and do so rapidly.

**Figure 3:**
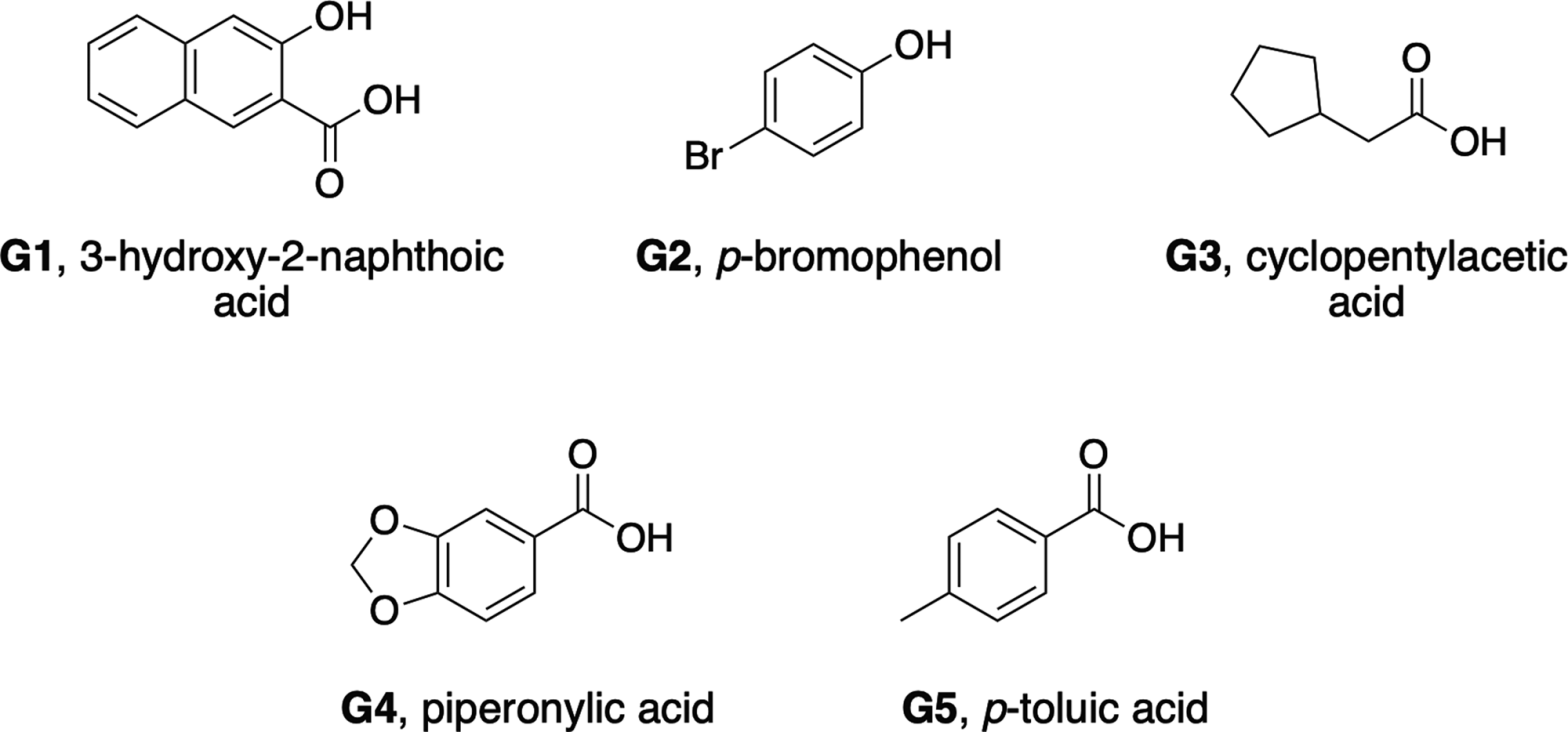
Guests used in this study. All guests were used as their sodium salts.

With a foundational understanding of host **2** in hand, we selected five guests to study their thermodynamics of binding to both hosts **1** and **2** (**G1**–**G5**, Figure 3). Models indicated that each guest **G1**–**G5** is too big to bind to **2** unless 2–4 ethyl groups turn out of the pocket. Thus, in each case an induced fit complexation process is required.

Complexation was determined for the polyanionic forms of hosts **1** and **2** at concentrations between 0.1 and 1.0 mM in pH 11.5 phosphate-buffered water. At this pH, both hosts were expected to be at least hexa-anionic^34^ and nominally octa-anionic,^48^ while the guests were fully in their conjugate base form (**G1** is monodeprotonated).

As evidenced by the movement of host signals and the broad and ill-defined bound guest signals, guest exchange was fast on the ^1^H NMR time scale; the ill-defined guest signals are consistent with the idea that they were undergoing the largest shifts between the free and bound state (*k*_coal_ = 2.22Δν). In contrast, the smaller shifts of the H_c′_ and H_c″_ signals meant that they were all still well-resolved. For example, in the case of **G2** binding to **2**, the shifts in H_c′_ and H_c″_ from the free to bound states were −0.48 and +0.98 ppm, respectively (Figure S37). Indeed, in all complexes examined with **2**, the H_c′_ and H_c″_ signals were deshielded and shielded, respectively, in the bound state. As the guests **G1**–**G5** are all too large for the resting state of the host (**2**), we attribute this consistency in signal shift to the rotation of some or all of the ethyl groups out of the cavity. However, the presence of the aromatic guest is also likely to influence these signal shifts. We return to the subtleties and details of the mechanism of guest binding below.

We utilized Isothermal Titration Calorimetry (ITC) to ascertain the free energy, enthalpy, and entropy of guest complexation (Table 1). Two host–guest pairs, **2**–**G3**, and **2**–**G5**, associated too weakly to determine affinity with ITC, and correspondingly, ^1^H NMR spectroscopy was investigated as an alternative to ascertain Δ*G* of binding. Although the affinity of **G5** to **2** could be ascertained by this technique, it transpired that the binding of **G3** to **2** was too weak to be accurately determined by either approach.

**Table 1:** Thermodynamic Data from ITC or ^1^H NMR Spectroscopy for the Binding^a^ of Guests **G1**–**G5** to Hosts **1** and **2**^b^

	TEMOA 1			TEEtOA 2		
guest	Δ*G* (kJ mol^–1^)	Δ*H* (kJ mol^–1^)	–*T*Δ*S* (kJ mol^–1^)	Δ*G* (kJ mol^–1^)	Δ*H* (kJ mol^–1^)	–*T*Δ*S* (kJ mol^–1^)
**G1**	–29.1 ± 0.2	–71.2 ± 5.3	42.1 ± 5.1	–13.8 ± 0.2	–57.1 ± 0.7	38.3 ± 0.6
**G2**	–35.2 ± 0.1	–65.6 ± 1.0	30.3 ± 1.0	–21.6 ± 0.1	–48.7 ± 1.2	27.2 ± 1.1
**G3**	–24.2 ± 0.1	–33.2 ± 1.0	09.0 ± 0.8	c	c	c
**G4**	–32.3 ± 0.1	–74.1 ± 1.4	41.8 ± 1.3	–18.7 ± 0.2	–54.3 ± 3.6	35.6 ± 3.4
**G5**	–27.9 ± 0.1	–59.6 ± 3.2	31.7 ± 3.1	–13.9 ± 0.1^d^		

a All experiments were performed in 10 mM phosphate buffer at pH 11.5 ± 0.05 at 25 °C for ITC measurements, or pH 11.9 ± 0.05 (pD 11.5 ± 0.05) in the case of NMR titrations (see SI, section E for details).

b The Δ*H* and *K*_a_ values were obtained by carrying out at least three separate experiments, averaging each set of data, and calculating the respective standard deviations. Δ*G* was obtained from *K*_a_ via the standard thermodynamic equation. The average Δ*H* and Δ*G* values were then used to calculate an average −*T*Δ*S*, and the corresponding standard deviations calculated using the standard equation for the propagation of uncertainties for subtraction. The deviations in Δ*G* were obtained by using the standard equation for the propagation of uncertainties for logarithms.

c Binding is too weak to be observed by NMR or ITC. Based on the difference in the average free energy of complexation to both hosts (⟨ΔΔ*G*⟩ = 12.9 kJ mol^–1^) and the value for **G3** binding to **1**, an affinity maximum for **G3** binding to **2** can be estimated to be approximately −10 kJ mol^–1^ or *K*_a_ ≲ 60 M^–1^.

d Determined by ^1^H NMR spectroscopy.

In both hosts, the order of increasing binding free energy, Δ*G*, was found to be **G3** < **G5** < **G1**, **G4** < **G2**, and all binding events driven by enthalpy (i.e., exothermic binding events with |Δ*H*| > |−*T*Δ*S*|). This nonclassical hydrophobic effect is typical for binding to a nonpolar concavity, and arises because poor solvation of the pocket leads to a lack of competition for the binding site by water, and hence a maximization of the host–guest noncovalent contacts.^38^ These contacts include the gamut of noncovalent interactions, primarily dipole–dipole, π–π stacking, C–H···π, dispersion, and in one case (see below) hydrogen bonding.

That guest **G2** is the strongest binder examined can be rationalized by the fact that the bromine atom can form four X···H–C hydrogen bonds with the H_b_ atoms in the interior of the cavitand.^47^ Indeed, this rather unusual interaction is also evidenced by the large enthalpic contribution (relative to **G5**) to the binding free energy of this guest. While we have not deconvoluted or decomposed these hydrogen bonds,^53^ we assume that like any weak hydrogen bond, their covalency is vanishing, their electrostatics moderate, and their polarization contributions (dispersion forces and other quantum mechanical interactions) relatively large.

Guest **G4** was the next strongest binder. It may be anticipated that **G4** would bind relatively weakly because the two ethereal oxygens would increase water solubility. However, we have previously shown that for a range of constitutional isomeric esters, the strongest binder to a dimeric cavitand assembly was the methyl ester.^54^ This was rationalized in terms of the electron-withdrawing methoxy oxygen inducing polarization of the methyl C–H bonds and leading to stronger interactions with the electron-rich aromatic walls of the host. We suspect a similar interaction here; not only is the five-membered acetal an ideal size for the very base of the pocket, but the methylene group represents the most electron-deficient moiety in the guest, which allows it to strongly anchor to the base of the host via C–H···π interactions.

The third strongest binding guest, naphthoic acid derivative **G1**, has the largest area of nonpolar surface to desolvate upon binding. Evidently, the benefit of this desolvation is more advantageous than the ability of the methyl group of the next to weakest binder, **G5**, to fill the very base of the pocket and act as a C–H···π anchor for the guest.^55^ Finally, we presume that the lack of any such anchor, combined with its small size, is the reason why **G3** is the weakest of binders.

A comparison of the ΔΔ*G* values for each guest binding to **1** and **2** reveals largely consistent differences of average ⟨ΔΔ*G*⟩ = 12.9 kJ mol^–1^ and standard deviation σ = 1.7 kJ mol^–1^. Because the pockets of **1** and **2**–**4*****o*** are so similar, we hypothesized that these ΔΔ*G* values are largely due to the flipping out of the ethyl groups in **2** to accommodate the guest (c.f. calculated Δ*G* for **2** → **2**-**4*****o*** = 10.3 kJ mol^–1^ (see above)). Other factors may, however, also come into play. For example, subtle differences in overall pocket shape may lead to intrinsically different direct host–guest interactions, as well as differences in pocket solvation which itself will lead to water-mediated affinity differences via water competition for the pocket (see below). To gain a better understanding of this state of affairs, and hence gain a better picture of the mechanism for guest binding, we carried out MD simulations. Specifically, we examined the hydration states of the pockets of **1** and **2**, the volume of the pocket of both hosts, the potential of mean force (PMF) of **G2** binding to **2**, as well as 10000 saved configurations from the PMF simulation.

**Figure 4:**
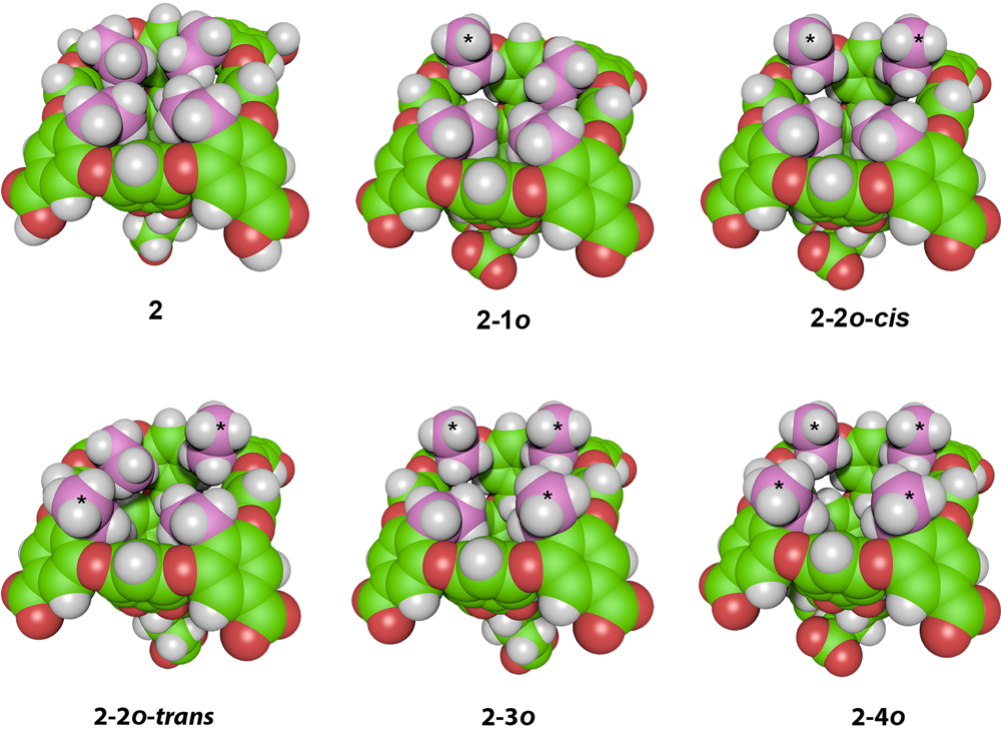
Conformations of the rim ethyl groups of host **2**. For clarity, the ethyl groups are highlighted in pink, with those oriented out of the pocket in each structure marked with a *. Top row, left to right: four ethyls in (**2**), one ethyl out (**2**-**1*o***), two adjacent ethyls out (**2-2*o*-*cis***). Bottom row, left to right: two opposing ethyls out (**2-2*o*-*trans***), three ethyls out (**2-3*o***), and four ethyls out (**2-4*o***). Structures were generated using ePMV for Cinema 4D.^44^

At a more detailed level, host **2** can exist in six distinct conformations (Figure 4). Specifically, all ethyl groups in **2** (the resting state of the host), one ethyl out (**2**-**1*****o***), two out (**2**-**2*****o***-***cis*** and **2**-**2*****o***-***trans***), three out (**2**-**3*****o***), and four out (**2**-**4*****o***). With the last of these possessing a pocket akin to **1**, we envisioned it would possess a relatively dry pocket^38^ but that, with each successive ethyl group pointing in, the pocket would be increasingly drier.

**Figure 5:**
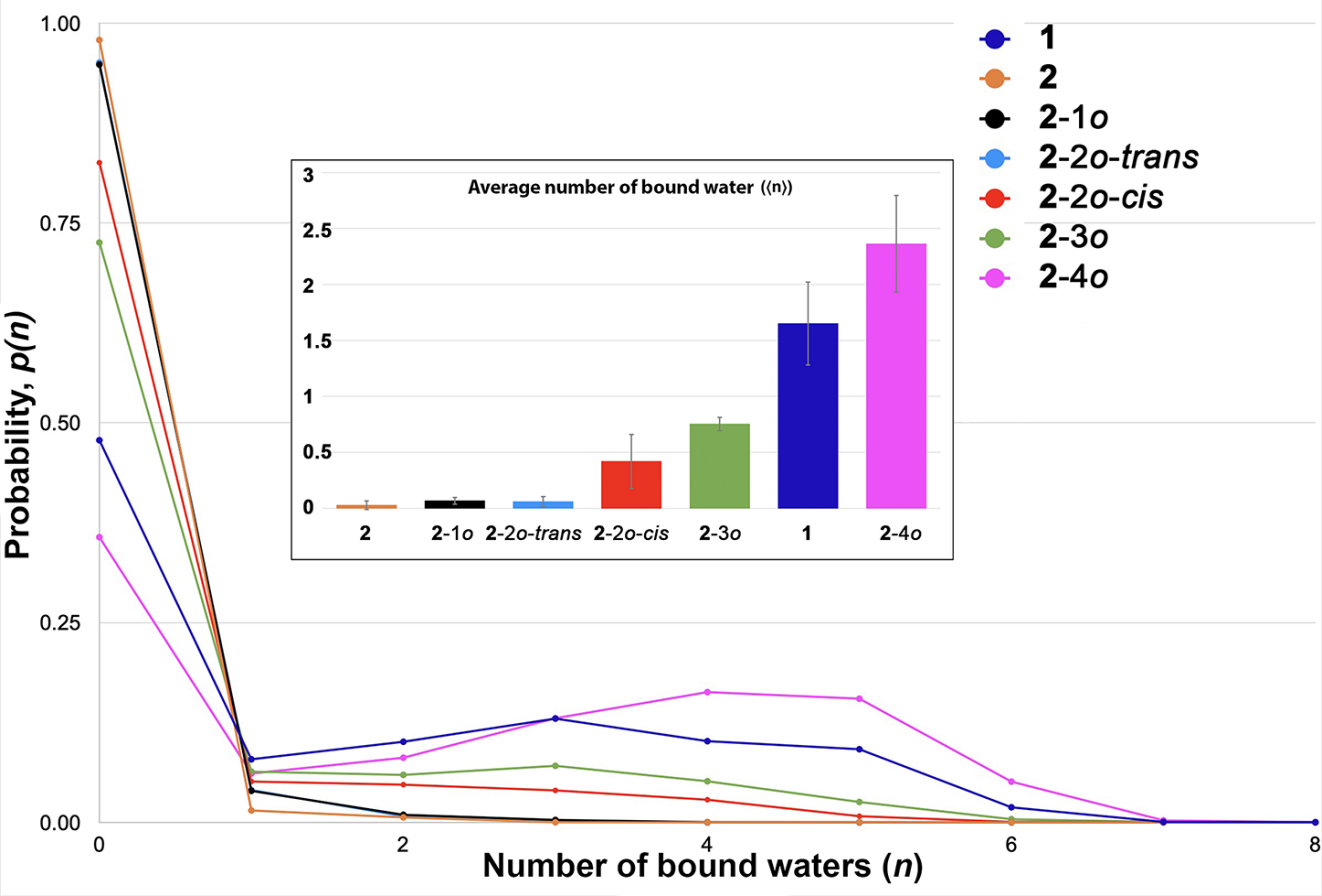
Probability distribution of the hydration (number of water molecules, *n*) of the pockets of **1**, **2**, **2**-**1*****o***, **2**-**2*****o***-***trans***, **2**-**2*****o***-***cis***, **2**-**3*****o***, and **2**-**4*****o***. Error bars (see SI) have been omitted for clarity. The probability distribution for **2**-**1*o*** and **2-2*o***-***trans*** are close enough that they are virtually overlaid. Inset: Bar graph of the average hydration number (⟨*n*⟩) of each host.

To evaluate the hydration of the pockets of these six conformations, we used the GROMACS 2016.3 simulation package (see SI for full details). In each simulation, the ethyl groups of each conformer were locked in either the *in* or *out* position, and the cavitand was placed in a bath of 2500 water molecules modeled using the TIP4P-Ew potential.^56^ The plane defined by the rim of ethereal oxygen atoms was used to define the boundary between the pocket and the bulk. Figure 5 graphs the probability of observing *n* waters inside the pocket of each host/conformer. Hosts **1** and **2**-**4*****o*** show a bimodal distribution with a ∼48% and ∼36% chance of finding zero water molecules in the pocket and a ∼13% and ∼17% chance of finding, respectively, three and four bound waters. In contrast, the probability distribution for host **2** in its resting conformation (ethyls in) is unimodal, with an ∼98% chance of finding the pocket completely evacuated; the pocket of **2** is barely, if ever, hydrated. Between **2** and **2**-**4*****o*** there is a continuum of increasing hydration, as each ethyl group adopts an out conformation so the probability of a totally dry pocket decreases from ∼96% to ∼48% and the probability of the pocket filling with three water molecules increases from 0% to ∼17%. Interestingly, the modeling predicts the **2**-**2*****o***-***trans*** conformation to have a drier pocket than the **2**-**2*****o***-***cis***.

The degree of hydration can also be gauged from the average number of bound waters (⟨*n*⟩ = ∑i*p*(i)) for each conformer (Figure 5, inset; SI, Table S10). By this useful metric, the degree of hydration of each conformer is (driest to wettest): **2** ∼ **2**-**1*****o*** ∼ **2**-**2*****o***-***trans***, **2**-**2*****o***-***cis***, **2**-**3*****o***, **1**, **2**-**4*****o***. Thus, within error, the pockets of the three conformers **2**, **2**-**1*****o***, and **2**-**2*****o***-***trans*** are all as dry as each other; they are, for all intents and purposes, unsolvated. In contrast, the hydration of the **2**-**2*****o***-***cis*** pocket is almost an order of magnitude greater that the **2**-**2*****o***-***trans*** conformer. The extent of hydration can be largely attributed to three classes of hydrogen bonding: between bound waters, between the bound and bulk waters, and between the bound waters and the host. Parenthetically, the last of these so-called “dangling” hydrogen bonds have been observed experimentally in aqueous benzene solutions using multivariate curve resolution Raman spectroscopy;^57^ they are enthalpically less favorable than a hydrogen bond to another water but are entropically less costly. In comparing the shape and the hydration of the pockets of the **2**-**2*****o***-***cis*** and **2**-**2*****o***-***trans*** conformers, we suspect that differences in hydrogen bonding between bound waters is key. We hypothesize that, because the ethyl groups of **2**-**2*****o***-***trans*** essentially divide the binding pocket into two to create two small pockets, each can contain no more than one water molecule. If a water molecule was to transiently occupy one of the pockets, it would find itself only able to form a hydrogen bond to the bulk and dangling hydrogen bonds. As a result, the pockets remain dry. In contrast, the *cis*-isomer is more capacious and has a small but relatively large chance of containing two to four waters. In such a scenario, stabilizing hydrogen bonding between bound waters would amount to an additional favorable contribution to the thermodynamics of hydration. In short, water is a better guest to **2-2*o***-***cis*** because more than one water can simultaneously bind.

The **2**-**3*****o*** conformer is almost twice as wet as the **2**-**2*****o***-***cis*** conformer (⟨*n*⟩ = 0.753 vs 0.419), but turning the fourth ethyl group outward leads to an even more significant increase in hydration: **2**-**4*****o*** is over three times wetter than **2**-**3*****o*** and 5–6× wetter than **2**-**2*****o***-***cis***. At first glance, the **2**-**4*****o*** conformer is wetter than host **1**, despite these having almost identically shaped pockets and calculated volumes (283.1 ± 1.2 versus 280.5 ± 1.4 Å^3^ for **2**-**4*****o*** and **1**, respectively). However, the error bars in both these measurements are large (Figure 5, inset), and better data would be needed to confirm any significant difference that may arise from dissimilarities in their respective networks of hydrogen bonding between bound and free (bulk) waters.

**Figure 6:**
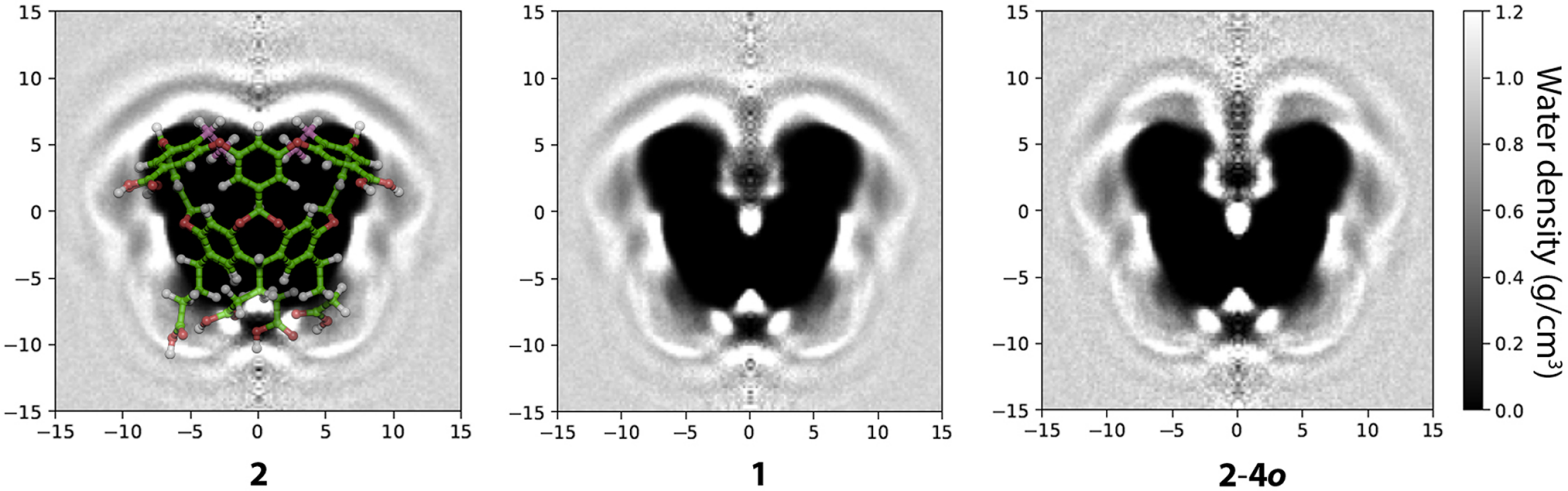
Water density maps about ***2***, **1**, and **2**-**4*****o***. The general orientation of the host in the three images is highlighted for host **2**. Each figure represents a cross-section of the cavitand (at 25 °C and 1 bar). The densities are cylindrically averaged around the *C*_4_ axes of each host and are reported in grayscale, with the very high electron density (cross-section) of each host masked out in black. The unsolvated pocket of **2** also appears in black.

The degree of pocket hydration is also illustrated by water density maps. Figure 6 shows the cylindrically averaged (around the *C*_4_ axis of the host) water density for **1**, **2**, and **2**-**4*****o***. Each image shows a cross section of the host, with the location of the oxygen atoms of water shown in greyscale. In each image, the high electron density of the cross-section of each host is masked black. As expected, the interior of **2** is completely dark, that is, devoid of water, while there is some water density residing within the interior of **1** localized around the ethereal oxygens at the rim and at the very base of the pocket. The same is true about the hydration sites of the pocket of **2**-**4*****o***, however, the water density is generally higher.

As mentioned previously, the ethyl groups of the host must swing out of the pocket for guests **G1**–**G5** to bind. As the pocket hydration data demonstrated an acute sensitivity to ethyl group conformation, we sought to probe the relationship between guest complexation, ethyl group conformation, and pocket hydration. To investigate this, we selected guest **G2** and calculated the potential of mean force (PMF) for the formation of its complex with host **2**. The PMF, which is essentially the free energy of binding as the guest is moved along a fixed coordinate, was calculated as the guest traveled along the *C*_4_ axis of the host and into the pocket. This value, calculated to be −30 kJ mol^–1^ (∼12*RT*) complements the ITC data for the host–guest pair (−21.6 kJ mol^–1^, Table 1).

**Figure 7:**
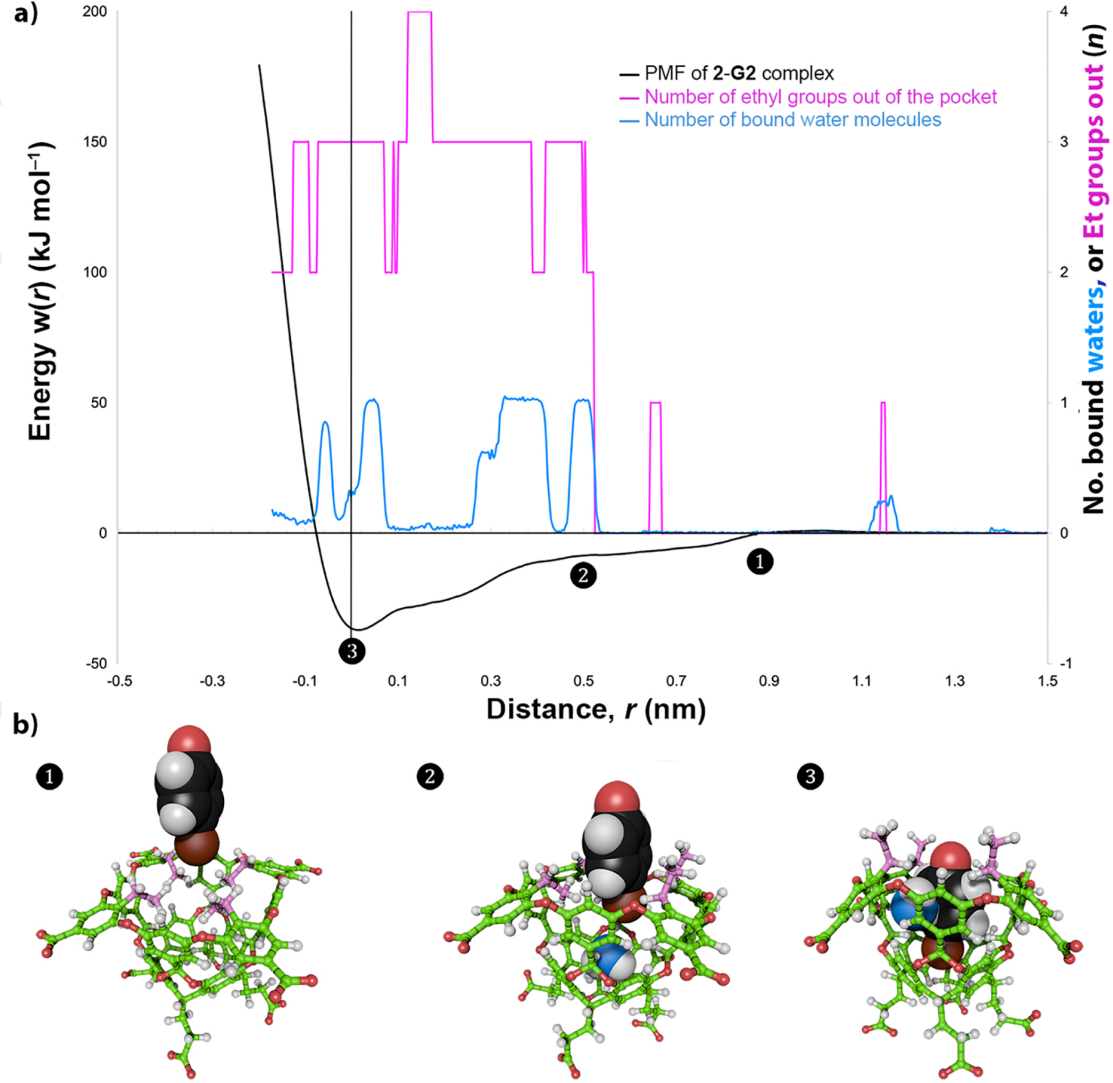
Detailed mechanism for the formation of the complex between **2** and **G2** calculated from simulations of the host, guest, and complex solvated by 2500 water molecules. (a) In this graph the *x*-axis shows the depth of the guest as it is inserted into the host. The potential of mean force (PMF, left *y*-axis) is shown as a black curve with a minimum at 0 nm. Superimposed on this plot is the number of ethyl groups in the host pointing out (pink line and right *y*-axis), along with the average number of bound waters (blue line and right *y*-axis). The data are taken from 10000 saved configurations from the simulation. The indicated points on the PMF curve ((1), (2), and (3)) correspond to the structures shown below in (b). Structures (1), (2), and (3) show the positions of **G2** relative to **2** at the indicated points on the PMF profile. In the three structures, the flexible ethyl groups are highlighted in pink, and any bound water molecules are shown in blue. The structures were generated using ePMV for Cinema 4D.^44^

Figure 7a shows the PMF profile for this complexation (black line), with *r* = 0 defined as a dummy atom where the *C*_4_ axis intercepts the plane defined by the centers of the four benzylic carbons to which the H_a_ protons (Figure 2) are attached. In general, the PMF landscape is uneven compared to that of guest binding to a similar host devoid of ethyl groups,^37^ illustrating the complexity that the ethyl substituents bring to the binding event. Building on this, we also carried out a postsimulation analysis of 10000 saved configurations from the PMF calculation to provide a picture of the changes in the ethyl group conformation and pocket hydration during guest complexation (Figure 7a,b). As the guest approaches the portal of the pocket (*r* ≳ 0.9 nm, (1)), the host is in its resting state with the four ethyl groups oriented into the pocket (pink line in Figure 7a; ethyls highlighted in pink in Figure 7b). During this segment of the simulation, only very occasionally were the ethyl groups observed to flip outward to give the **2**-**3*****o*** state (Figure 7a). As expected, the pocket is essentially dry (blue line in Figure 7a) in this conformation.

As the guest begins to enter the pocket (*r* ≈ 0.5 nm, (2)), three ethyl groups move out of the cavity. There are occasional fluctuations to the **2**-**2*****o*** and the **2**-**4*****o*** states, but to a first approximation, the switch from the **2** conformation to the **2**-**3*****o*** conformation is complete. Presumably, the fourth ethyl group does not have to swing out because of the slim nature of the guest. As the guest is entering the cavity, a water molecule (Figure 7a) slips in to occupy the void at the very base of the pocket (Figure 7b, c.f. water density in Figure 6). The switching from a dry cavity to one with a bound water suggests that wetting represents a thermodynamic minimum, but higher levels of sampling are required to accurately determine this. Regardless, most snapshots have this guest water oriented as shown in Figure 7b, acting as a double hydrogen bond donor to two opposing aromatic rings in the wall of the pocket. Here, the guest water has little option but to only hydrogen bond with the host; at least transiently until the slower binding **G2** “catches up”.

As guest **G2** binds completely into the pocket (*r* ≈ 0, (3)), the bound water is pushed out of the bottom of the pocket; the hydrogen bonding between it and the host is no thermodynamic match for the formation of four X···H–C hydrogen bonds between **G2** and the H_b_ atoms of the cavitand.^47^ However, this guest water does not entirely vacate the pocket. As Figure 7a and b show, frequently one water molecule can be found in the pocket bound with **G2**, sandwiched between the aromatic face of the guest and the aromatic wall of the pocket. Labeling the bound water in structure (2) reveals that ∼90% of the time it translocates to the upper section of the pocket as **G2** docks. In other words, in most saved configurations, the bound water in structures (2) and (3) are one and the same. Presumably, the bound water in (3) is stabilized by both hydrogen bonding to the bulk and its weaker dangling hydrogen bonding to the wall of the host and the aromatic ring of **G2**. However, an estimation of its precise thermodynamic stability would require much longer simulations to obtain accurate exchange kinetics with water in the bulk. What is clear, however, is that in the case of **G2** (and presumably the other aromatic guests), the pocket of the host is wetter when it binds a guest than when it is empty; the bound water is integral to the stability of the host–guest complex. The final stages of **G2** binding result in little change in the conformations of the ethyl groups (Figure 7b); barring the occasional flipping of one ethyl group into or out of the pocket, the dominant form is the **2**-**3*****o*** conformation. And so, in the bound state, the pocket of host **2** is occupied by **G2**, a water, and one of its ethyl groups.

**Scheme 3:**
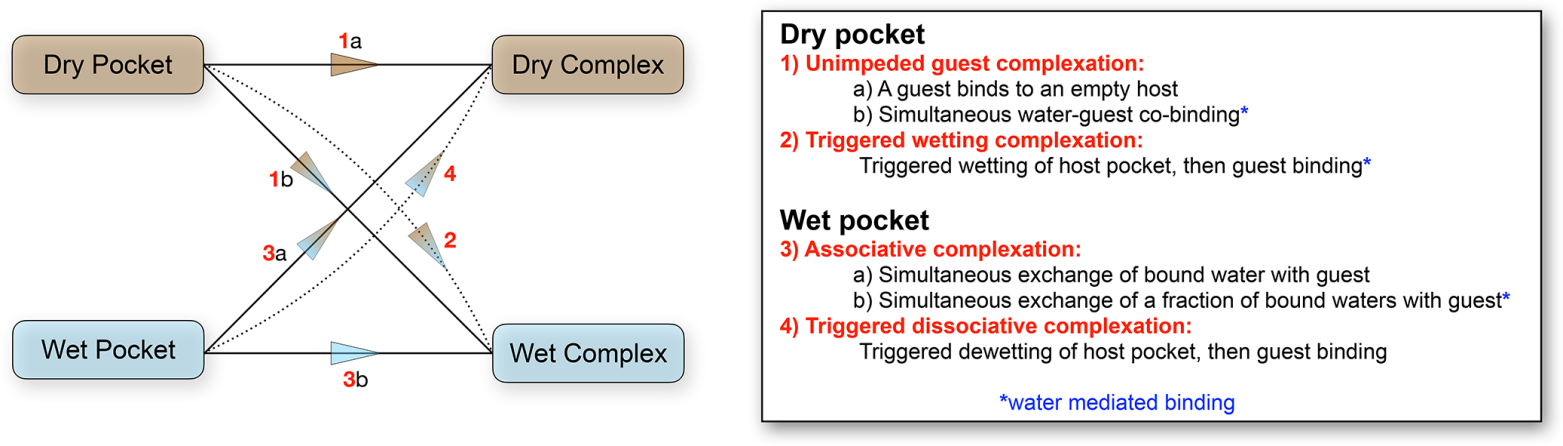
Mechanisms for Host–Guest Complexation in Aqueous Solution For simplicity, we treat dry and wet hosts separately. The inclusion of stochastic wetting of a dry host and drying of a wet host leads to duplication of mechanisms and difficulty in classification.

Where does the binding of **G2** to host **2** lie within the different possible complexation mechanisms in water? To our knowledge, there is as yet no classification system for binding events in aqueous solution, but the unusual small size and high cohesivity of water suggests a scheme more complex than that in organic media, which can be bifurcated into associative and dissociative processes. This idea is further supported by an inspection of binding processes reported in the literature, which suggests a useful approach is to consider the hydration state of the pocket of the free host and the hydration state of the bound guest. In the following analysis (Scheme 3) we assume that for the free host there is no equilibrium between the dry and wet states; either the host is dry or it is wet. Such an idealized system is needed for the sake of simplicity; to invoke stochastic wetting of a dry host or drying of a wet host leads to the duplication of mechanistic possibilities and difficulties in formal classification.

### Unimpeded Guest Complexation

This is the simplest of mechanisms: the pocket is devoid of water and is free to bind a guest; no water binds with the guest, that is, a simple 1:1 host–guest complex is formed (Scheme 3, 1a). This is likely a relatively rare situation, since the pocket must be open enough for a guest to bind but not allow any adventitious water molecules to enter in the absence of a guest. Indeed, a binding pocket that is never solvated by water, but can bind a guest molecule, probably represents a theoretical concept more than any real situation (methane binding?). However, very dry to almost dry pockets have been observed,^38^ and it is likely that the very strong complexation of guests to some cucurbiturils arise because their pockets are close to dry, and therefore, binding is akin to the gas phase and is thus maximal.^39,40^

A variation of this process is the binding of a water–guest complex to form a wet complex Scheme 3, 1b). We are unaware of any definitive examples whereby a guest and one or more closely associated water molecules bind simultaneously to a dry host. Indeed, given the small size of water and its rapid dynamics of movement, it may be accurate to state that in the majority of cases, water would precede guest entry into the pocket of a host. This point notwithstanding, it is not difficult to conceive of a strong guest–water complex that itself is bound to a dry host.

### Triggered Wetting Complexation

In the idealized triggered wetting complexation, the approach of a guest triggers a conformational change in the host, which allows the pocket of the host to switch from a dry to a wet state (Scheme 3, 2). This is the mechanism reported here (Figure 7). It may not necessarily be the guest that triggers a change in the host, but some form of external stimulus is needed for water to become an acceptable guest and ultimately allows the formation of a hydrated host–guest complex. In this situation, water mediates guest complexation and enhances the overall thermodynamic affinity; the one or more bound water molecules are thermodynamically integral to the stability of the host–guest complex. Parenthetically, it is worth noting that the formation of a hydrated complex can also arise when water is kinetically trapped at the base of a cavity by a large entering guest.^28^ However, such cases do not represent thermodynamic minima and are not examples of water mediation.

### Associative Complexation (Simultaneous Water–Guest Exchange)

This first example of the binding properties of a hydrated pocket is the classic associative binding mechanism, whereby a guest pushes water out of a pocket (Scheme 3, 3a). The most favorable situation whereby this can occur is with twin-portal, “tubular” hosts such as cyclodextrins,^58−60^ cucurbiturils,^61,62^ and pillarenes.^63^ A variation on this theme is where a relatively small, well-hydrated guest displaces only a fraction of the waters in the pocket of a host (Scheme 3, 3b). We are not aware of any detailed studies of such a mechanism, but such a process seems to be the most likely mechanism in the binding of (solvated) polarizable anions to cyclodextrins, cavitands, and other such hosts.^64−67^

### Triggered Dissociative Binding (Stepwise Water–Guest Exchange)

Simplifying the classification of guest complexation mechanisms by neglecting stochastic wetting/dewetting of a host precludes a classic dissociative guest binding mechanism. Consequently, one need only consider a triggered dissociative mechanism, whereby external stimuli, most commonly the approach of a guest molecule, triggers the dewetting of the pocket to allow the guest to bind. Dewetting of the pocket is triggered by the approaching nonpolar guest destabilizing the bound water by disrupting its hydrogen bond network with the bulk. This mechanism has been observed by Rick while studying the solvation of the octa-acid cavitand using MD simulations^34^ and by Setny in MD simulations of wholly artificial concavities.^26^

These four general classes of host–guest complexation in water represent six distinct mechanisms for binding events in water. Half of these are water-mediated, that is, water is intimately involved in the complexation process and is a contributor to the thermodynamic stability of the resulting hydrated host–guest complex (H·G·*n*H_2_O). This greater variety of binding mechanisms relative to binding in organic media comes about because of the small size of water (e.g., MW of H_2_O, THF, and toluene: 18.0, 72.1, and 92.1 g mol^–1^, respectively), its high cohesivity (strong internal hydrogen bond network), and its ability to form hydrogen bonds and van der Waals interactions with organic molecules. Together, these properties ensure that water can never be treated as purely a spectator species.

## Conclusions

We have detailed here the synthesis and conformation properties of TEEtOA **2**, a deep cavity cavitand with inherent conformational flexibility stemming from rim ethyl groups at its portal. We also report, using ITC and NMR spectroscopy, the binding affinities of a series of carboxylate guests to **2** and compare it to related deep-cavity cavitand TEMOA **1**. MD simulations reveal how the wetting of the pocket of **2** is controlled by the conformation of its rim ethyl groups and how guest binding follows a triggered wetting complexation, whereby the approach guest opens-up the pocket of the host, induces its wetting, and ultimately allows the formation of a hydrated host–guest complex (H·G·H_2_O). The observation of this mechanism adds to the growing list of mechanisms for host–guest complexation in aqueous solution, and based on this state-of-the-art, a general classification of mechanisms has been presented.

## Supporting Information Available

Synthesis and characterization of **1**, NMR and ITC analyses, and computational methods and results (PDF)
