# Supplementary material for: On the Nature of Guest Complexation in Water: Triggered Wetting–Water-Mediated Binding
Source: J Phys Chem B. 2022 Apr 19;126(16):3150–60. doi: 10.1021/acs.jpcb.2c00628 (PMC9059121; doi:10.1021/acs.jpcb.2c00628)
Supplement: Supplementary file 1 — jp2c00628_si_001.pdf [file jp2c00628_si_001.pdf]

Supplemental Information for:

On the Nature of Guest Complexation in Water: Triggered Wetting–Water-Mediated Binding

Paolo Suating[¶], Nicholas E. Ernst[¶], Busayo D. Alagbe[†], Hannah A. Skinner[¶], Joel T. Mague[¶], Henry S. Ashbaugh[†], and Bruce C. Gibb[¶*]

[¶] Department of Chemistry, Tulane University, New Orleans, LA 70118, USA
[†] Department of Chemical and Biomolecular Engineering, Tulane University, New Orleans, LA 70118, USA

Table of Contents

A. Hosts and Guests 5

B. Synthesis of tetra-endo-ethyl octa acid, **2**.......... 5

C. Summary of thermodynamic data.......... 34

D. Isothermal titration calorimetry (ITC): instrumentation.......... 35

E. ITC experimental parameters 36

F. ITC and NMR results 37

G. Crystal structure of 4-ethyl-3,5-dimethoxybenzoic acid, **c**.......... 45

H. Computational results.......... 51

I. References.......... 73

Figure S1: Hosts and guests used in this study. 5
Figure S2: ^{1}H NMR spectrum of 3,4,5-trimethoxybenzaldehyde dimethyl acetal, **a**, in acetone-*d*$_6$. 11
Figure S3: ^{13}C NMR spectrum of 3,4,5-trimethoxybenzaldehyde dimethyl acetal, **a**, in acetone-*d*$_6$. 12
Figure S4: ^{1}H NMR spectrum of 4-ethyl-3,5-dimethoxybenzaldehyde, **b**, in acetone-*d*$_6$. 13
Figure S5: ^{13}C NMR spectrum of 4-ethyl-3,5-dimethoxybenzaldehyde, **b**, in acetone-*d*$_6$. 14
Figure S6: ^{1}H NMR spectrum of 4-ethyl-3,5-dimethoxybenzoic acid, **c**, in acetone-*d*$_6$. 15
Figure S7: ^{13}C NMR spectrum of 4-ethyl-3,5-dimethoxybenzoic acid, **c**, in acetone-*d*$_6$. 16
Figure S8: ^{1}H NMR spectrum of 4-ethyl-3,5-dihydroxybenzoic acid, **d**, in acetone-*d*$_6$. Inset shows broad singlet indicative of a carboxylic acid. 17
Figure S9: ^{13}C NMR spectrum of 4-ethyl-3,5-dihydroxybenzoic acid, **d**, in acetone-*d*$_6$. 18
Figure S10: ^{1}H NMR spectrum of 2-ethyl-5(hydroxymethyl)benzene-1,3-diol, **e**, in acetone-*d*$_6$. 19
Figure S11: ^{13}C NMR spectrum of 2-ethyl-5(hydroxymethyl)benzene-1,3-diol, **e**, in acetone-*d*$_6$. 20
Figure S12: ^{1}H NMR spectrum of tetra-*endo*-ethyl octaethyl ester, **h**, in $CDCl_3$. 27
Figure S13: ^{1}H–^{1}H COSY NMR spectrum of tetra-*endo*-ethyl octaethyl ester, **h** in $CDCl_3$. 28
Figure S14: ^{13}C NMR spectrum of tetra-*endo*-ethyl octaethyl ester, **h**, in $CDCl_3$. Insets are expanded views of closely spaced signals in the aromatic and aliphatic regions. 29
Figure S15: ^{1}H NMR spectrum of 1 mM tetra-*endo*-ethyl octa acid, **2**, in 10 mM phosphate buffered D_2O, pD 11.45. 30
Figure S16: ^{1}H–^{1}H COSY NMR spectrum of 1 mM tetra-*endo*-ethyl octa acid, **2**, in 10 mM phosphate buffered D_2O, pD 11.45. 31
Figure S17: ^{13}C NMR spectrum of 10 mM tetra-*endo*-ethyl octa acid, **2**, in phosphate buffered D_2O, pD 11.45. 32
Figure S18: 2D ^{1}H–^{1}H NOESY spectrum of 10 mM tetra-*endo*-ethyl octa acid, **2**, in 10 mM phosphate buffered D_2O, pD 11.45. 33
Figure S19: DOSY NMR spectrum of 1 mM tetra-*endo*-ethyl octa acid, **2** in 10 mM phosphate buffered D_2O, pD 11.45. Diffusion constant, $D = 1.4634\times10^{-6}$ cm^2 s^{-1} 34
Figure S20: Variable-temperature ^{1}H NMR spectra of 1 mM tetra-*endo*-ethyl octa acid, **2**, in 10 mM phosphate buffered D_2O, pD 11.45. Temperatures are annotated on each spectrum. 35
Figure S21: DOSY NMR spectrum of 1mM tetra-*endo*-ethyl octa acid, **2**, complex with 100 equiv. **G1**, in 10 mM phosphate buffered D_2O, pD 11.45. Diffusion constant, $D = 1.5423\times10^{-6}$ cm^2 s^{-1} 36
Figure S22: DOSY NMR spectrum of 1 mM tetra-*endo*-ethyl octa acid, **2**, complex with 50 equiv. **G2**, in 10 mM phosphate buffered D_2O, pD 11.45. Diffusion constant, $D = 1.5734\times10^{-6}$ cm^2 s^{-1} 37
Figure S23: DOSY NMR spectrum of 1 mM tetra-*endo*-ethyl octa acid, **2**, complex with 100 equiv. **G4**, in 10 mM phosphate buffered D_2O, pD 11.45. Diffusion constant, $D = 1.4793\times10^{-6}$ cm^2 s^{-1} 38
Figure S24: DOSY NMR spectrum of 1 mM tetra-*endo*-ethyl octa acid, **2**, complex with 100 equiv. **G5**, in 10 mM phosphate buffered D_2O, pD 11.45. Diffusion constant, $D = 1.6765\times10^{-6}$ cm^2 s^{-1} 39
Figure S25: DOSY NMR spectrum of 1.0 mM tetra-endo-ethyl octa acid, **2**, dimerised with *n*-$C_{12}H_{26}$, in 10 mM phosphate buffered D_2O, pD 11.45. Diffusion constant, $D = 1.3717\times10^{-6}$ cm^2 s^{-1} 40
Figure S26: Plot of Δδ as a function of temperature from 30–60 °C. The blue line indicates the average of the Δδ for distal protons (H_j, H_g, H_f, H_e, H_a, H_l, H_m) ; the green and pink lines show the deviation of the $H_{c'}$ and $H_{c''}$ with increasing temperature. 46

Figure S27: ITC thermogram and 1:1 binding fit for TEMOA–G1 complexation. A 1.5 mM solution of G1 was titrated into a 0.15 mM solution of TEMOA equilibrated at 25 °C. Both host and guest were in 10 mM phosphate buffer, pH 11.5. 47
Figure S 28: ITC thermogram and 1:1 binding fit for TEEtOA–G1 complexation. A 20.0 mM solution of G1 was titrated into a 0.10 mM solution of TEEtOA equilibrated at 25 °C. Both host and guest were in 10 mM phosphate buffer, pH 11.5 47
Figure S29: ITC thermogram and 1:1 binding fit for TEMOA–G2 complexation. A 1.5 mM solution of G2 was titrated into a 0.15 mM solution of TEMOA equilibrated at 25 °C. Both host and guest were in 10 mM phosphate buffer, pH 11.5. 48
Figure S30: ITC thermogram and 1:1 binding fit for TEEtOA–G2 complexation. A 7.5 mM solution of G2 was titrated into a 0.10 mM solution of TEEtOA equilibrated at 25 °C. Both host and guest were in 10 mM phosphate buffer, pH 11.5. 48
Figure S31: ITC thermogram and 1:1 binding fit for TEMOA–G3 complexation. A 15 mM solution of G3 was titrated into a 0.15 mM solution of TEMOA equilibrated at 25 °C. Both host and guest were in 10 mM phosphate buffer, pH 11.5. 50
Figure S32: ITC thermogram and 1:1 binding fit for TEMOA–G4 complexation. A 1.5 mM solution of G4 was titrated into a 0.15 mM solution of TEMOA equilibrated at 25 °C. Both host and guest were in 10 mM phosphate buffer, pH 11.5. 51
Figure S33: ITC thermogram and 1:1 binding fit for TEEtOA–G4 complexation. A 20.0 mM solution of G4 was titrated into a 0.10 mM solution of TEEtOA equilibrated at 25 °C. Both host and guest were in 10 mM phosphate buffer, pH 11.5. 51
Figure S34: ITC thermogram and 1:1 binding fit for TEMOA–G5 complexation. A 1.2 mM solution of G5 was titrated into a 0.15 mM solution of TEMOA equilibrated at 25 °C. Both host and guest were in 10 mM phosphate buffer, pH 11.5. 52
Figure S35: ^{1}H NMR stack showing the addition of aliquots of 500 mM **G5** into a 1.0 mM solution of **TEEtOA**, **2**. Both solutions were in 10 mM pD 11.4 phosphate-buffered D_2O. 53
Figure S36: Host region between –1.0 – 1.6 ppm, and 3.0 – 4.6 ppm showing the shifting of **TEEtOA** host peaks H_b and $H_{c''}$ (see Figure S13) as a function of **G5**. 54
Figure S37: (**A**, **B**) Representative fitting curve and residuals of the titration of **G5** to TEEtOA tracking H_b. (**C**, **D**) Representative fitting curve and residuals of the titration of **G5** to **TEEtOA** tracking $H_{c''}$. Curve and residuals were calculated using the online BindFit software.[12, 13] 55
Figure S38: ORTEP thermal ellipsoid plot of the unit cell of **c** along the crystallographic *a* axis at a 50% probability level. 58
Figure S39: TEEtOA derivative **2'** used in calculations. Bonds rotated are highlighted in red. ..60
Figure S40: (top right to bottom left): Representative van der Waals structures of **2'** at different critical points in the energy profile as the four ethyl groups are simultaneously rotated out of the pocket: 0°, 80°, 160°, and 180°. The ethyl groups undergoing rotation are highlighted in pink. van der Waals structures were generated using the ePMV plugin for Cinema4D.[17] 60
Figure S41: Plot of relative energies of **2'** versus torsion angle φ. Critical points whose coordinates are listed below are highlighted in red. 61

A. Hosts and guests

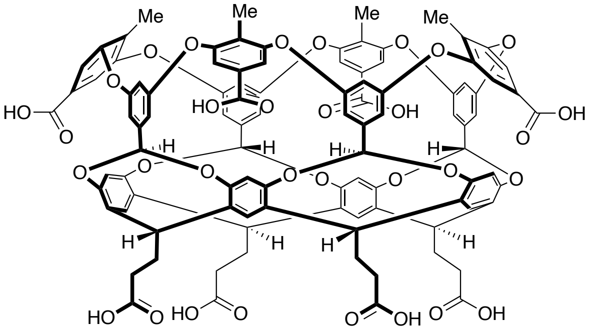

1, TEMOA

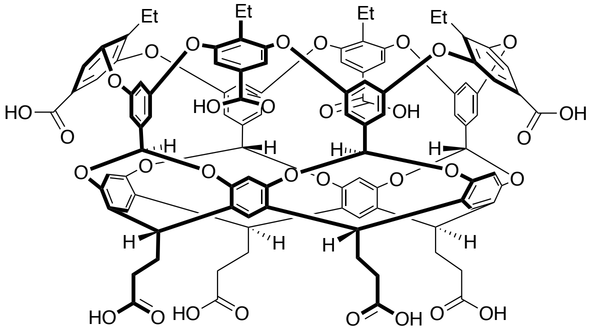

2, TEEtOA

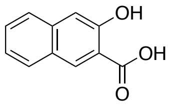

G1, 3-hydroxy-2-naphthoic acid

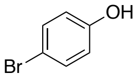

G2, *p*-bromophenol

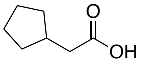

G3, cyclopentylacetic acid

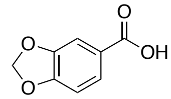

G4, piperonylic acid

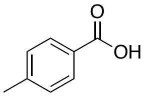

G5, *p*-toluic acid

Figure S1: Hosts and guests used in this study.

B. Synthesis of tetra-*endo*-ethyl octa acid (host 2)

General considerations

All reagents were purchased from either MilliporeSigma, Fisher Scientific, TCI America, or AK Scientific and were used without further purification unless otherwise specified. All reactions involving anhyd. solvents were performed under a blanket of dry N_2 gas. Anhyd. solvents were purchased from MilliporeSigma and, unless supplied with a Sure-Seal septum, stored with 3 Å molecular sieves activated at 250 °C overnight. Chromatography solvents were purchased from Fisher Scientific. Chloroform from Fisher Scientific contained up to 0.75% EtOH as preservative. Thin-layer chromatography was performed using 60G F_{254} glass-backed silica gel plates from MilliporeSigma. All flash column chromatography separations were performed using a dry load on a Teledyne Isco CombiFlash NextGen300+ instrument using SiliCycle SiliaSep silica cartridges, or Teledyne Isco RediSep Rf Gold cartridges for compound **h**. Degassing of solvents was performed by applying a vacuum on the solvent and replacing the atmosphere with dry N_2. The preparation of HCl-saturated EtOH was performed by bubbling HCl gas through 250 mL absolute EtOH for 5 mins immediately prior to use. Room temperature (rt) is used to describe

ambient laboratory temperatures ranging from 24–27 °C. All drying steps were performed under vacuum at or below 13 Pa and, in the case of high temperature drying, in an Abderhalden apparatus charged with the appropriate refluxing solvent (EtOAc at 77 °C, PhMe at 110 °C, and xylenes at 140 °C) as necessary.

All ^{1}H NMR spectra were recorded on either a Bruker Avance 500 (500.13 MHz) instrument or a Varian UNITY INOVA 400 (399.74 MHz) instrument operating at 25 °C, using residual $CHCl_3$ (δ 7.26 ppm), DMSO-d_5 (δ 2.50 ppm), acetone-d_5 (δ 2.05 ppm), or H_2O (δ 4.79 ppm) as an internal standard. All ^{13}C NMR spectra were recorded on either a Bruker Avance III 300 instrument (75.47 MHz ^{13}C), or a Varian UNITY INOVA 400 (100.51 MHz ^{13}C), or a Bruker Avance 500 (125.16 ^{13}C) operating at 25 °C using $CDCl_3$ (δ 77.16 ppm), acetone-d_6 (δ 29.84 ppm, 206.26 ppm), or DMSO-d_6 (δ 39.52 ppm) as an internal standard, and are broadband decoupled. 2D NMR spectra were recorded on a Bruker Avance 500 operating at 25 °C. NMR spectrum data were processed using MNova 14 (Mestrelab Research, S.L.). Multiplicity abbreviations are as follows: s – singlet; d – doublet; t – triplet; q – quartet; dd – doublet of doublets; dt – doublet of triplets; td – triplet of doublets; tt – triplet of triplets; br – broad signal; m – unresolved multiplet. Coupling constants *J* are reported in hertz as positive values regardless of their real individual signs. Solvents for NMR spectroscopy were purchased from Cambridge Isotope Laboratories.

All elemental analyses were performed offsite by Midwest Microlab. All melting point data were collected using a Stanford Research Systems Digimelt MPA160 Schmelzpunktbestimmungsapparat and are uncorrected.

Purification of copper(I) bromide

The purification[1] of copper(I) bromide and the synthesis of its dimethyl sulfide complex[2] used literature procedures. To a dry flask was added copper(I) bromide (50.0 g, 349 mmol) and glacial acetic acid (500 mL). The green suspension was stirred vigorously at rt under a blanket of N_2 for 24 h, filtered, the solids washed with absolute ethanol until the filtrate ran colourless, then dried under high vacuum at 110 °C for 24 h. The solids were transferred to a flame-dried, N_2-flushed flask and the solid contents cooled to –10 °C (1:1 ice/acetone). Dimethyl sulfide (300 mL) was then added dropwise via a pressure-equalising addition funnel over 30 min. and the suspension was allowed to stir at 0 °C for 30 mins. The resulting homogeneous, red-orange solution was heated to reflux (oil bath) for 24 h, after which time the solution was allowed to cool to rt. Hexanes (700 mL) was slowly poured onto the solution and the resulting suspension refrigerated for 4 h. The suspension was then filtered, and the solids washed with additional hexanes until the filtrate ran colourless. The solids were dried at rt overnight to afford the complex as a greyish-white crystalline powder (70.5 g, 98%). The complex was stored in a Dierite-charged desiccator under static vacuum until ready for use. Crystallographic data agree with the literature.[2]

3,4,5-trimethoxybenzaldehyde dimethyl acetal (a)

To a dry flask flushed with N_2 and fitted with a reflux condenser and an addition funnel charged with 3 Å molecular sieves (30 g)[3] was added 3,4,5-trimethoxybenzaldehyde (50.0 g, 255 mmol), anhyd. benzene (300 mL), trimethyl orthoformate (69.7 mL, 637 mmol, 2.55 equiv.), and *p*-toluenesulfonic acid hydrate (1.10 g, 6.37 mmol, 0.025 equiv.). The colour of the solution slowly changed from colourless, to purple, to yellow as the temperature increased from rt to reflux. The solution was stirred at reflux temperature (sand bath) for 48 h., after which the clear yellow solution was allowed to cool to rt. The cooled solution was diluted with 200 mL EtOAc, washed with 1 × 300 mL saturated aqueous $NaHCO_3$, 3 × 300 mL dH_2O, and 1 × 300 mL 20% w/w aqueous Na_2SO_4. The organic layer was then dried with anhyd. $MgSO_4$, filtered, and the solvent removed from the filtrate. The oily residue was then cooled to –78 °C (acetone/CO_2) to induce crystallization, and the solids dried at rt for 24 h. to afford **a** as an off-white solid (60.3 g, 98%). NMR spectroscopic data agree with the literature.[4] ^{1}H NMR (500.13 MHz, acetone-d_6) δ 3.27 (s,

6H), 3.72 (s, 3H), 3.82 (s, 6H), 5.30 (s, 1H), 6.72 (s, 2H). $^{13}C\{^1H\}$ NMR (100.51 MHz, acetone-d_6) δ 52.8, 56.3, 60.5, 103.8, 104.9, 135.0, 139.1, 154.2. mp 39.1–41.1 °C.

4-ethyl-3,5-dimethoxybenzaldehyde (b)

Compound **b** was synthesised according to a modification of a literature report.[5] To a flame-dried, N_2-flushed round-bottomed flask was added 80 mL unstabilized anhyd. THF and freshly cut sodium metal (3.0 g, 130 mmol, 3.15 equiv) pressed into leaves, and was allowed to stir at 23 °C for 15 minutes. To this was added dimethyl acetal **a** (10.0 g, 41.3 mmol) in one portion, and the mixture was allowed to stir at rt for 24 h during which the suspension went from colourless, to yellow, to deep red. The resulting deep red suspension was cooled to 0 °C (ice bath), and to which bromoethane (30 mL, 495 mmol, 3.81 equiv) was added over 30 minutes via a pressure-equalising addition funnel. The resulting yellow suspension was allowed to warm to rt and stirred for 24 h, after which the reaction was cooled to 0 °C for 1 h. The cooled solution was then quenched with 50 mL 6 M HCl added dropwise over 1 h (DANGER! H_2 released. Frequent venting required.) via a pressure-equalising addition funnel and allowed to stir at rt for 2 h. The organic solvent was then removed under reduced pressure, the remaining aqueous suspension was extracted with 3 × 75 mL EtOAc, and the combined organics washed sequentially with 1 × 50 mL satd. $NaHCO_3$, 1 × 100 mL dH_2O, and 1 × 50 mL 20% w/w aq Na_2SO_4. The organic layer was dried with anhyd. Na_2SO_4 and filtered. The solvent was removed under reduced pressure and the residue dried at rt for 12 h. The crude mixture was then subjected to column chromatography [gradient from 0–5% EtOAc in hexanes v/v, R_f = 0.46 (10% EtOAc in hexanes)] to afford **b** as a white micaceous solid (6.70 g, 84%) which was dried at rt for 24 h. NMR spectroscopic data agree with the literature.[5] ^1H NMR (399.74 MHz, acetone-d_6) δ 1.05 (t, J = 7.4 Hz, 3H), 2.70 (q, J = 7.4 Hz, 2H), 3.92 (s, 6H), 7.19 (s, 2H), 9.94 (s, 1H). $^{13}C\{^1H\}$ NMR (75.47 MHz, acetone-d_6) δ 13.8, 17.5, 56.5, 105.8, 128.3, 137.0, 159.5, 192.5. mp 72–73 °C.

4-ethyl-3,5-dimethoxybenzoic acid (c)

To a round-bottomed flask with magnetic stirrer was added **b** (2.4 g, 12.5 mmol) and THF (100 mL). To the solution was added 0.5 M aqueous Oxone (100 mL, 63 mmol, 5 equiv) and stirred for 48 h at rt, during which the triphasic suspension gradually took on an orange colour. The organic solvent was removed from the triphasic suspension under reduced pressure, and the resulting suspension was extracted with 100 mL EtOAc. The organic layer was then washed with 3 × 100 mL dH_2O and 1 × 100 mL 20% aq. Na_2SO_4. The organic layer was then dried with anhyd. Na_2SO_4, filtered, the solvent removed under reduced pressure, and the resulting residue dried at 77 °C. The fibrous solid was then dissolved in a minimum amount of boiling absolute EtOH, and slowly crystallised out of solution by the addition of ca. 15% boiling H_2O, and allowed to cool slowly to rt. The resultant needles were filtered, washed with ice-cold dH_2O, and dried at 77 °C to afford pure **c** (2.33 g, 90%). ^1H NMR (500.13 MHz, acetone-d_6) δ 1.04 (t, J = 7.4 Hz, 3H), 2.68 (q, J = 7.4 Hz, 2H), 3.88 (s, 6H), 7.27 (s, 2H), 11.2 (bs, 1H). $^{13}C\{^1H\}$ NMR (75.47 MHz, acetone-d_6) δ 13.9, 17.3, 56.3, 106.0, 126.6, 130.2, 158.9, 167.7. Calcd for $C_{11}H_{14}O_4$: C, 62.85; H, 6.71; O, 30.44. Found: C, 62.80; H, 6.71; O, 30.28. mp 194.1–195.5 °C.

Crystalline samples used for X-ray crystallography were obtained by layered diffusion of hexanes onto a solution of **c** in THF.

4-ethyl-3,5-dihydroxybenzoic acid (d)

To a flame-dried, N_2-flushed round-bottomed flask fitted with a magnetic stirrer was added **c** (2.0 g, 9.3 mmol), anhyd. CH_2Cl_2 (75 mL), and BBr_3 (3.6 mL, 37.3 mmol, 4 equiv). **N.B.**: Compound **c** is not immediately soluble in CH_2Cl_2. The solution was allowed to stir at rt for 18 h, after which the flask was opened to atmosphere for 10 mins. The solution was then cooled to 0 °C (ice bath) and 50 mL dH_2O was then added dropwise over 30 mins (CAUTION! $HBr_{(g)}$ released!) during which a tan solid precipitated. The organic solvent was then removed under reduced

pressure from the triphasic mixture, and the aqueous suspension was extracted with 3 × 30 mL Et_2O. To the combined organics were added anhyd. Na_2SO_4 and decolourising carbon, after which the suspension was stirred for 5 mins. The suspension was filtered through a pad of Celite 545, and the pad was flushed with an additional 50 mL Et_2O. The solvent was removed from the filtrate under reduced pressure and the solids dissolved in minimal THF. The solution was then triturated with 20 volumes of hexanes to precipitate the product. The suspension was refrigerated (3 °C) overnight, filtered, and the solids dried for 24 h at 77 °C to afford **d** as a fine white powder (1.6 g, 93%). ^1H NMR (399.74 MHz, acetone-d_6) δ 1.12 (t, J = 7.4 Hz, 3H), 2.71 (q, J = 7.4 Hz, 2H), 7.10 (s, 2H), 8.41 (s, 2H), 10.92 (bs, 1H). $^{13}C\{^1H\}$ NMR (75.47 MHz, acetone-d_6) δ 13.7, 17.4, 109.0, 123.6, 129.6, 156.8, 167.7 Calcd for $C_9H_{10}O_4$: C, 59.34; H, 5.53; O, 35.13. Found: C, 59.60; H, 5.63; O, 35.41. mp 133 °C (dec.).

2-ethyl-5(hydroxymethyl)benzene-1,3-diol (e)

To a flame-dried, N_2-flushed round-bottomed flask fitted with a magnetic stirrer and reflux condenser was added **d** (1.3 g, 7.1 mmol) unstabilized anhyd. THF (45 mL), and $(MeO)_3B$ (3.6 mL, 32.3 mmol, 4.5 equiv). The resulting solution was cooled to 0 °C for 10 mins, after which $BH_3 \cdot Me_2S$ complex (2.8 mL, 28.6 mmol, 4 equiv) was added dropwise over 10 mins (CAUTION! H_2 released. Frequent venting is required during addition.). The solution was stirred at 0 °C for 15 mins, after which the solution was heated to reflux (oil bath) for 24 h. The solution was then cooled to 0 °C (ice bath), opened to atmosphere, and MeOH (25 mL) was added over 5 mins to quench the reaction (CAUTION! H_2 released). The solvent was removed under reduced pressure, and 25 mL MeOH was added and removed under reduced pressure two additional times to facilitate the evaporation of the volatile borates. The resulting residue was dried for 1 h at rt and was then dissolved in a minimal amount of THF, triturated with 20 volumes of $CHCl_3$, and filtered. The resulting solids were dried overnight at 77 °C to afford **x** as a fine white powder (1.0 g, 85%). ^1H NMR (399.74 MHz, acetone-d_6) δ 1.08 (t, J = 7.5 Hz, 3H), 2.65 (q, J = 7.5 Hz, 2H), 3.89 (t, J = 6 Hz, 1H), 4.41 (d, J = 6 Hz, 2H), 6.38 (s, 2H), 7.91 (s, 2H). $^{13}C\{^1H\}$ NMR (75.47 MHz, acetone-d_6) δ 14.3, 17.1, 64.8, 106.0, 116.5, 142.0, 156.8. Calcd for $C_9H_{12}O_3$: C, 64.27; H, 7.19. Found: C, 64.49; H, 7.29. mp 153–156 °C

Crude Tetra-*endo*-ethyl octol, TEEtOctol (g)

To a flame-dried, N_2-flushed round bottomed flask fitted with a magnetic stirrer and reflux condenser was added sequentially pyridine (500 mL), octabromide **f** (10.0 g, 5.87 mmol), **e** (5.92 g, 35.2 mmol, 6 equiv), and anhyd. K_2CO_3 (9.73 g, 70.4 mmol, 12 equiv). The suspension was stirred and sparged with N_2 for 10 mins between additions to exclude atmospheric and dissolved O_2. To the suspension was then added $CuBr \cdot SMe_2$ (14.5 g, 70.4 mmol, 12 equiv), and the resulting deep green suspension was heated to a vigorous reflux for 21 d (sand bath) during which time the suspension took on a brown colour. The solvent was then removed under reduced pressure and the residue dried at rt for 10 mins. To the residue was then added THF (500 mL) and sonicated for 45 mins. The suspension was then filtered through a pad of Celite 545, and the pad flushed with an additional THF until the filtrate ran colourless. The solvent was removed from the filtrate under reduced pressure, and the residue dried at rt for 1 h. The residue was then suspended in 6 M aqueous HCl (150 mL) and sonicated for 1 h. The suspension was filtered, and the residue washed with dH_2O until the filtrate was pH neutral. The solids were dried at 140 °C overnight, sonicated in 150 mL EtOAc, filtered, and the dark-coloured solids washed with the same solvent until the filtrate ran colourless. The filtrate was allowed to stand at rt for 2 h during which time a tan micaceous solid precipitated out of solution. The solvent volume was then reduced to 50% under reduced pressure. The solids were filtered, then dried at 140 °C overnight to afford crude TEEtOctol **g** as a tan powder (3.92 g, 32% yield of crude **3**). Compound **g** was used in the next step without further purification.

Crude Tetra-*endo*-ethyl Octa Acid, TEEtOA (2)

To a flame-dried, N_2-flushed round-bottomed flask fitted with a magnetic stirrer was added crude **g** (1.4 g, 0.8 mmol) and DMA (100 mL). The solution was sparged for 5 mins, and to which was added *t*-BuOH (100 mL). The solution was again sparged for 5 mins, after which time $KMnO_4$ (3.5 g, 22.3 mmol, 28 equiv) was added in one portion. The deep purple solution was allowed to stir for 4 d at 60 °C, after which time the dark brown suspension was filtered, the residue taken up in 100 mL 4:1 dH_2O/DMA, sonicated for 20 mins, and filtered. The solvent was removed from the filtrate under reduced pressure and the residue dried at rt for 24 h. The residue was then suspended in conc. HCl (50 mL) and sonicated for 30 mins. The suspension was diluted with dH_2O (50 mL), sonicated for an additional 20 mins, filtered, and the solids washed with dH_2O until the filtrate was pH neutral. The solids were then dried overnight at 140 °C to afford crude **2** as a tan powder (1.1 g, 73% yield of crude **2**).

Tetra-*endo*-ethyl Octa Acid Octa Ethyl Ester, TEEtOEster (h)

To a 500 mL flame-dried, N_2-flushed round bottomed flask fitted with a magnetic stirrer and reflux condenser was added crude **2** (1.1 g, 0.6 mmol), HCl-saturated EtOH (90 mL), and $CHCl_3$ (140 mL). The light brown solution was heated to reflux (oil bath) under a blanket of N_2 for 4 d, after which the solvent was removed under reduced pressure and dried under high vacuum at rt for 4 h. The residue was taken up in absolute EtOH (15 mL), sonicated for 15 minutes, filtered, and dried at 110 °C overnight. The solids were subjected to column chromatography [gradient from 40% hexanes in $CHCl_3$ to 100% $CHCl_3$, R_f = 0.25 (5% acetone in $CHCl_3$)], the solvent removed and dried at rt. The compound was subjected to a second round of chromatographic separation using the same conditions but using Teledyne Isco RediSep Gold cartridges. The centre 50% fractions were collected, the solvent removed, and the resulting glassy, colourless solid was dissolved in minimal $CHCl_3$ and triturated with 20 volumes of hexanes to precipitate the product. The suspension was refrigerated overnight, filtered, and the solids dried at 140 °C overnight to afford **h** as a fine white powder. (0.98 g, 78%). ^{1}H NMR (500.13 MHz, $CDCl_3$) δ 0.58 (t, *J* = 7.5 Hz, 12H), 1.24 (t, *J* = 7.1 Hz, 12H), 1.41 (t, *J* = 7.1 Hz, 12 H), 2.17 (q, *J* = 7.5 Hz, 8H), 2.29 (t, *J* = 7.4 Hz, 8H), 2.54 (td, *J* = 7.7, 8.3 Hz, 8H), 4.13 (q, *J* = 7.1 Hz, 8H), 4.40 (q, *J* = 7.1 Hz, 8H), 4.43 (s, 4H), 4.77 (t, *J* = 8.2 Hz, 4H), 5.83 (s, 4H), 6.475 (d, *J* = 2.5 Hz, 8H), 7.08 (t, *J* = 2.5 Hz, 4H), 7.12 (s, 4H), 7.91 (s, 8H). $^{13}C\{^1H\}$ NMR (100.51 MHz, $CDCl_3$) δ 13.3, 14.3, 14.4, 18.1, 25.5, 32.5, 36.2, 60.8, 61.8, 104.9, 106.3, 109.3, 115.4, 122.0, 123.0, 131.0, 136.4, 136.6, 139.3, 153.9, 156.5, 161.4, 164.9, 172.9 Calcd for $C_{120}H_{112}O_{32}$: C, 69.76; H, 5.46. Found: C, 69.43; H, 5.36. mp >260 °C.

Pure Tetra-*endo*-ethyl Octa Acid, TEEtOA (2)

To a dry round-bottomed flask fitted with a magnetic stirrer was added octa-ester **h** (0.311 g, 0.150 mmol) and DMA (33 mL). The solution was stirred at 60 °C (oil bath) for 30 mins, after which 2 M aq. $LiOH{\cdot}H_2O$ (0.685 mL, 1.37 mmol, 9.10 equiv) was added dropwise over 5 mins. The solution gradually became turbid, and to the suspension was slowly added dH_2O (~10 mL) until the precipitate redissolved. The temperature of the solution was maintained at 60 °C (oil bath) for 24 h, after which the solution was allowed to cool to rt and the solvent was removed under reduced pressure. The residue was dried at rt overnight under vacuum, after which time conc. aq. HCl (20 mL) was added to the solids. The suspension was sonicated for 30 mins, after which the suspension was diluted with dH_2O (20 mL), sonicated for an additional 15 mins, then filtered. The solids were washed with additional portions of dH_2O until the filtrate was pH neutral. The solids were then dried at 140 °C overnight, taken up in 15 mL anhyd. acetone, then sonicated for 5 mins. The suspension was refrigerated overnight (3 °C), filtered, and the solids dried at 140 °C for 48 h to afford pure tetra-*endo*-ethyl octa acid **2** as a fine white powder (0.27 g, 97%). ^{1}H NMR (500.13 MHz, 10 mM in pD 11.45 phosphate buffer in D_2O) δ –0.35 (m, 12 H), 2.22 (t, *J* = 7.3, 8H), 2.52 (m, 8 H), 2.61` (m, 8H), 4.15 (bs, 4H), 4.66 (t, *J* = 8.8 Hz, 4H), 5.84 (s, 4H), 6.57

(s, 8H), 7.17 (s, 4H), 7.54 (s, 4H), 7.84 (s, 8H). $^{13}C\{^1H\}$ NMR (75.47 MHz, D_2O) δ 11.9, 17.3, 26.1, 35.4, 36.4, 105.3, 106.2, 110.2, 113.9, 122.4, 123.8, 133.0, 137.1, 137.6, 138.3, 153.1, 155.7, 161.9, 172.9, 182.3. Calcd for $C_{104}H_{80}O_{32}·4H_2O·HCl$: C, 64.05; H, 4.60 Found: C, 64.33; H, 4.21. mp >260 °C.

Optional purification of tetra-*endo*-ethyl octol (g) via the octa-acetate (g-OAc)

To a flame-dried, N_2-flushed round-bottomed flask fitted with a magnetic stirrer was added **g** (0.500 g, 0.29 mmol) and acetic anhydride (20 mL, 212 mmol, 732 equiv.). The suspension was stirred at 110 °C (oil bath) for 24 h, during which time the mixture became homogenous. The solution was then allowed to cool to rt, and the solvent was removed under reduced pressure. The residue was dried under high vacuum at 110 °C for 4 h. The residue was subjected to column chromatography [gradient from 0% EtOAc in hexanes to 10 % EtOAc in hexanes (R_f = 0.15)], and the relevant fractions dried at 110 °C overnight. The glassy residue was then taken up in hexanes and sonicated for 10 minutes, and the resulting powder was filtered and dried at 110 °C to give **g-OAc** as a fine white powder (0.341 g, 57%). ^1H NMR (500.13 MHz, $CDCl_3$) δ 0.52 (t, *J* = 7.4 Hz, 12H), 1.66 (tt, *J* = 7.1, 6.6 Hz, 8H), 2.055 (s, 12H), 2.061 (s, 12H), 2.15 (q, *J* = 7.4 Hz, 8H), 2.29 (dt, *J* = 8.2, 6.6 Hz, 8H), 4.14 (t, *J* = 6.6 Hz, 8H), 4.44 (s, 4H), 4.80 (t, *J* = 8.2 Hz, 4H), 5.20 (s, 8H), 5.90 (s, 8H), 6.486 (s, *J* = 2.3 Hz, 8H), 7.047 (t, *J* = 2.3 Hz, 4H), 7.09 (s, 4H), 7.26 (s 8H). $^{13}C\{^1H\}$ NMR (125.77 MHz, $CDCl_3$) δ 13.4, 17.5, 20.85, 20.97, 26.73, 26.87, 36.0, 63.8, 64.9, 104.7, 106.1, 109.4, 115.2, 121.0, 121.7, 130.6, 136.65, 136.68, 138.9, 154.0, 156.3, 161.4, 170.6, 171.0. Calcd for $C_{120}H_{112}O_{32}·H_2O$: C, 69.15; H, 5.51 Found: C, 69.41; H, 5.50. mp >260 °C.

Hydrolysis of octa-acetate (g-OAc) to pure tetra-*endo*-ethyl octol (g)

To a dry flask flushed with N_2 and fitted with a magnetic stirrer was added **g-OAc** (0.150 g, 0.073 mmol) and DMA (10 mL). The solution was allowed to stir at 70 °C (oil bath) for 30 mins, after which time 2 M aq. $LiOH·H_2O$ (1.52 mL, 3.05 mmol, 42 equiv.) was added in one portion. The resulting suspension was allowed to stir for 15 mins after which dH_2O was added in 0.5 mL portions every 2 mins until the precipitate dissolved (~3 mL) and the resulting homogenous mixture was allowed to stir overnight. The solution was allowed to cool to rt, the solvent was removed under reduced pressure, and the residue was dried at rt under high vacuum for 3 h. The residue was then taken up in 1 M aq. HCl (25 mL) and sonicated for 30 minutes. The suspension was filtered, and the solids washed with dH_2O until the eluting filtrate was pH neutral. The solids were then dried at 140 °C under high vacuum to afford pure **g** as a white powder (0.115 g, 92%). ^1H NMR (500.13 MHz, DMSO-d_6) δ –0.04 (t, *J* = 7.6 Hz, 12H), 1.41 (tt, *J* = 7.0, 6.6 Hz, 8H), 2.29 (q, *J* = 7.6 Hz, 8H), 2.40 (dt, *J* = 7.9, 6.2 Hz, 8H), 3.48 (dt, *J* = 6.0, 5.5 Hz, 8H), 4.42 (s, 4H), 4.50 (t, *J* = 4.8 Hz, 4H), 4.56 (t, *J* = 8.1 Hz, 4H), 4.58 (d, *J* = 5.4 Hz, 8H), 5.44 (t, *J* = 5.9 Hz, 4H), 5.73 (s, 8H), 6.434 (d, *J* = 1.5 Hz, 8H), 7.146 (t, *J* = 2.0 Hz, 4H), 7.26, (s, 8H), 7.71 (s, 4H). $^{13}C\{^1H\}$ NMR (125.76 MHz, DMSO-d_6) δ 13.0, 16.9, 26.0, 30.6, 36.2, 60.2, 61.7, 104.5, 105.6, 108.8, 114.3, 119.1, 124.9, 128.1, 137.0, 138.6, 143.6, 153.2, 155.2, 161.3. Calcd for $C_{104}H_{96}O_{24}·2HCl·H_2O$: C, 68.60; H, 5.54. Found: C, 68.95; H, 5.50. mp >260 °C.

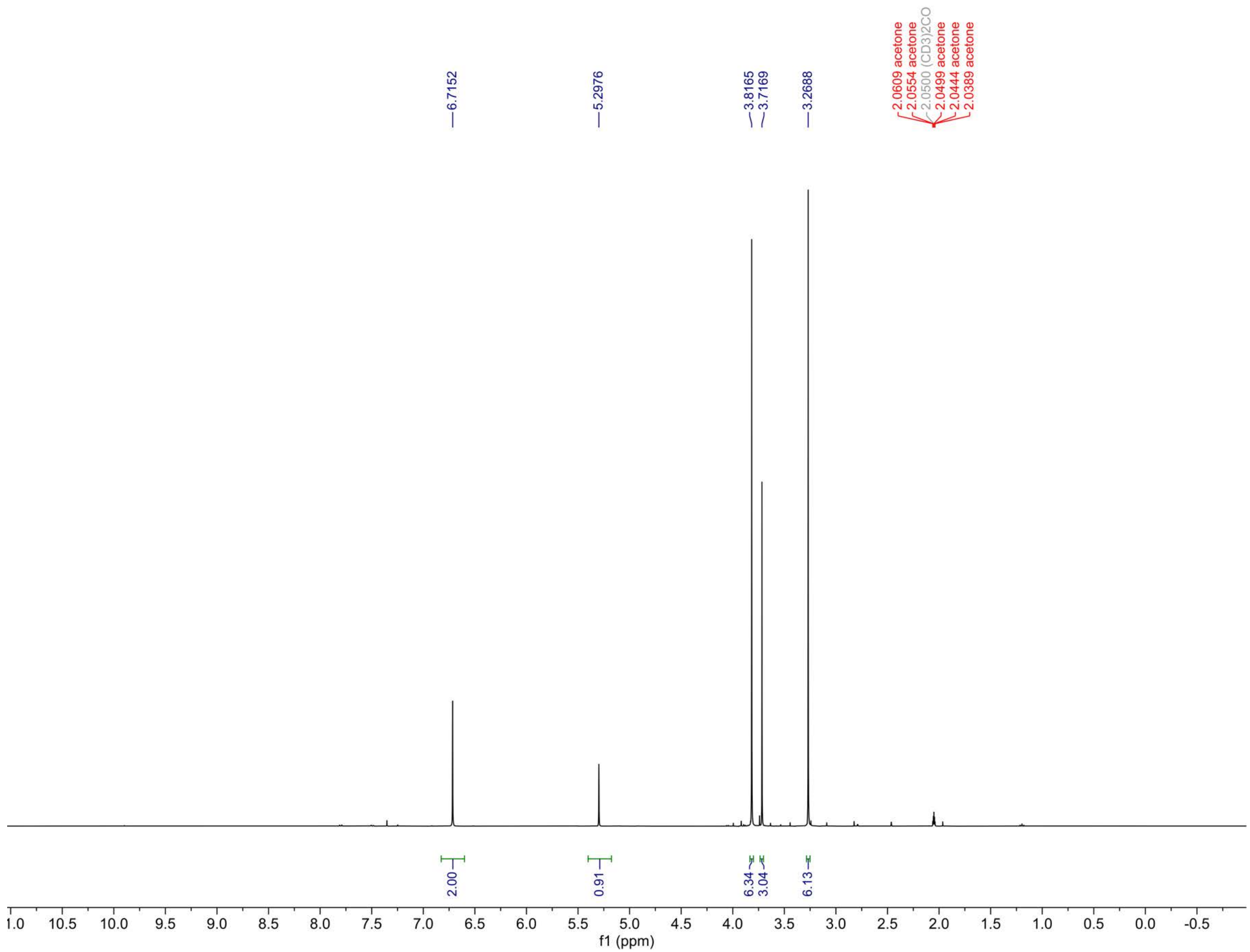

Figure S2: ^{1}H NMR spectrum of 3,4,5-trimethoxybenzaldehyde dimethyl acetal, **a**, in acetone-d_6.

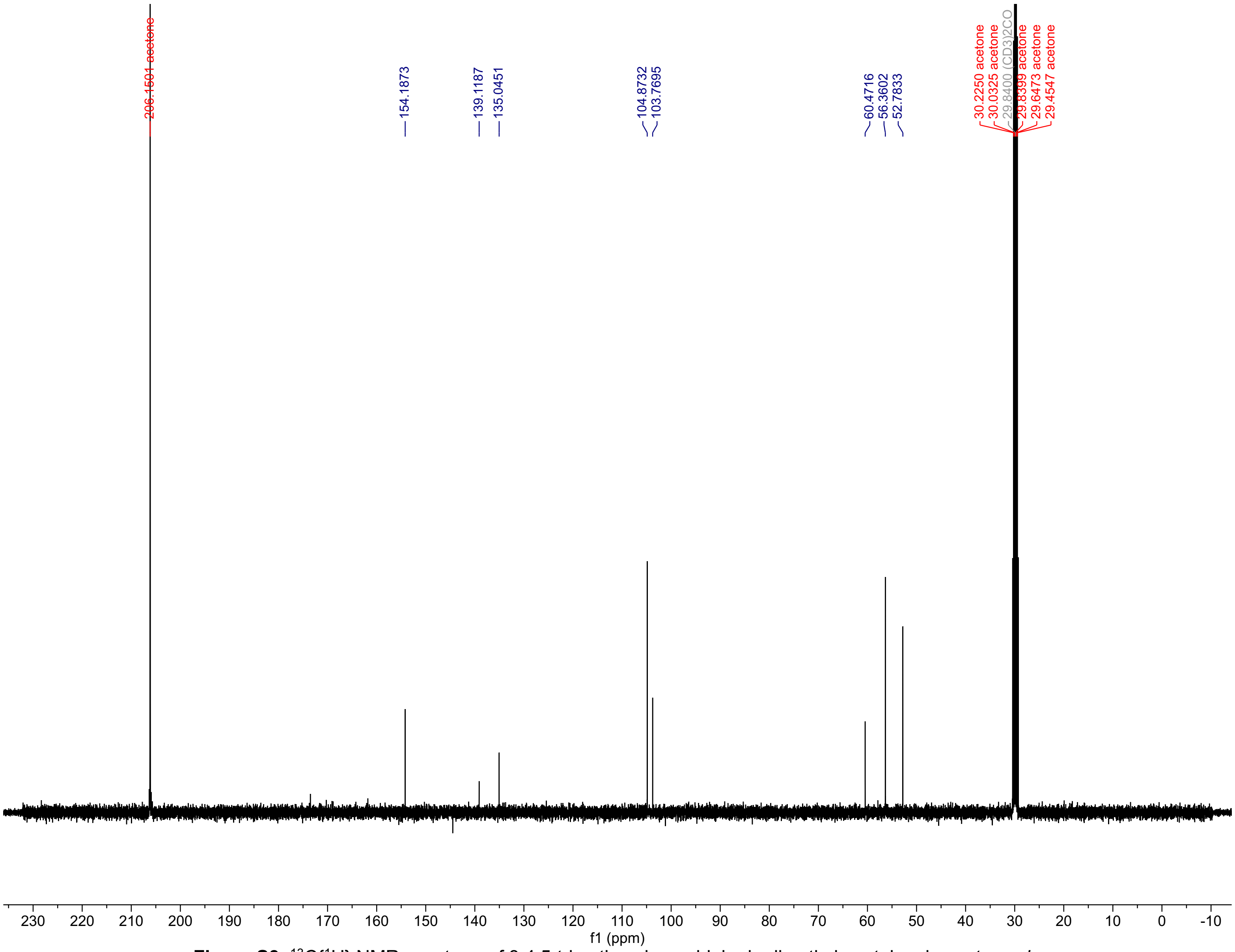

Figure S3: $^{13}C\{^1H\}$ NMR spectrum of 3,4,5-trimethoxybenzaldehyde dimethyl acetal, **a**, in acetone-d_6.

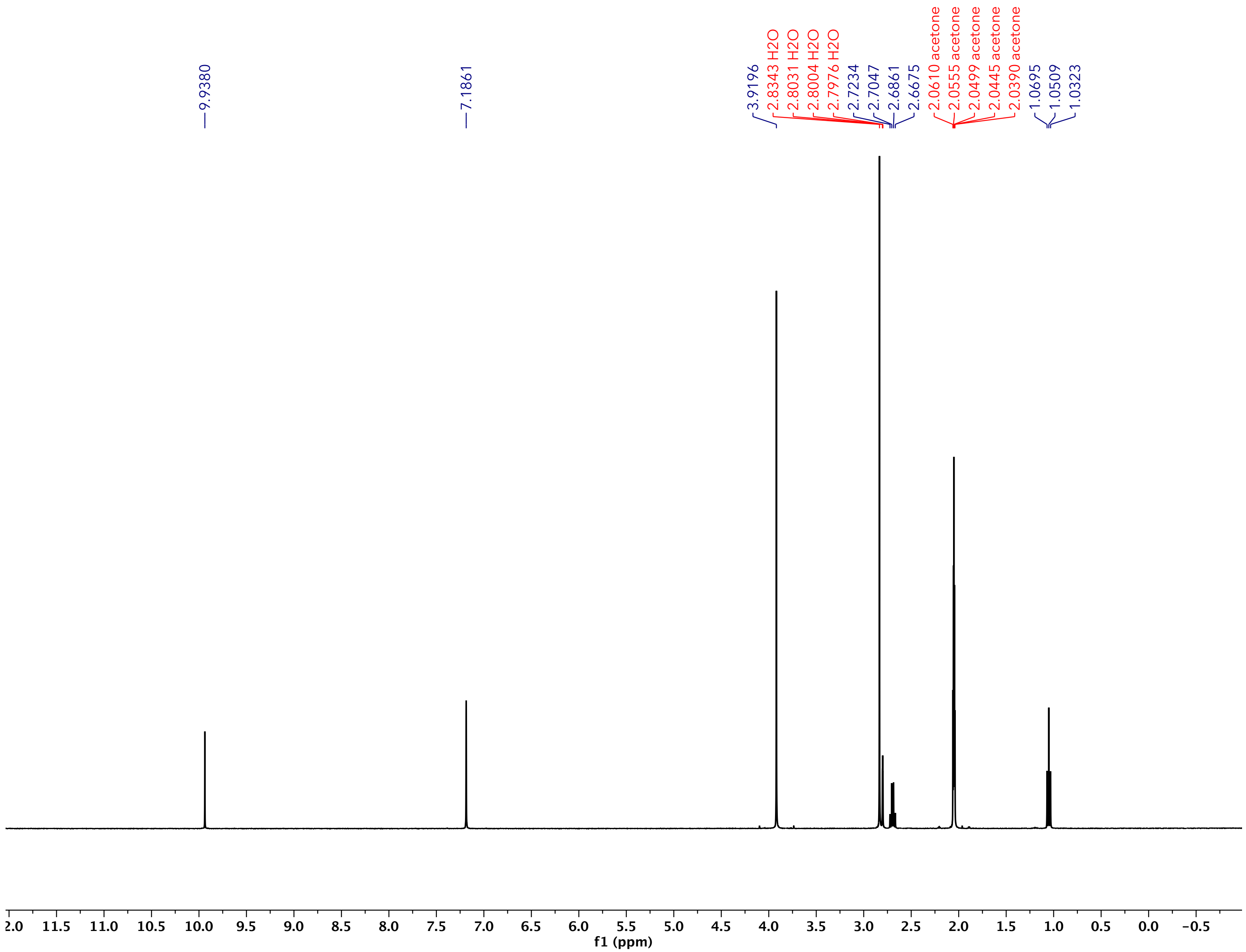

Figure S4: ^{1}H NMR spectrum of 4-ethyl-3,5-dimethoxybenzaldehyde, **b**, in acetone-d_6.

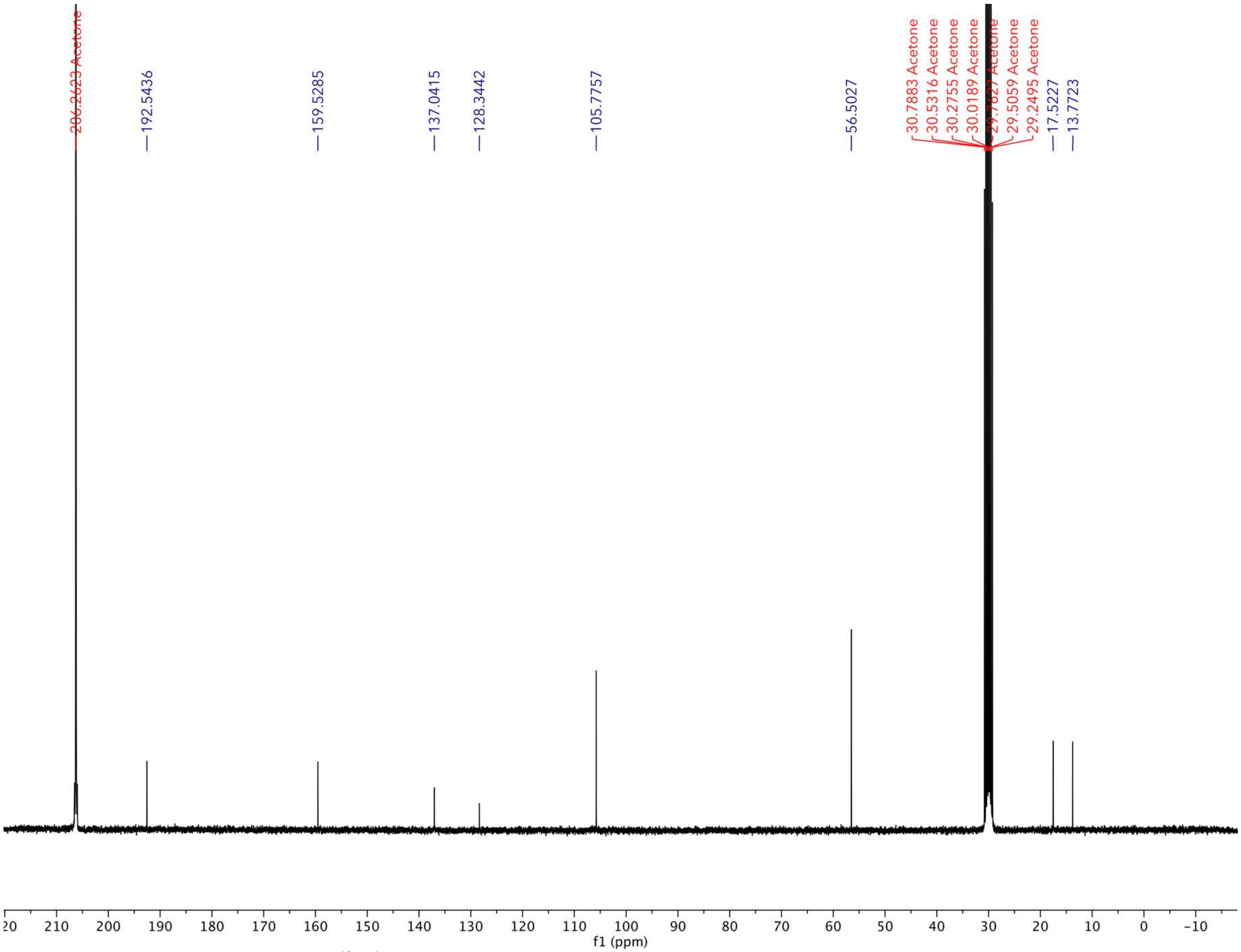

Figure S5: $^{13}C\{^{1}H\}$ NMR spectrum of 4-ethyl-3,5-dimethoxybenzaldehyde, **b**, in acetone-d_6.

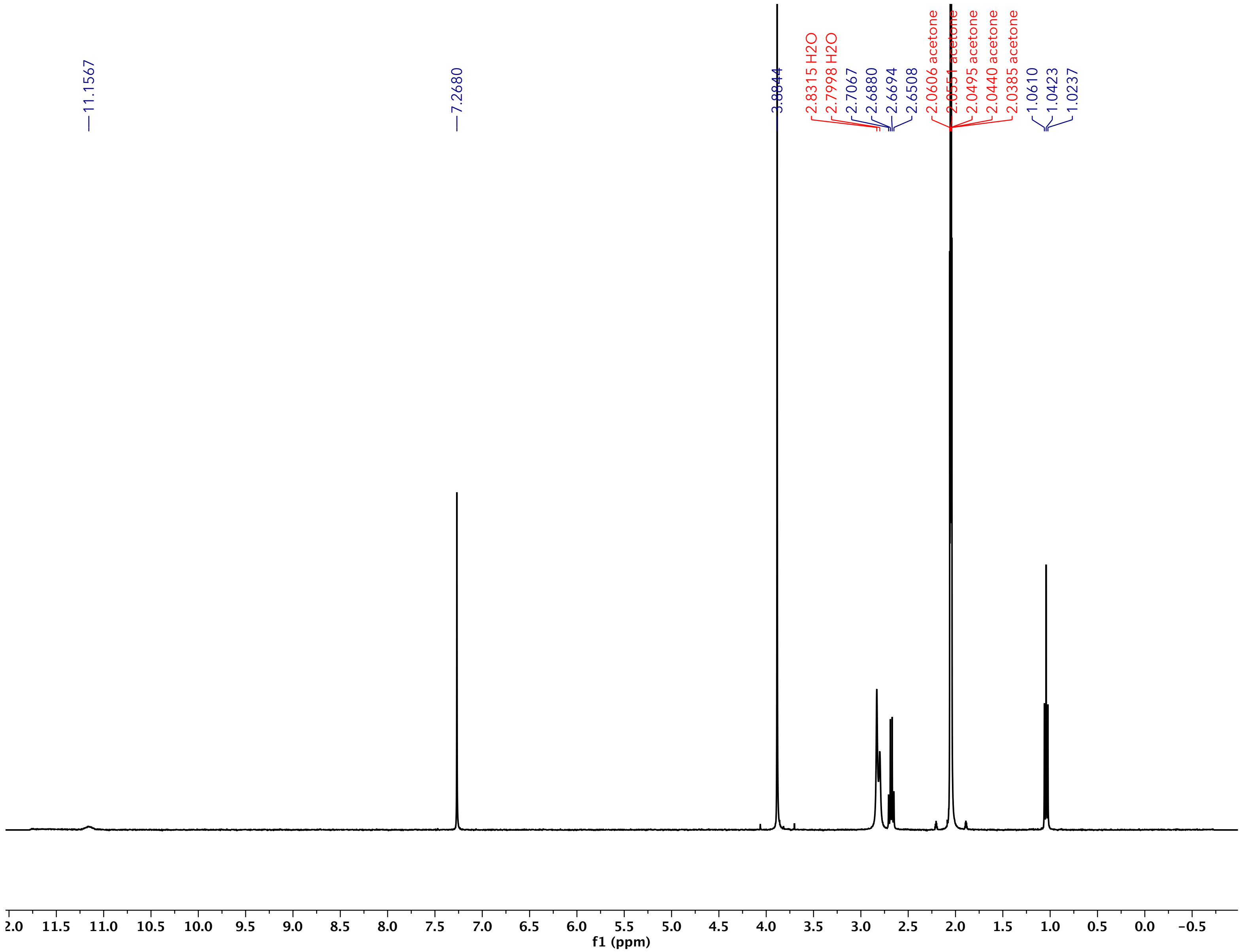

Figure S6: ^{1}H NMR spectrum of 4-ethyl-3,5-dimethoxybenzoic acid, **c**, in acetone-d_6.

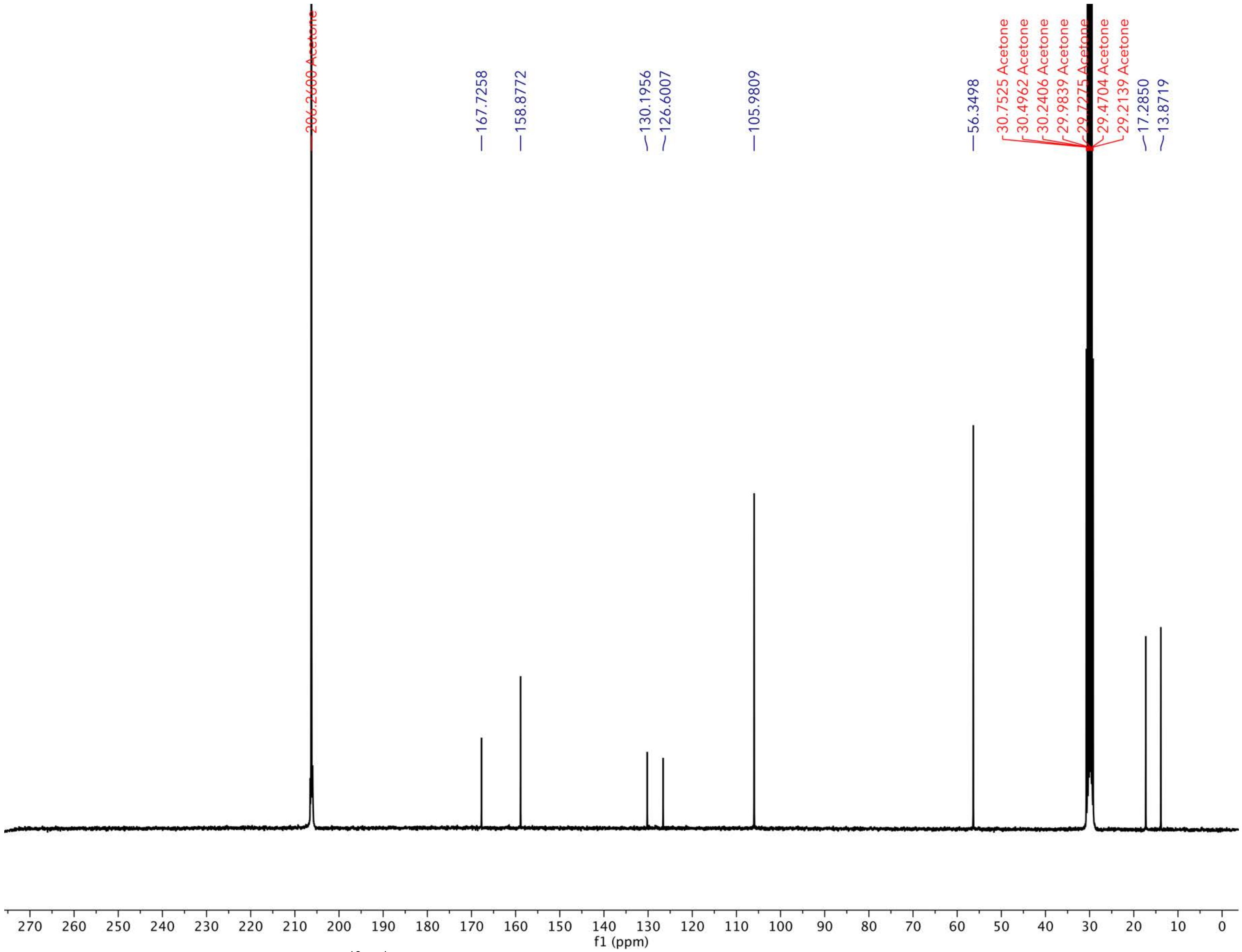

Figure S7: $^{13}C\{^1H\}$ NMR spectrum of 4-ethyl-3,5-dimethoxybenzoic acid, **c**, in acetone-d_6.

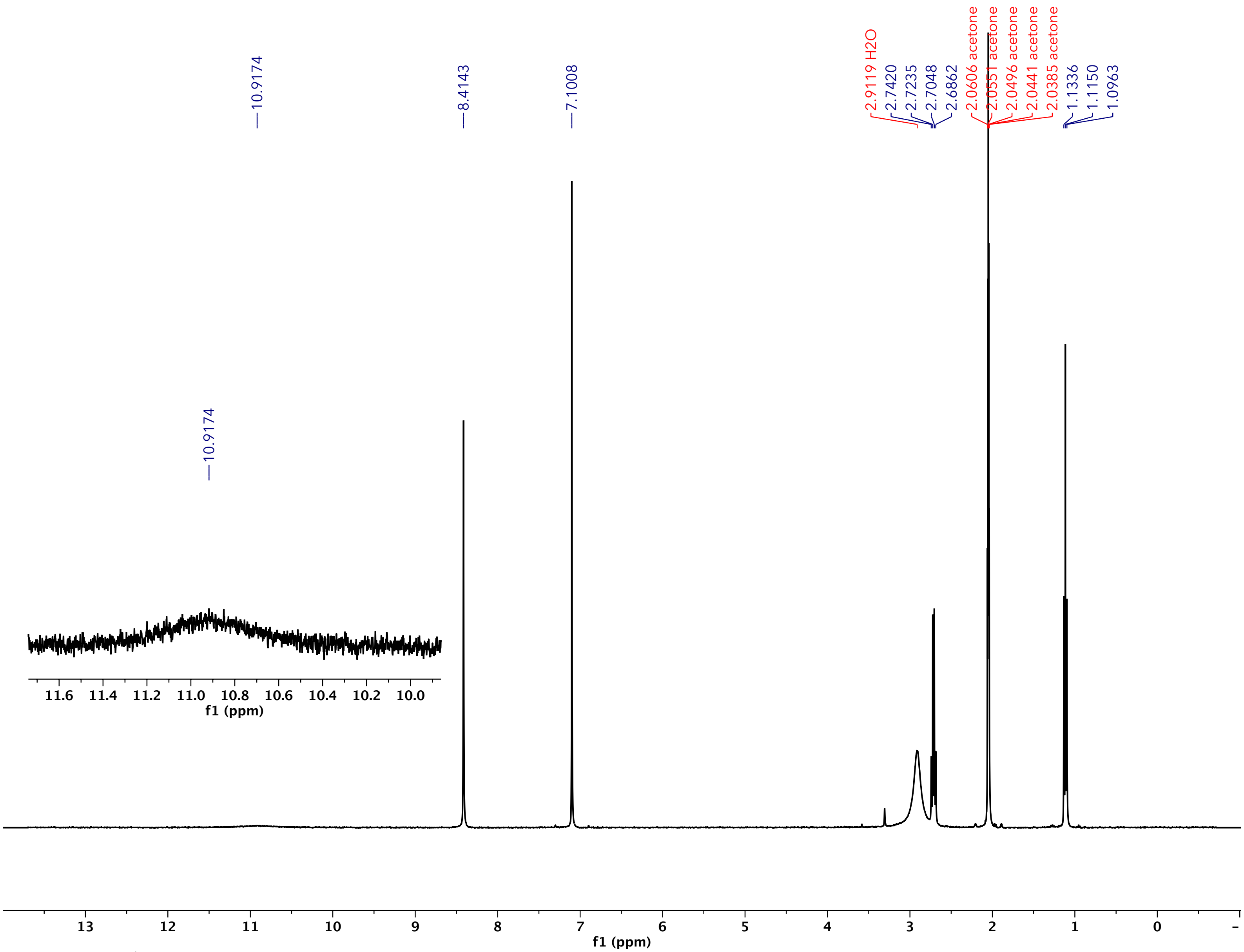

Figure S8: ^{1}H NMR spectrum of 4-ethyl-3,5-dihydroxybenzoic acid, **d**, in acetone-d_6. Inset shows broad singlet indicative of a carboxylic acid.

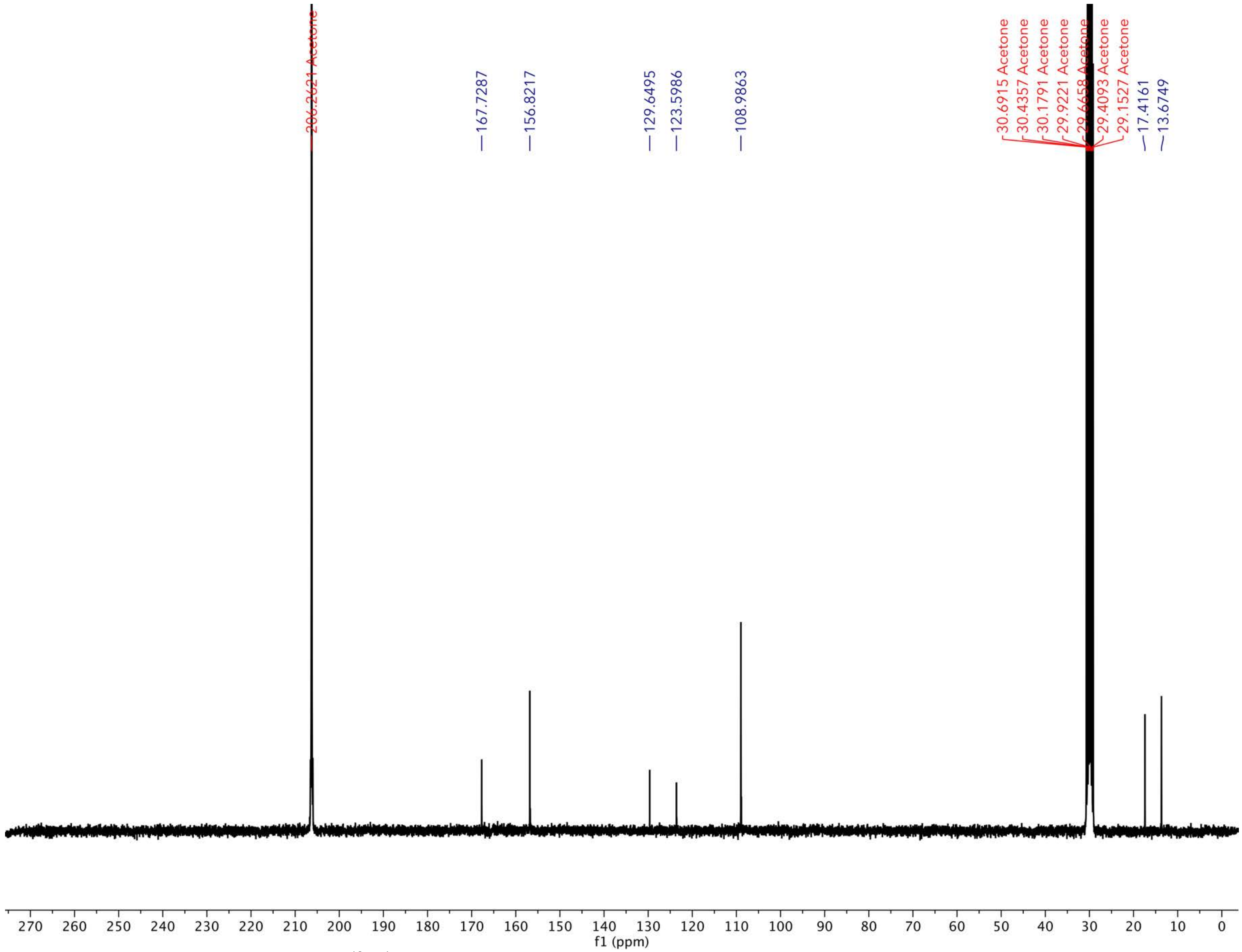

Figure S9: $^{13}C\{^1H\}$ NMR spectrum of 4-ethyl-3,5-dihydroxybenzoic acid, **d**, in acetone-d_6.

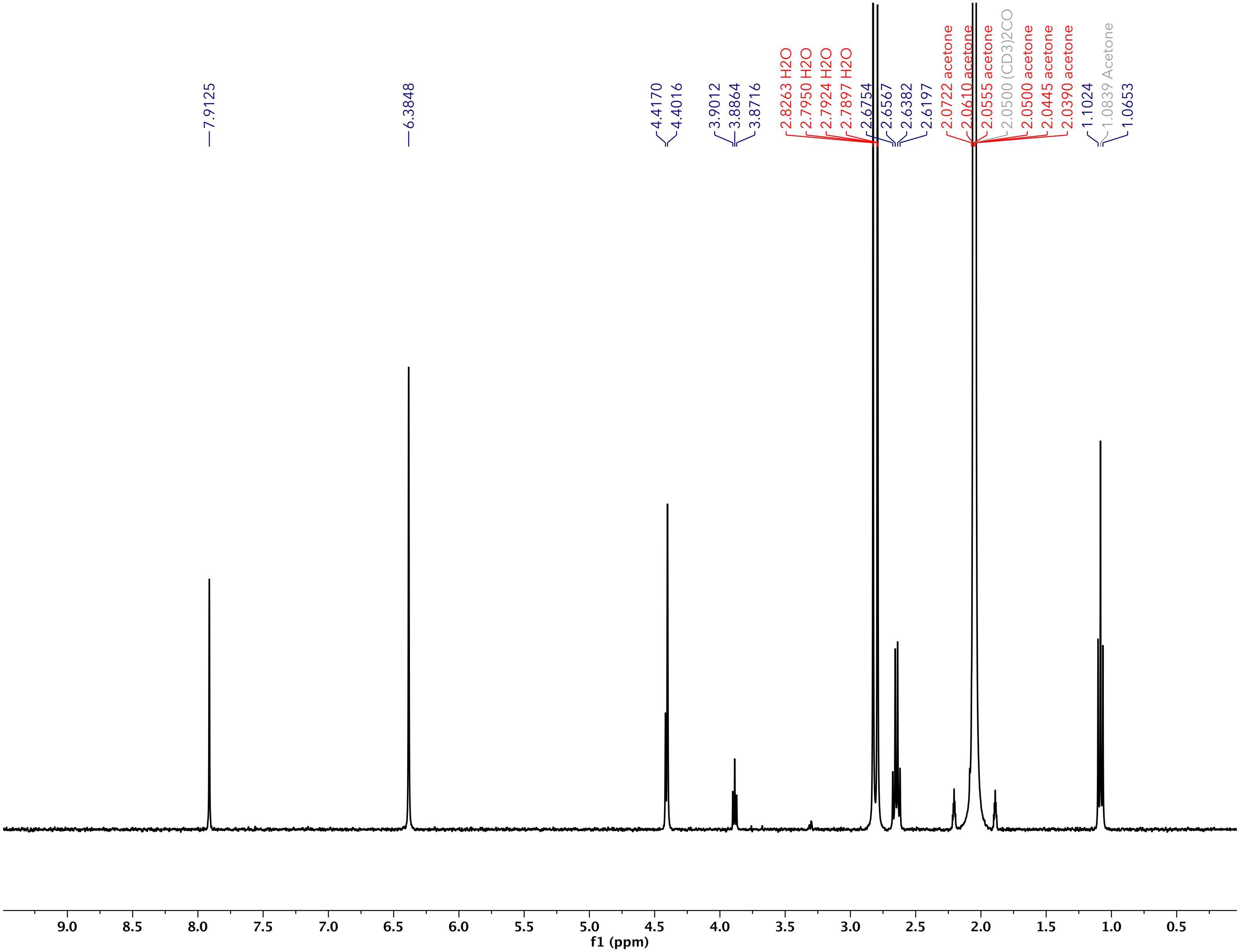

Figure S10: ¹H NMR spectrum of 2-ethyl-5(hydroxymethyl)benzene-1,3-diol, **e**, in acetone-d_6.

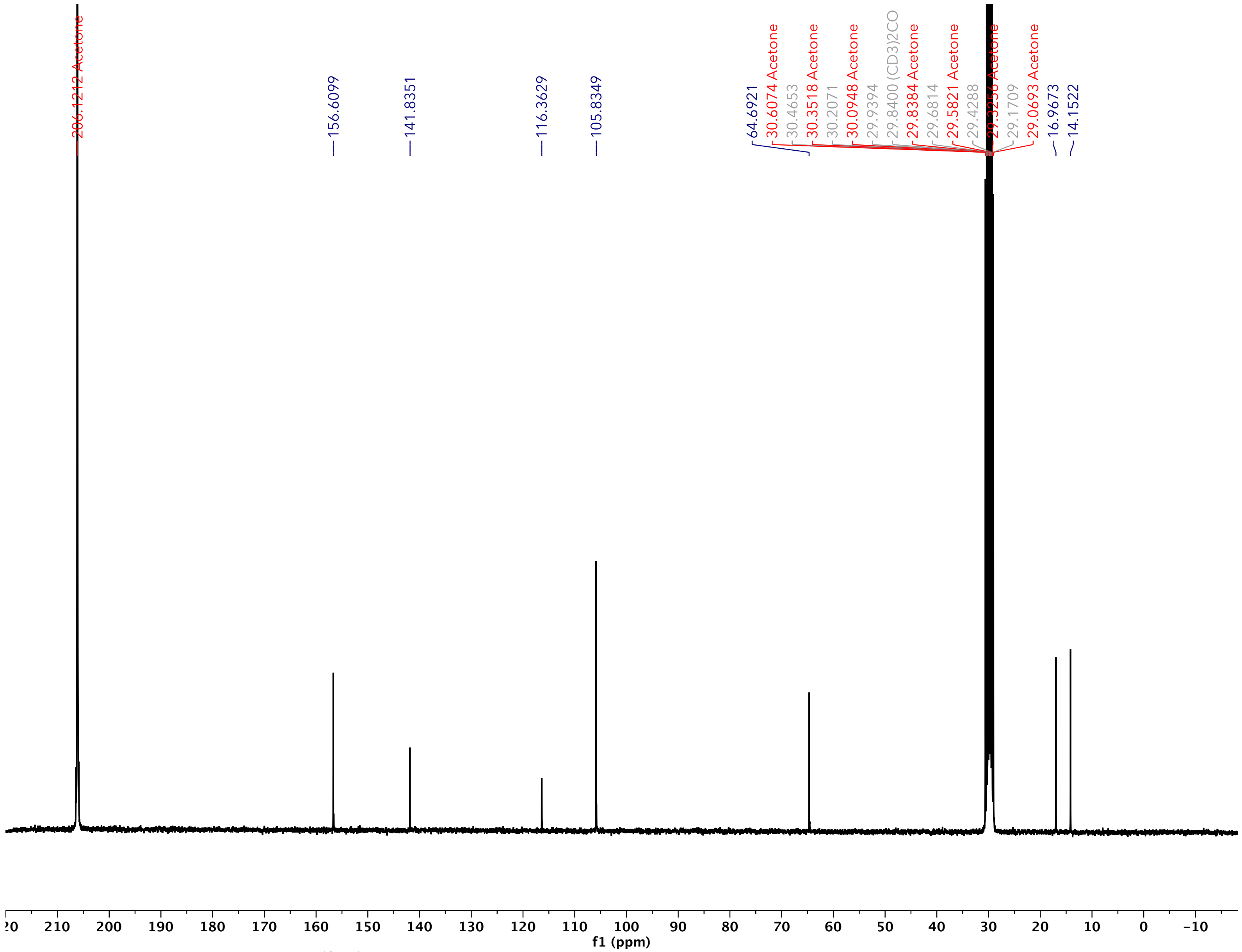

Figure S11: $^{13}C\{^{1}H\}$ NMR spectrum of 2-ethyl-5(hydroxymethyl)benzene-1,3-diol, **e**, in acetone-d_6.

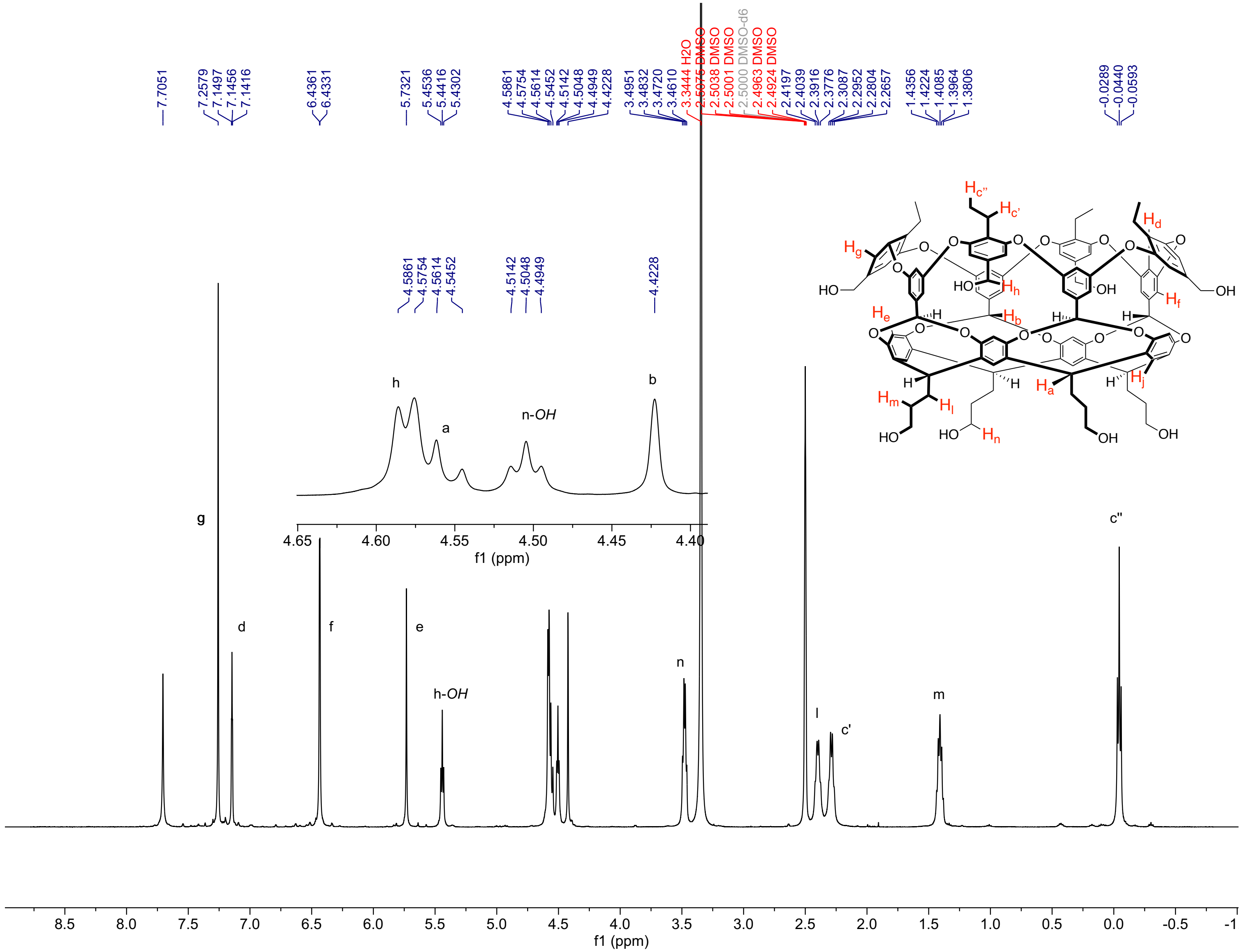

Figure S12: ^{1}H NMR spectrum of TEEtOctol **g** in DMSO-d_6. Inset shows crowded region between 4.40–4.65 ppm.

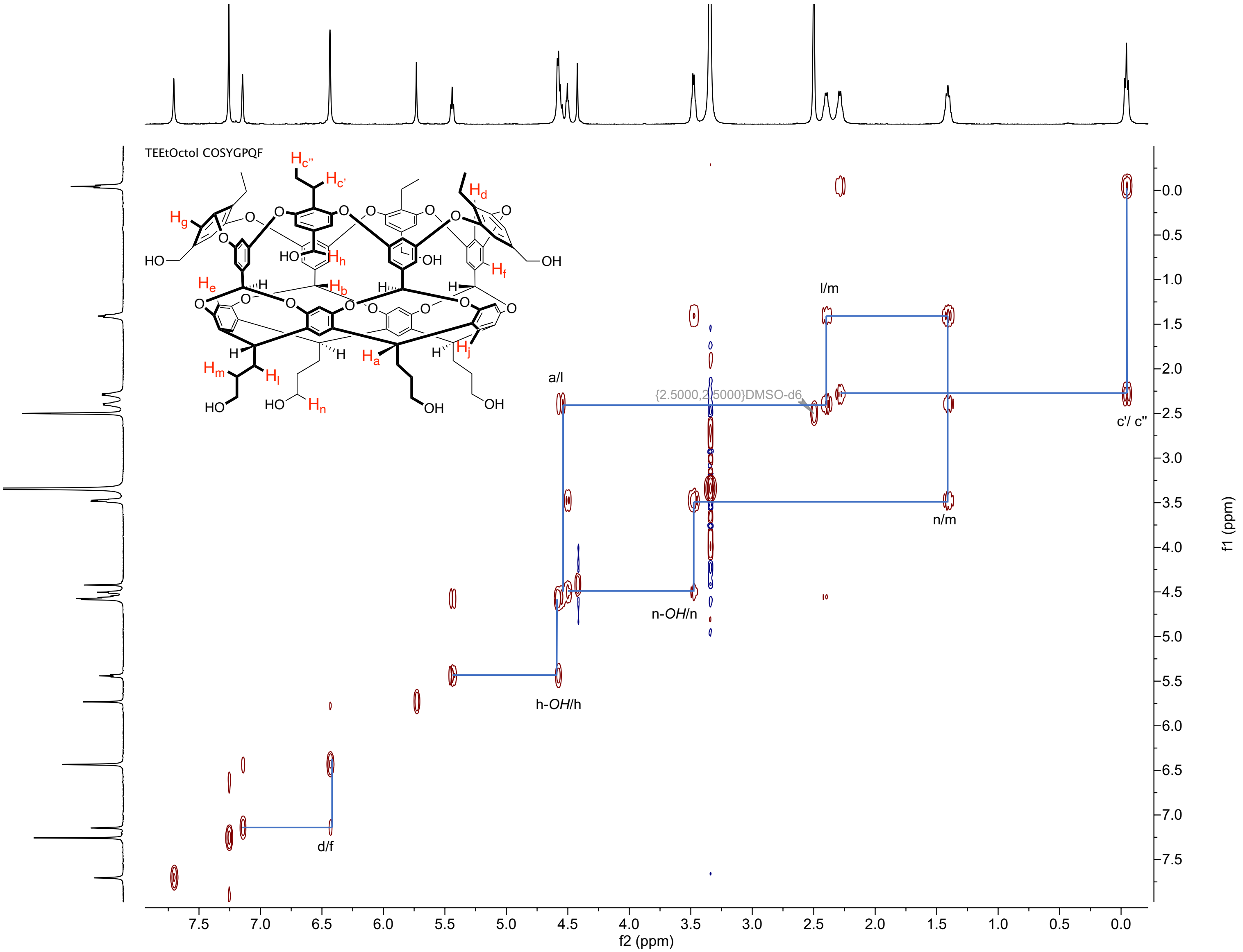

Figure S13:^{1}H–^{1}H COSY NMR spectrum of TEEtOctol **g** in DMSO-d_6.

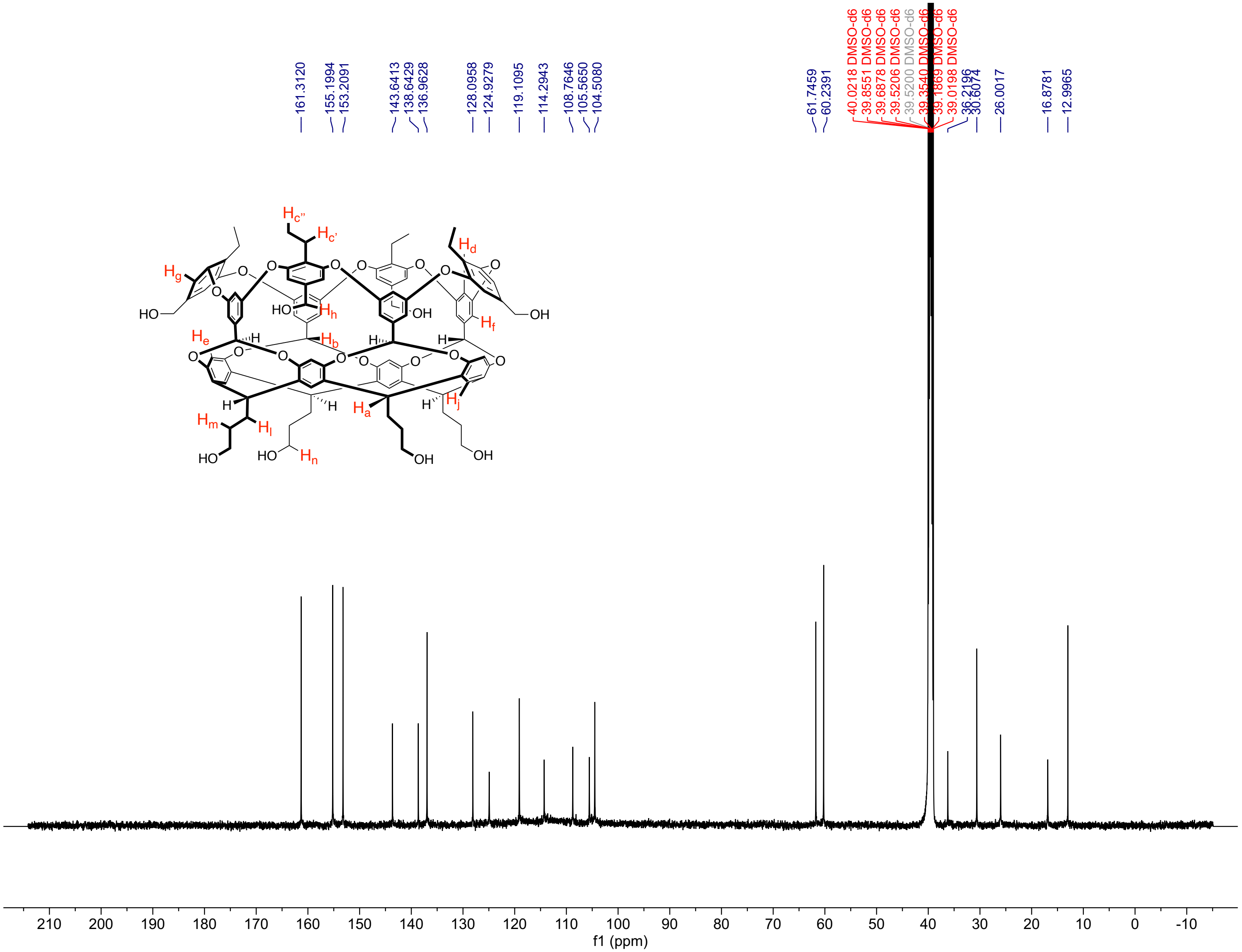

Figure S14: $^{13}C\{^{1}H\}$ NMR spectrum of TEEtOctol **g** in DMSO-d_6.

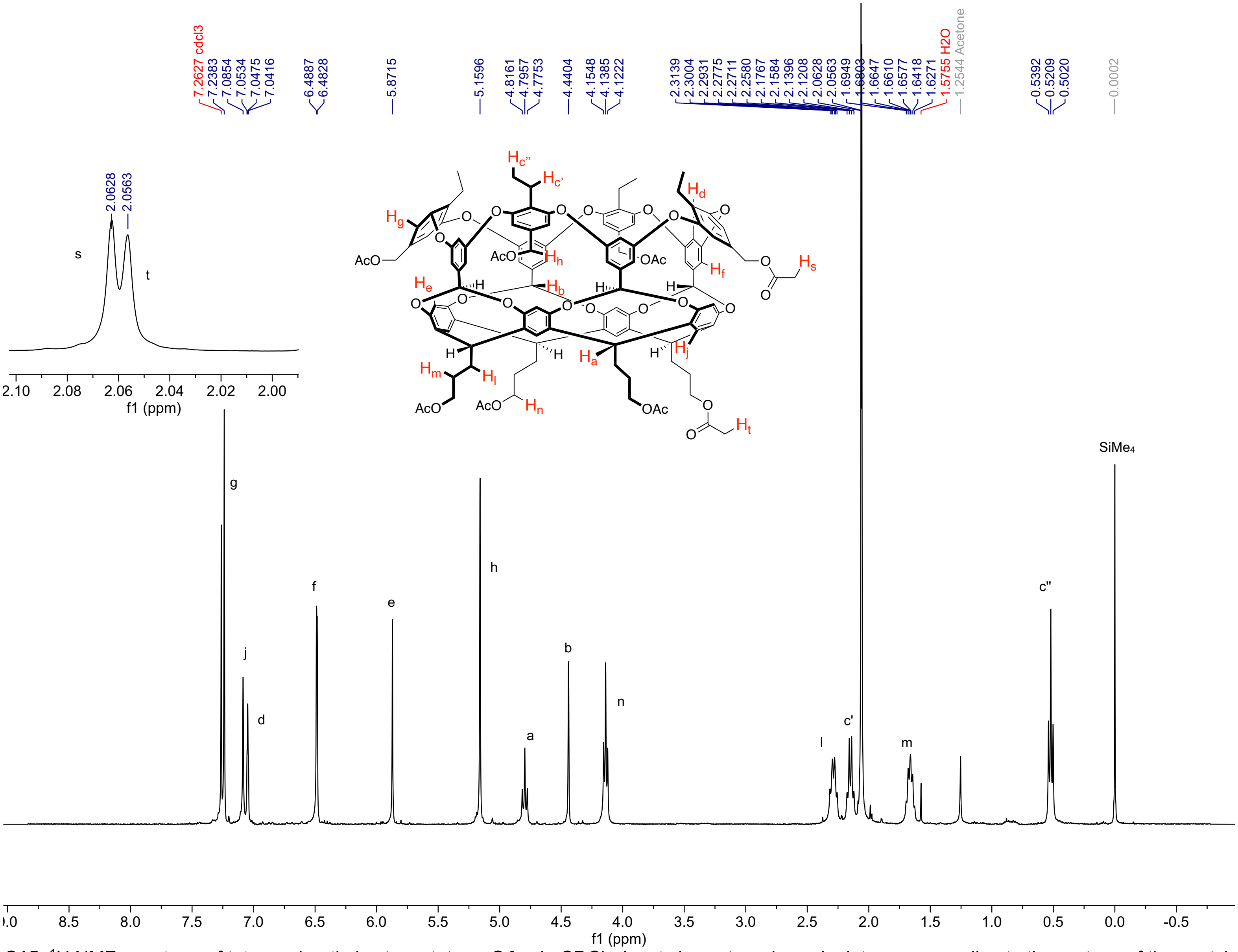

Figure S15: ^{1}H NMR spectrum of tetra-endo-ethyl octaacetate, **g-OAc**, in $CDCl_3$. Inset shows two close singlets corresponding to the protons of the acetyl groups.

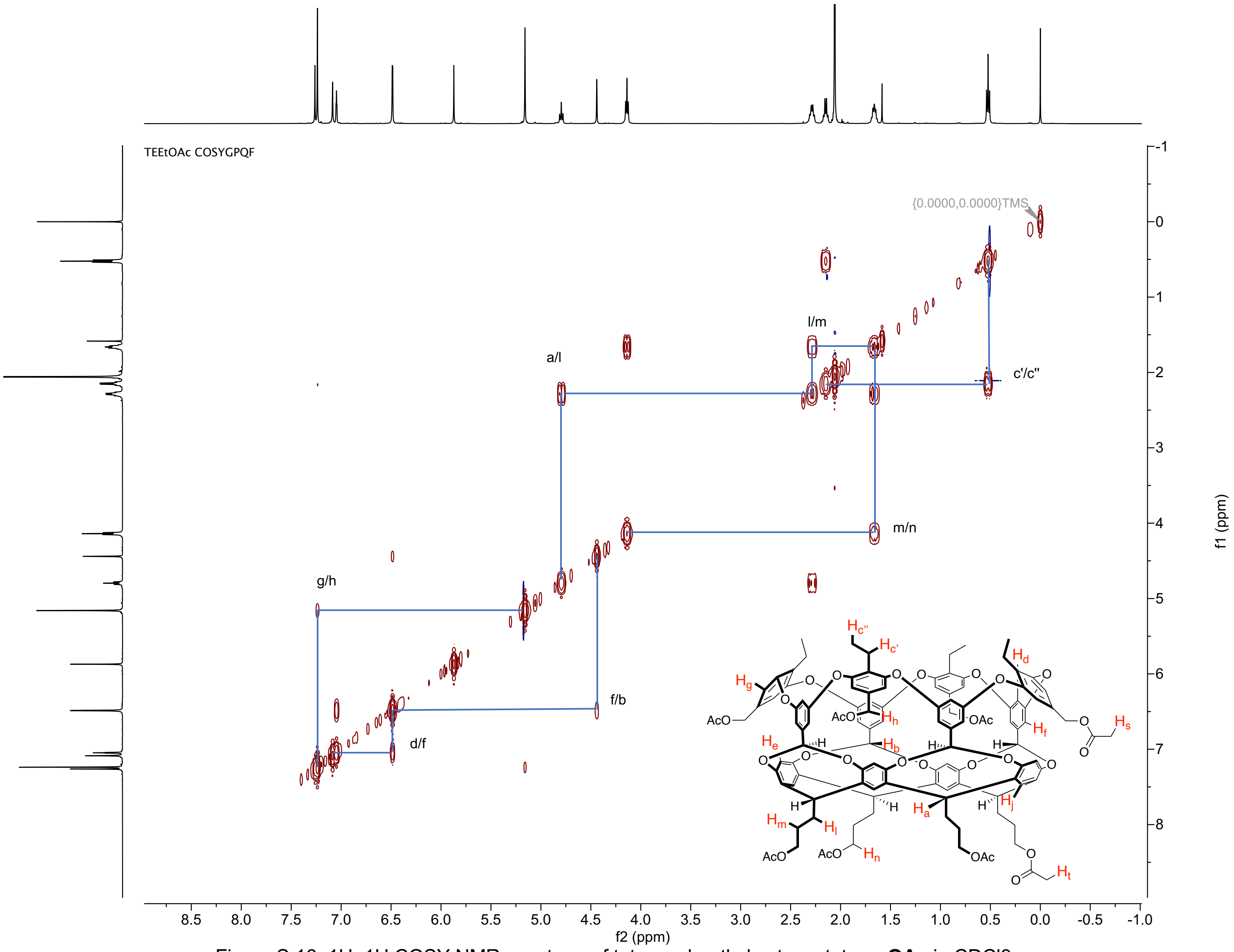

Figure S 16: 1H–1H COSY NMR spectrum of tetra-endo-ethyl octaacetate, **g-OAc** in CDCl3.

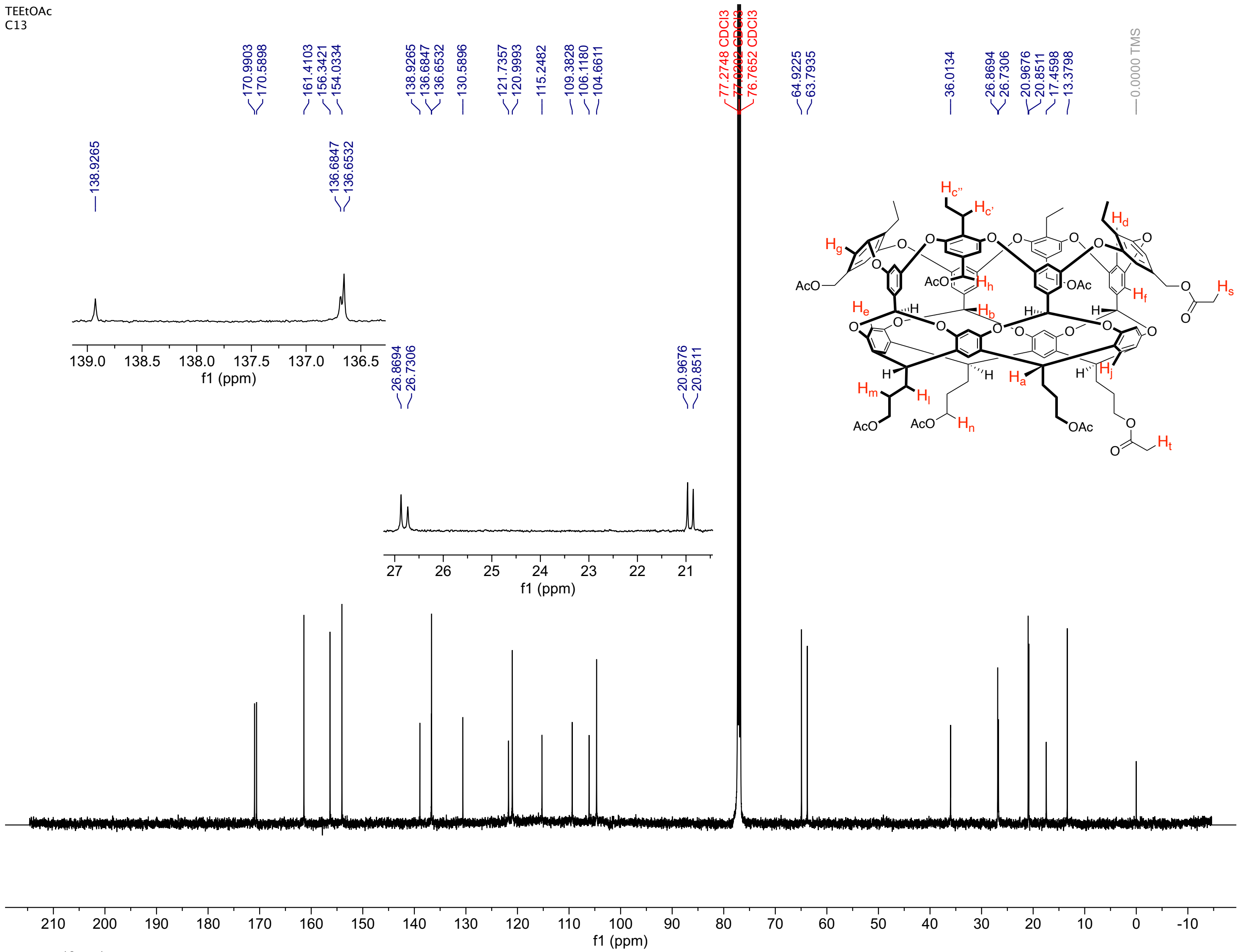

Figure S 17: $^{13}C\{^1H\}$ NMR spectrum of octaacetate **g-OAc** in $CDCl_3$. Inset shows regions of tightly-spaced signals from 136–139 ppm, and 20–27 ppm.

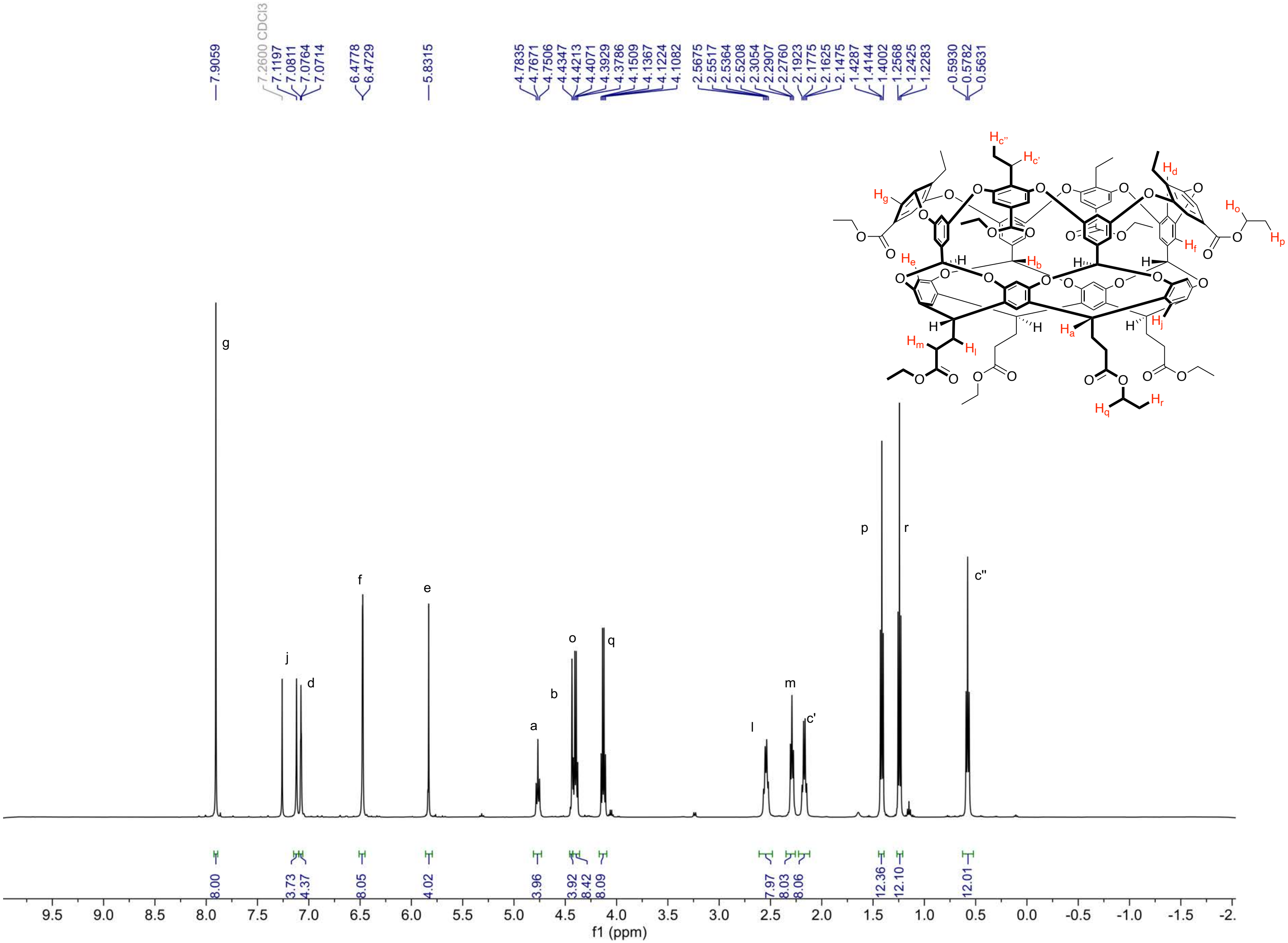

Figure S18: ^{1}H NMR spectrum of tetra-*endo*-ethyl octaethyl ester, **h**, in $CDCl_3$.

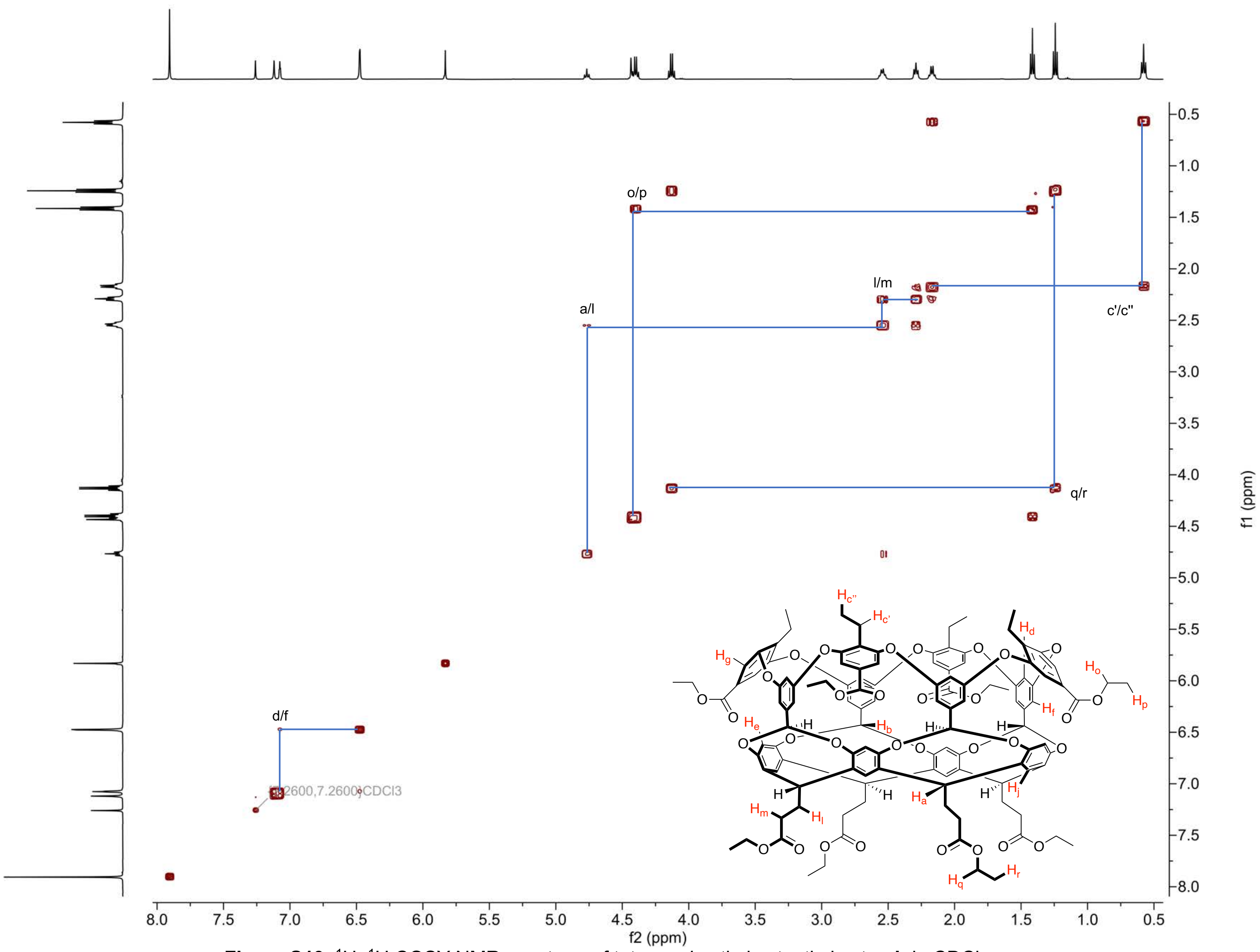

Figure S19: ^{1}H–^{1}H COSY NMR spectrum of tetra-*endo*-ethyl octaethyl ester, **h** in $CDCl_3$.

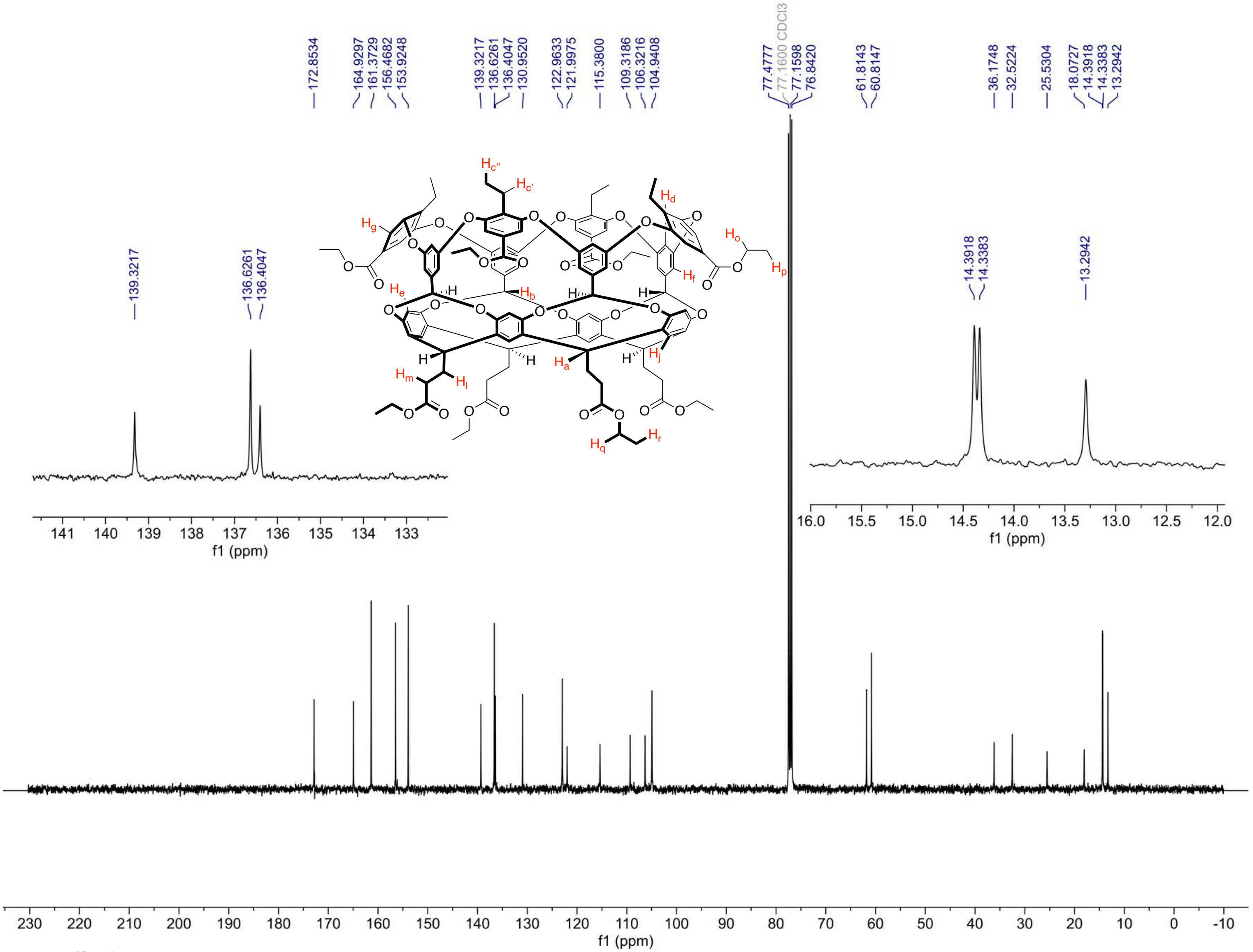

Figure S20: $^{13}C\{^{1}H\}$ NMR spectrum of tetra-*endo*-ethyl octaethyl ester, **h**, in $CDCl_3$. Insets are expanded views of tightly-spaced signals in the aromatic and aliphatic regions.

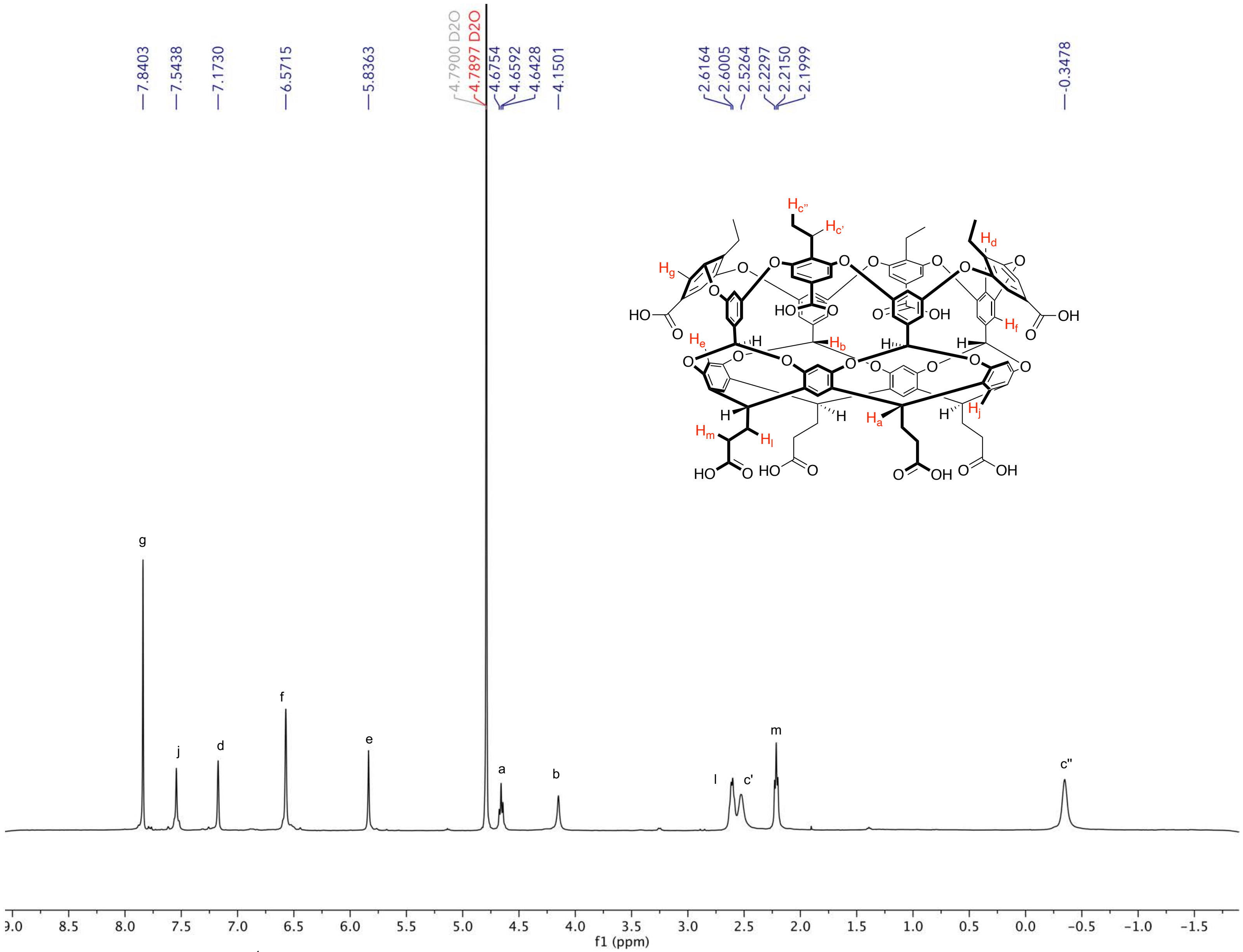

Figure S21: ^{1}H NMR spectrum of 1 mM tetra-*endo*-ethyl octa acid, **2**, in 10 mM phosphate buffered D_2O, pD 11.45.

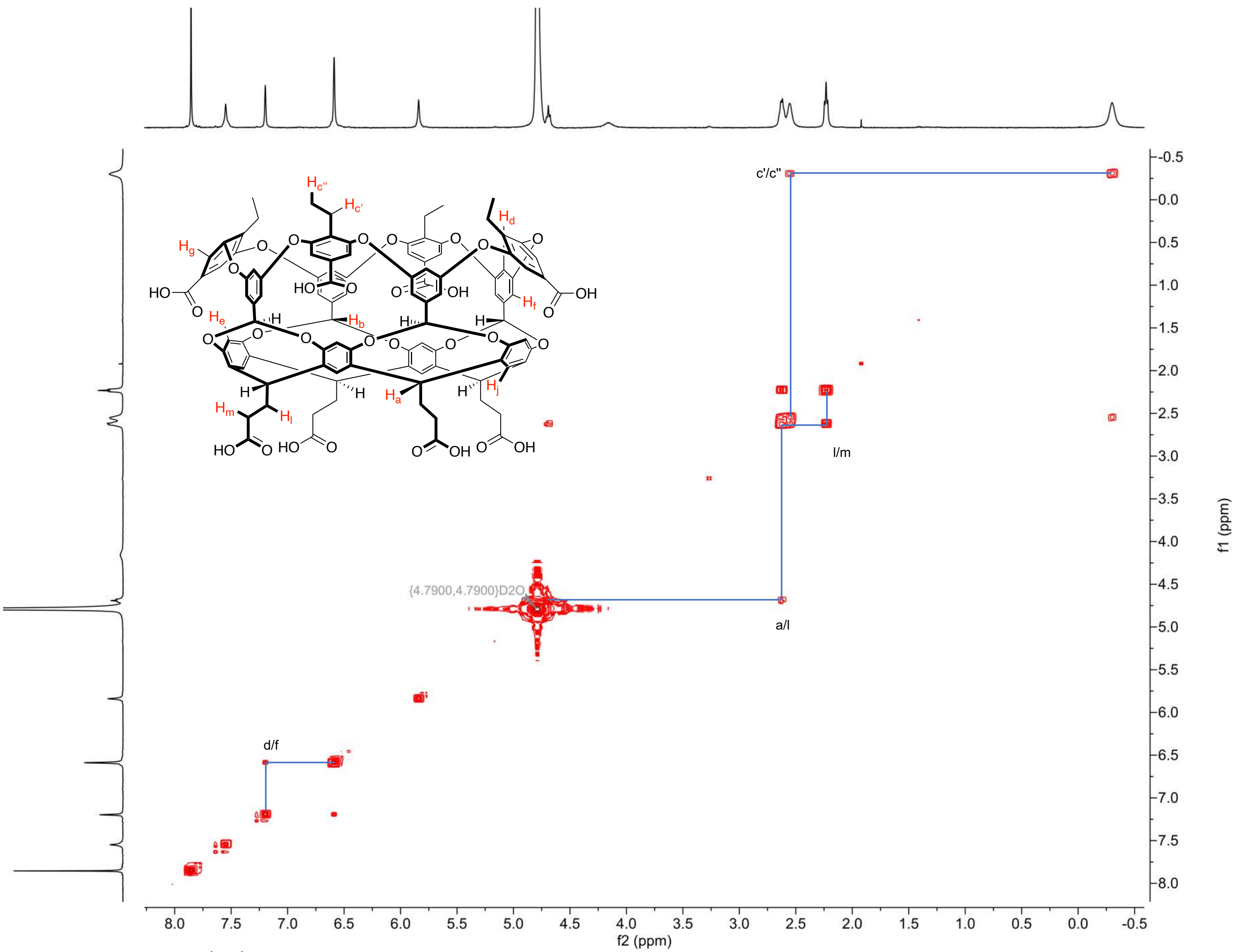

Figure S22: ^1H–^1H COSY NMR spectrum of 1 mM tetra-*endo*-ethyl octa acid, **2**, in 10 mM phosphate buffered D_2O, pD 11.45.

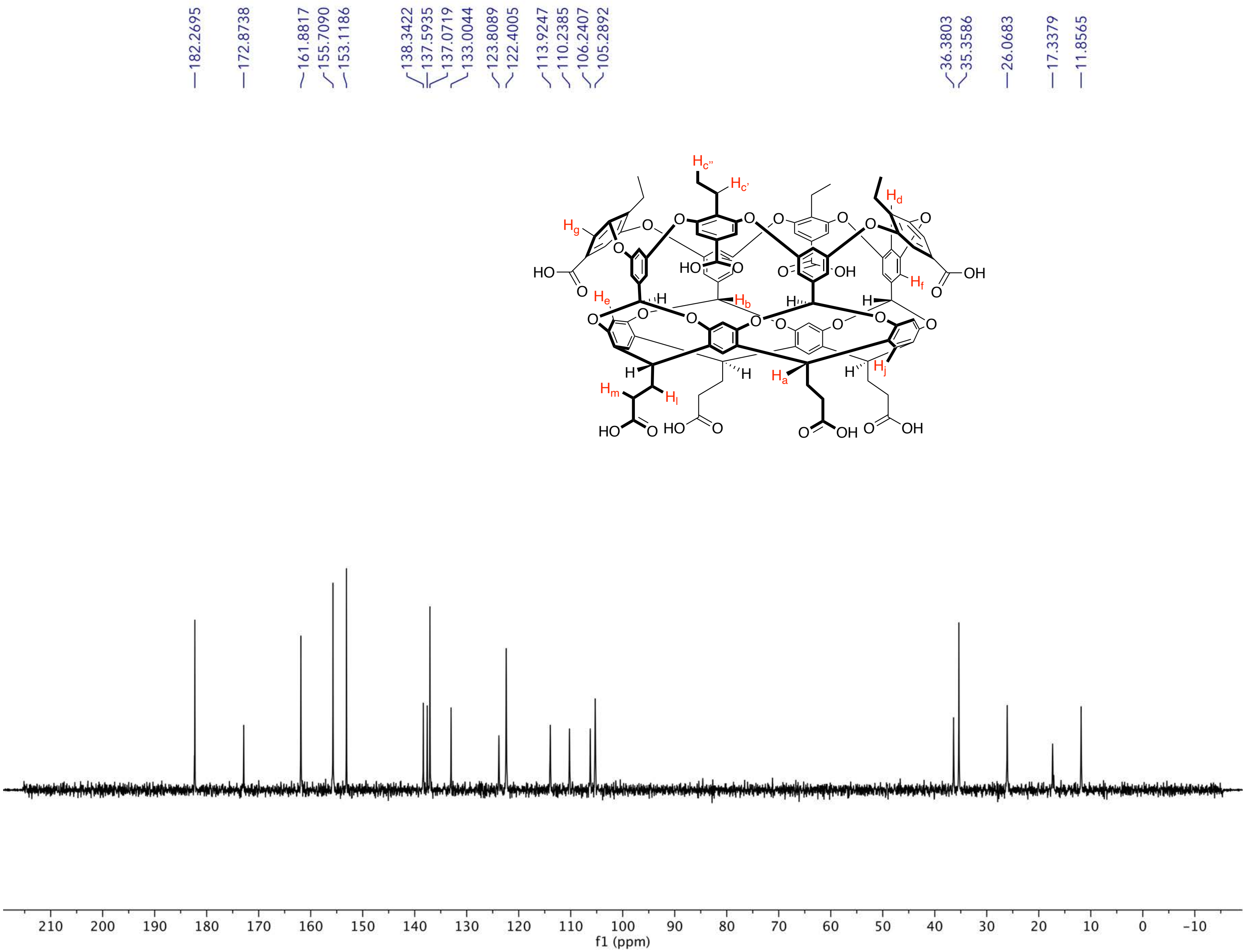

Figure S23: $^{13}C\{^1H\}$ NMR spectrum of 10 mM tetra-*endo*-ethyl octa acid, **2**, in phosphate buffered D_2O, pD 11.45.

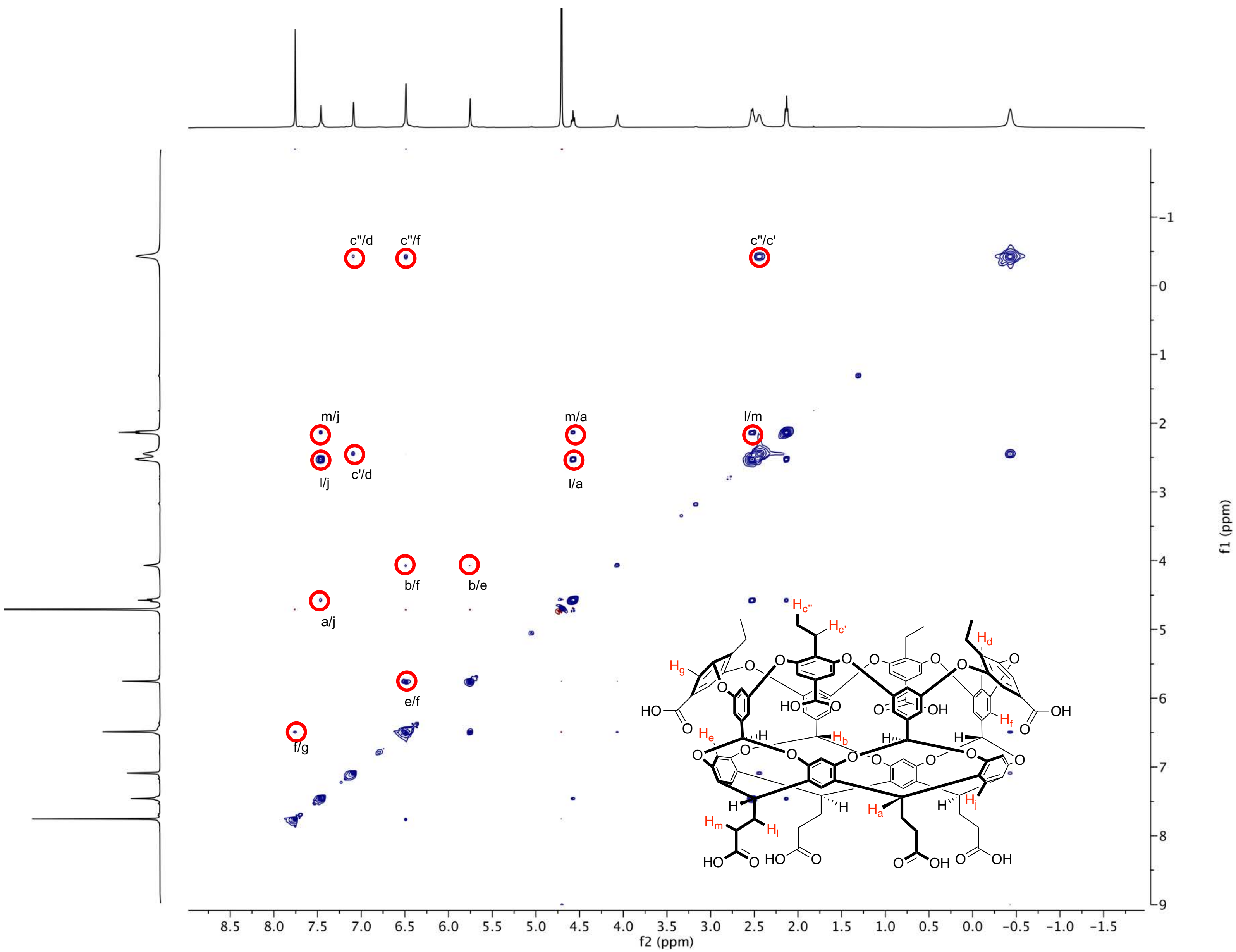

Figure S24: 2D ^{1}H–^{1}H NOESY spectrum of 10 mM tetra-*endo*-ethyl octa acid, **2**, in 10 mM phosphate buffered D_2O, pD 11.45.

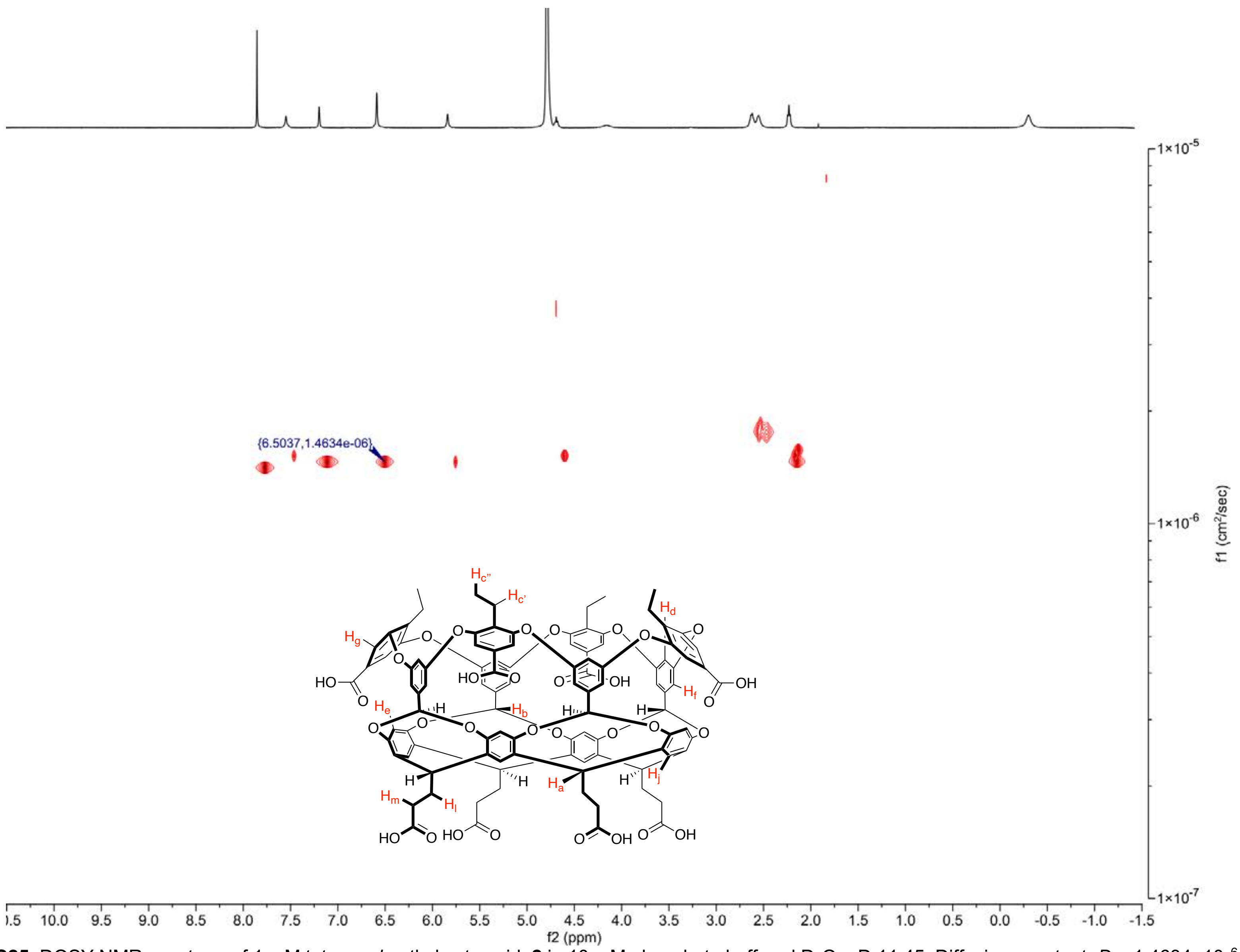

Figure S25: DOSY NMR spectrum of 1 mM tetra-*endo*-ethyl octa acid, **2** in 10 mM phosphate buffered D_2O, pD 11.45. Diffusion constant, $D = 1.4634 \times 10^{-6}$ cm^2 s^{-1}

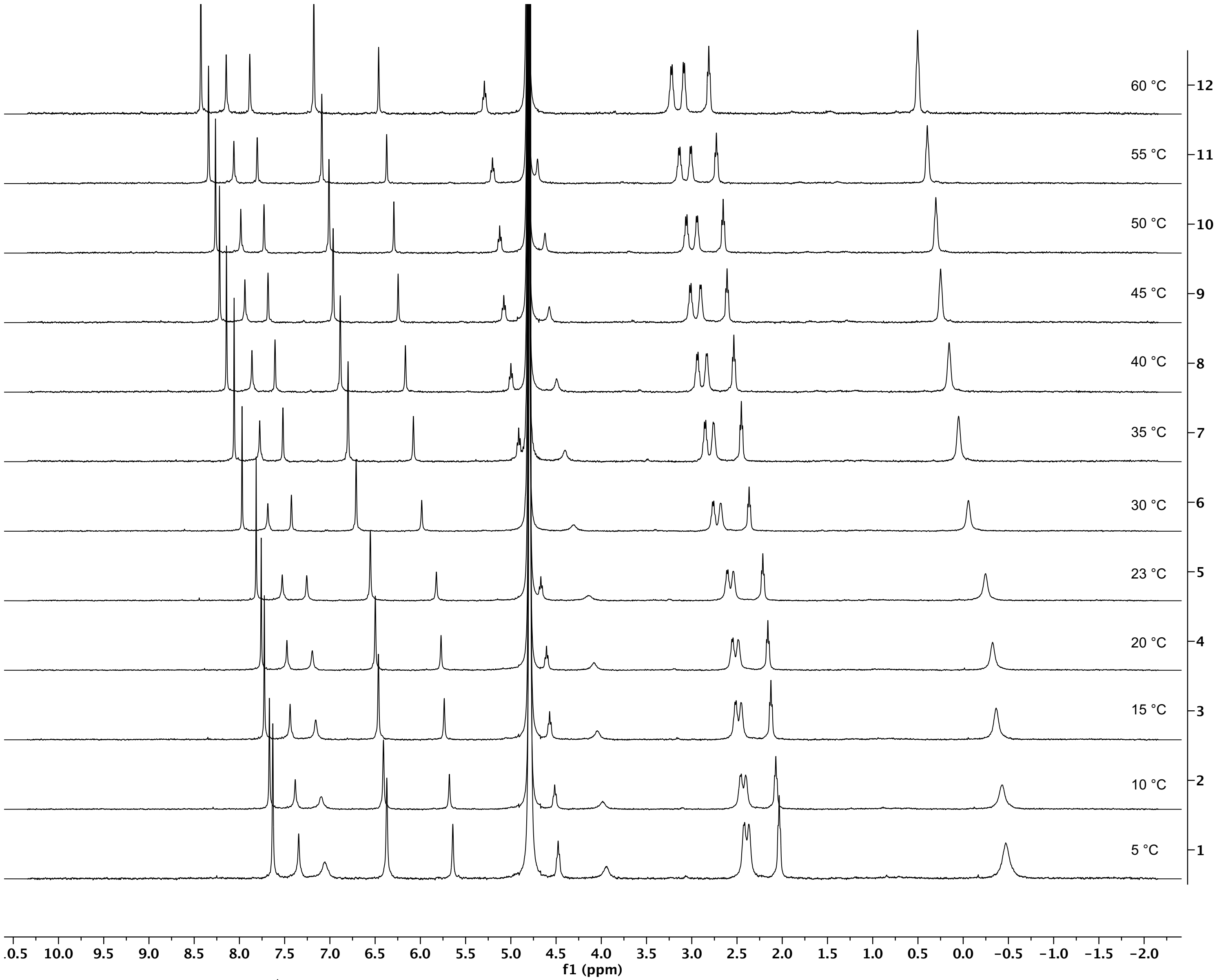

Figure S26: Variable-temperature ^{1}H NMR spectra of 1 mM tetra-*endo*-ethyl octa acid, **2**, in 10 mM phosphate buffered D_2O, pD 11.45. Temperatures are annotated on each spectrum.

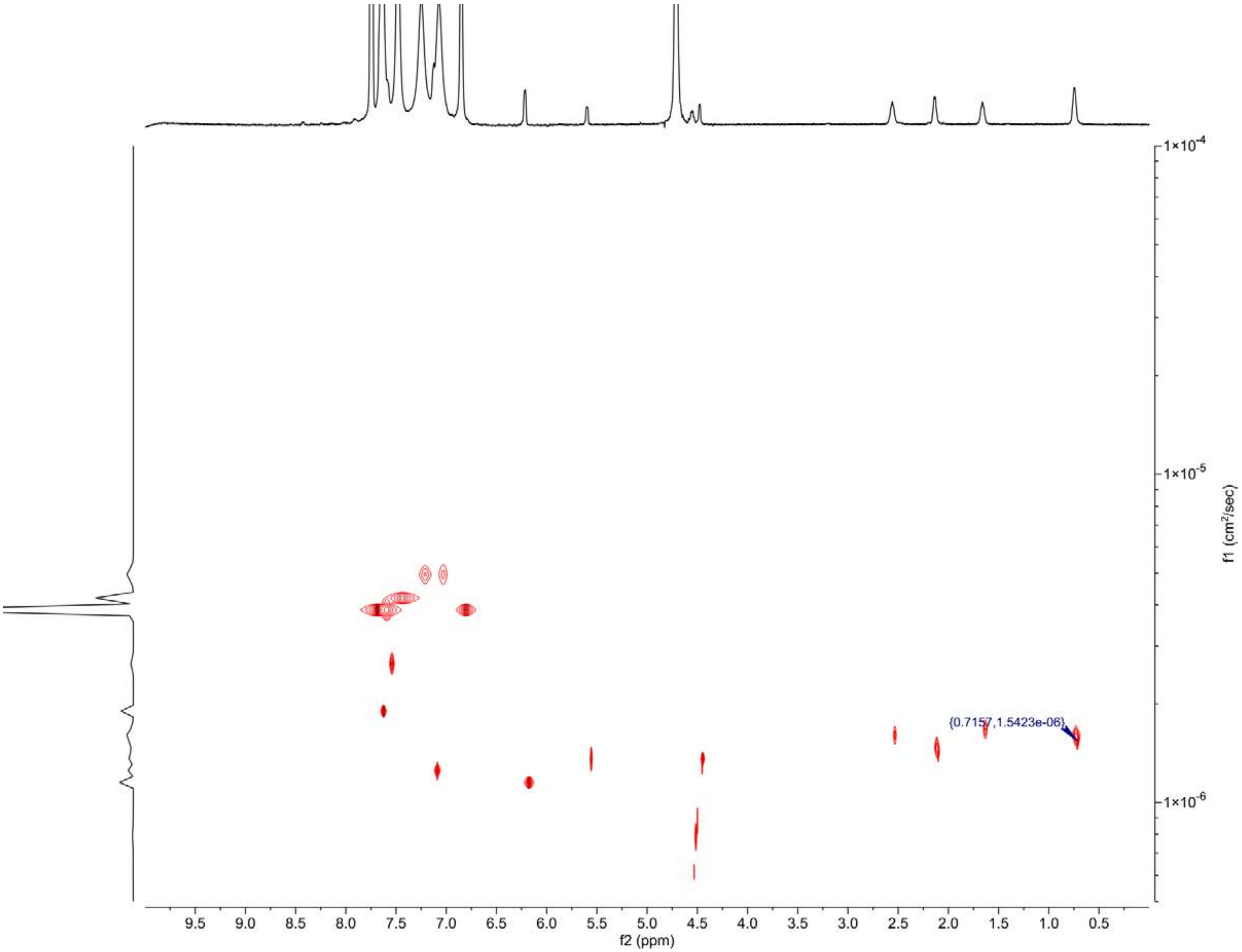

Figure S27: DOSY NMR spectrum of 1mM tetra-*endo*-ethyl octa acid, **2**, complex with 100 equiv. **G1**, in 10 mM phosphate buffered D_2O, pD 11.45. Diffusion constant, $D = 1.5423\times10^{-6}$ $cm^2\ s^{-1}$.

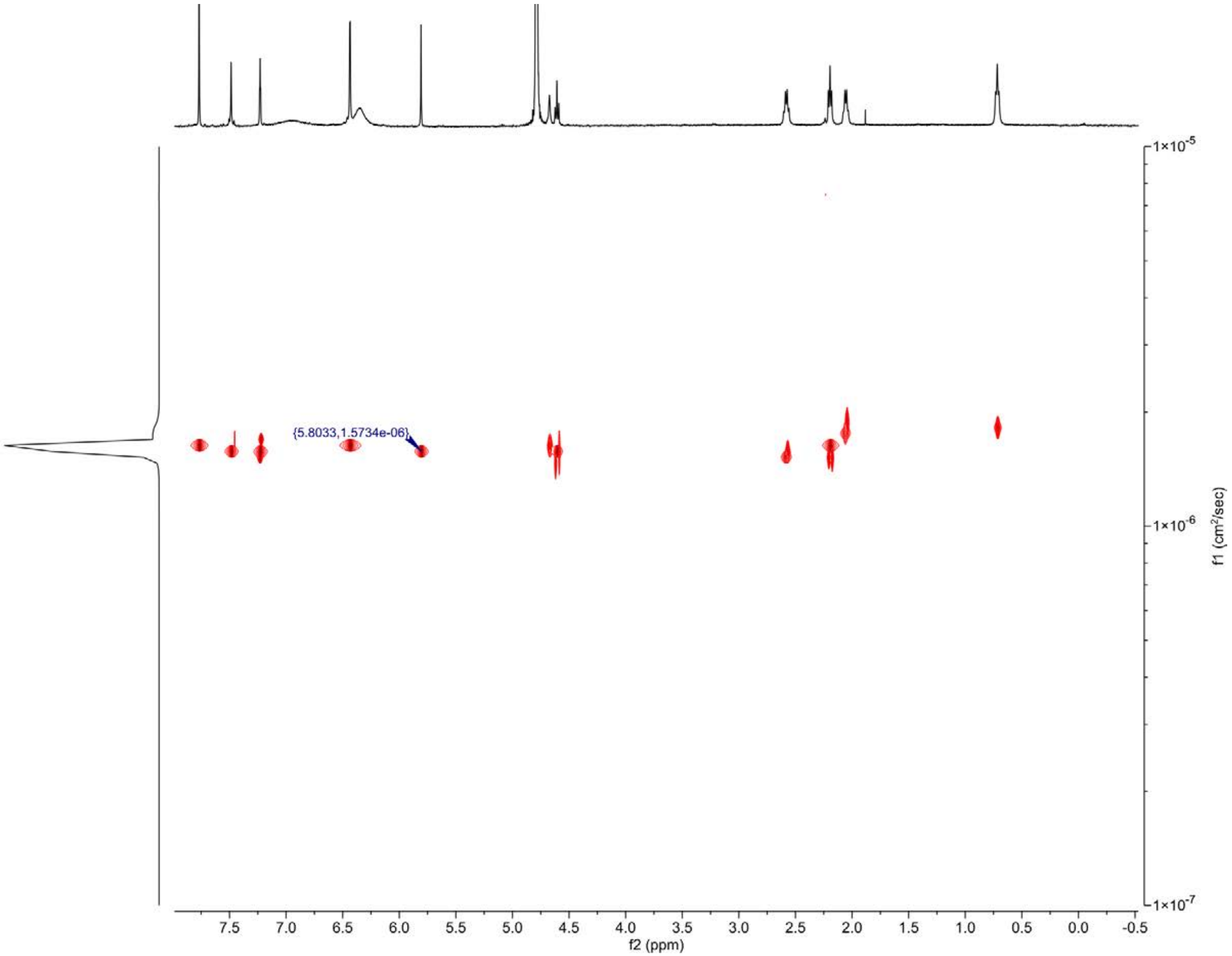

Figure S28: DOSY NMR spectrum of 1 mM tetra-*endo*-ethyl octa acid, **2**, complex with 50 equiv. **G2**, in 10 mM phosphate buffered D_2O, pD 11.45. Diffusion constant, $D = 1.5734 \times 10^{-6}$ cm^2 s^{-1}.

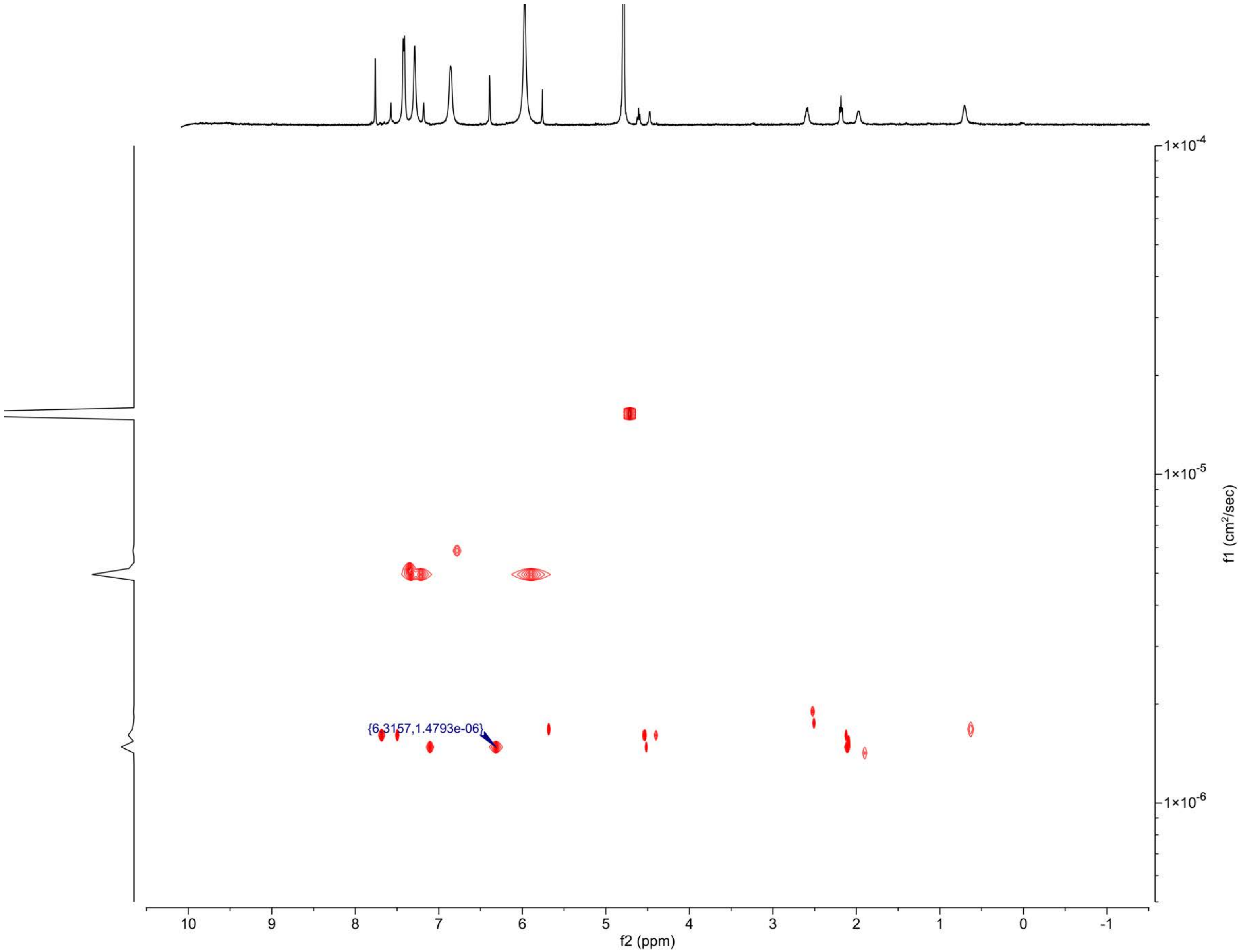

Figure S29: DOSY NMR spectrum of 1 mM tetra-*endo*-ethyl octa acid, **2**, complex with 100 equiv. **G4**, in 10 mM phosphate buffered D_2O, pD 11.45. Diffusion constant, $D = 1.4793 \times 10^{-6}$ cm^2 s^{-1}.

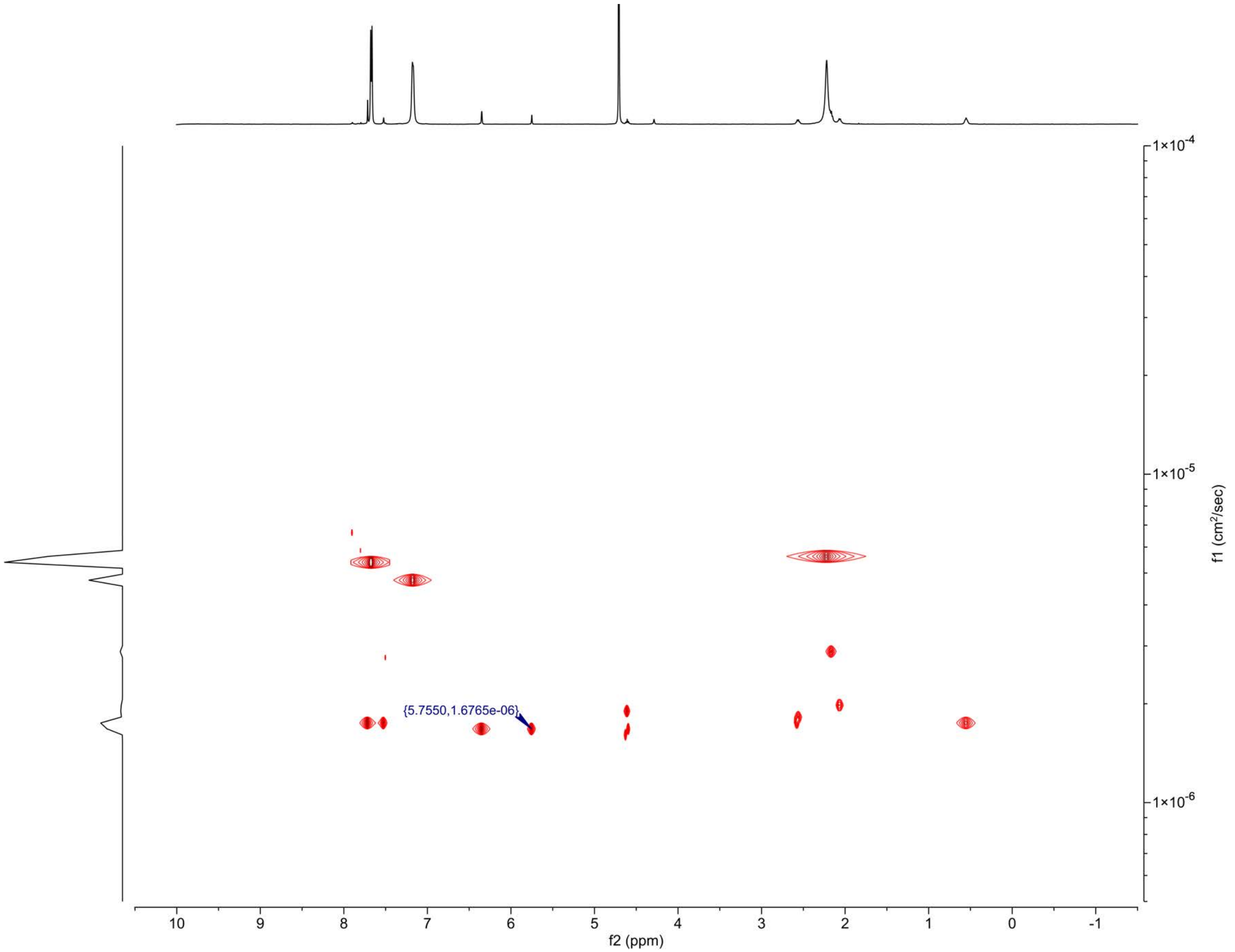

Figure S30: DOSY NMR spectrum of 1 mM tetra-*endo*-ethyl octa acid, **2**, complex with 100 equiv. **G5**, in 10 mM phosphate buffered D_2O, pD 11.45. Diffusion constant, $D = 1.6765\times10^{-6}$ cm^2 s^{-1}.

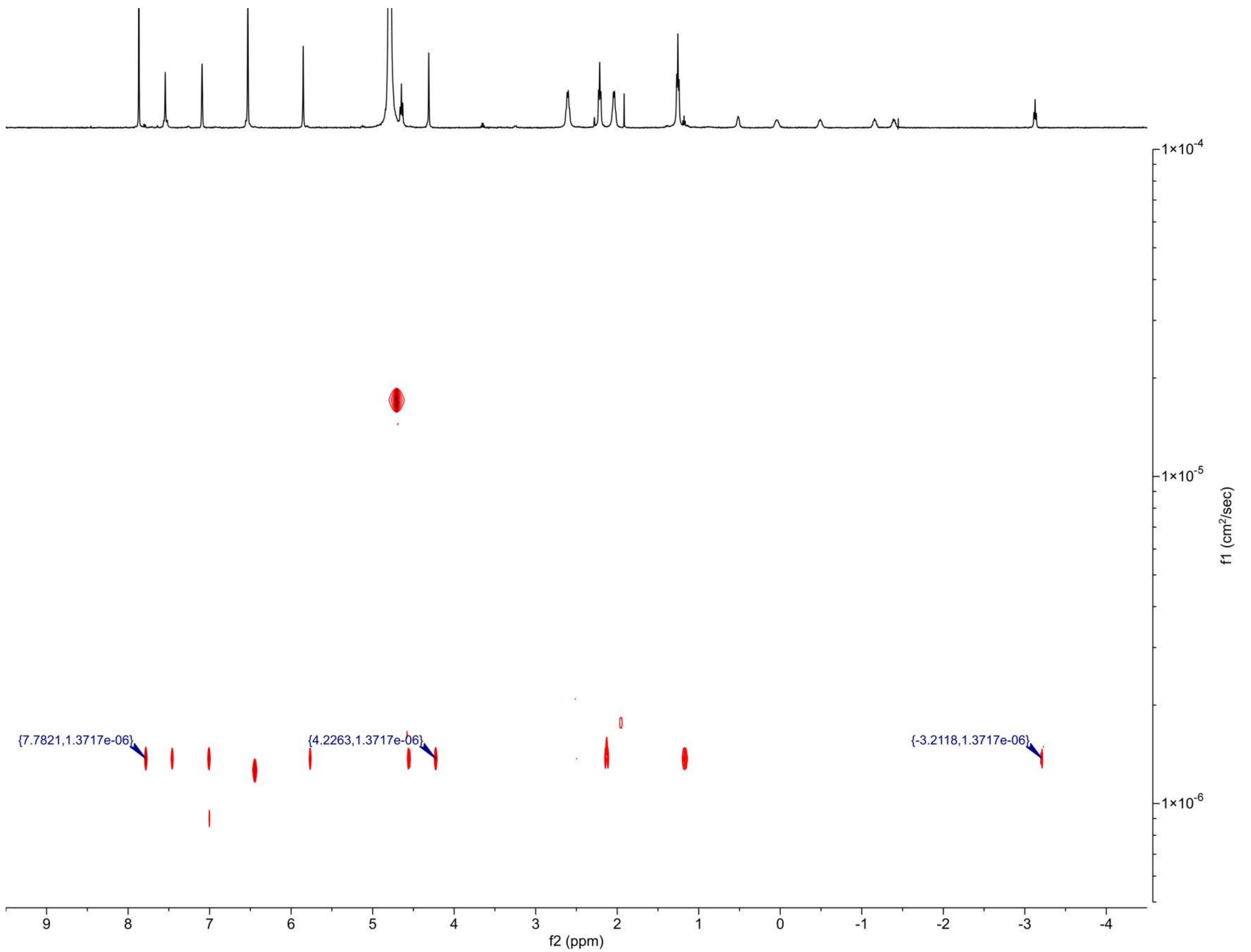

Figure S31: DOSY NMR spectrum of 1.0 mM tetra-endo-ethyl octa acid, **2**, dimerised with *n*-$C_{12}H_{26}$, in 10 mM phosphate buffered D_2O, pD 11.45. Diffusion constant, $D = 1.3717\times10^{-6}$ $cm^2\ s^{-1}$

C. Summary of thermodynamic data

Table S1: Thermodynamic data[a] from ITC and/or NMR for the binding of guests **G1**–**G5** with hosts TEMOA **1** and TEEtOA **2**. All titrations were performed in 10 mM phosphate buffer at pH 11.5.

**Guest**	**TEMOA 1**				**TEEtOA 2**			
	**$\log_{10} K_a$**	**ΔG (kJ mol^{-1})**	**ΔH (kJ mol^{-1})**	**$-T\Delta S$ (kJ mol^{-1})**	**$\log_{10} K_a$**	**ΔG (kJ mol^{-1})**	**ΔH (kJ mol^{-1})**	**$-T\Delta S$ (kJ mol^{-1})**
**G1**	5.10 ± 0.04	–29.1 ± 0.2	–71.2 ± 5.3	42.1 ± 5.1	3.30 ± 0.03	–18.8 ± 0.2	–57.1 ± 0.7	38.3 ± 0.6
**G2**	6.17 ± 0.02	–35.2 ± 0.1	–65.6 ± 1.0	30.3 ± 1.0	3.77 ± 0.02	–21.6 ± 0.1	–48.7 ± 1.2	27.2 ± 1.1
**G3**	4.24 ± 0.02	–24.2 ± 0.1	–33.2 ± 1.0	09.0 ± 0.8	—[b]	—[b]	—[b]	—[b]
**G4**	5.66 ± 0.02	–32.3 ± 0.1	–74.1 ± 1.4	41.8 ± 1.3	3.27 ± 0.03	–18.7 ± 0.2	–54.3 ± 3.6	35.6 ± 3.4
**G5**	4.89 ± 0.01	–27.9 ± 0.1	–59.6 ± 3.2	31.7 ± 3.1	2.43 ± 0.02[c]	–13.9 ± 0.1[c]	—	—

[a] The ΔH and K_a values were obtained by carrying out at least three separate experiments, averaging each set of data, and calculating the respective standard deviations. ΔG was obtained from K_a via the standard thermodynamic equation. The average ΔH and ΔG values were then used to calculate an average $-T\Delta S$, and the corresponding standard deviations calculated using the standard equation for the propagation of uncertainties for subtraction. The deviations in $\log_{10} K_a$ and ΔG were obtained by using the standard equation for the propagation of uncertainties for logarithms.

[b] Binding is too weak to be observed by NMR or ITC.

[c] Determined by ^{1}H NMR spectroscopy.

D. Isothermal titration calorimetry (ITC): instrumentation

Isothermal Titration Calorimetric (ITC) experiments within this study were performed at 298 K using a VP-ITC MicroCalorimeter from Microcal, USA. Integrated heat data obtained for the titrations were fitted using the MicroCal-Origin 7.0 software package. All titrations were carried out in 10 mM sodium phosphate buffer of pH ~11.5 ± 0.05 at 25 °C. Before each experiment, both host and guest solutions were degassed for 2–5 min to eliminate air bubbles. The injection volumes for all titrations used the computer-controlled injection procedure of guest solution into host solution. The injection volumes used for each titration are detailed in the next section.

The binding for most host-guest pairs gave adequate heats of injection such that general ITC titration procedures could be followed. However, in the case of titrations to **2**, the Wiseman "*c*" values (c = [host] × K_a) were less than ideal (i.e., < 5),[6] and modification procedures defined by Turnbull[7] and Tellinghuisen[8] were followed. Thus, for host **2**, large excesses of guest were injected into the host solution with the *N* parameter fixed to 1.0. We have used the same modification procedures previously.[9-11] Moreover, because the heats of complexation were relatively low, higher concentrations of the guest titrant were required which necessitated guest dilution reference titrations (guest injected into buffer solution without host) to be carried out and subtracted from the host-guest titration.

All the ITC titrations to TEMOA **1** exhibited clear thermal responses and gave an excellent fit for a 1:1 complex model. Data from ITC titrations to TEEtOA **2** did not converge to a 1:1 model, and thus *N* was set to 1.00. NMR spectroscopic data are provided to corroborate 1:1 binding. All titrations were run in triplicate, and good reproducibility of K_a and ΔH values with the experimental error between runs less than 5%, and $-T\Delta S$ values less than 10% (Table S1).

E. ITC and NMR experimental parameters

Hosts **1** and **2** exist as hydrates in the solid state and as such the true molar mass $\boldsymbol{M_x}$, which accounts for waters of hydration, is required for accurate determination of binding constants either by ITC or ^{1}H NMR spectroscopy. Thus, the general method for the determination of the true molar mass of **1** and **2** is as follows. Three 2.0 mL 0.5 mM solutions of host were prepared analytically in 10 mM NaOD assuming that the host was anhyd. (M^1_{anh} = 1785.64 g mol^{-1}; M^2_{anh} = 1841.75 g mol^{-1}). Three stocks of 100 mM $EtSO_3Na$ in D_2O were made and were used as an internal analytical standard. Nine (9) different combinations composed of 3 host stocks and 3 standard stocks were combined in 9 NMR tubes (500 µL host stock + 5 µL standard stock). An NMR spectrum of each tube was then taken using a standard 16 scan ^{1}H NMR pulse program with a spectral window spanning δ –3 – 10, and a delay time between excitation and observation pulses of 30 s. The methyl triplet of $EtSO_3^-$ was then integrated and set to a standard integral of 3.00, while the integral for the host H_m peak (see **Fig. S14**) was allowed to float. The integrals of H_m were then averaged and used in the below formula and the resulting average molar mass $\langle \boldsymbol{M_x} \rangle$ was obtained:

$$\langle M_x \rangle = \frac{W_x \cdot V_x \cdot I_{cal} \cdot N_x}{V_{Sx} \cdot (V_x + V_{cal}) \cdot \langle I_x \rangle \cdot N_{cal} \cdot C_{cal}} \cdot 10^6$$

where

$\boldsymbol{W_x}$ is the gravimetric mass of the host in the stock solution (g);
$\boldsymbol{V_{Sx}}$ is the volume of the host stock solution (mL);
$\langle \boldsymbol{I_x} \rangle$ and $\boldsymbol{I_{cal}}$ are the averaged integrals of the H_m, and of the $EtSO_3^-$ methyl signals, respectively;
$\boldsymbol{N_x}$ and $\boldsymbol{N_{cal}}$ are the number of nuclei in the H_m and $EtSO_3^-$ methyl signals, respectively;
$\boldsymbol{C_{cal}}$ is the the final concentration of $EtSO_3Na$ in the NMR tube (mM);
$\boldsymbol{V_x}$ and $\boldsymbol{V_{cal}}$ are the volumes of host and $EtSO_3Na$ solutions added to the NMR tube, respectively (µL).

All solutions (including stock buffer solutions) for ITC were prepared in volumetric flasks which have been previously washed once with freshly prepared Caro's acid (1:5 30% aq. H_2O_2/98% H_2SO_4), and thrice with ultra-pure MilliQ water. Stock solutions of phosphate buffer (10 mM, pH 11.5) were prepared by adding $Na_2HPO_4 \cdot 7H_2O$ (1.93 g, 7.2 mmol), Na_3PO_4 (0.44 g, 2.7 mmol), and ultra-pure MilliQ water to a 1 L volumetric flask. Appropriate volumes of host and guest solutions were prepared in the normal fashion by dissolving the appropriate mass of host or guest in a volumetric flask using the above prepared stock buffer solution. The pH of the solution was measured and adjusted to pH 11.5 ± 0.05 by titrating in 5 M NaOH or 5 M HCl as appropriate. All ITC titrations utilised ultra-pure MilliQ water in the reference cell.

All solutions (including stock buffer solutions) for ^{1}H NMR spectroscopic titrations were prepared in volumetric flasks which have been previously washed once with freshly prepared Caro's acid (1:5 30% aq. H_2O_2/98% H_2SO_4), and thrice with 99.9% D_2O. Stock solutions of phosphate buffer (10 mM, pH 11.9, pD 11.5) were prepared by adding $Na_2HPO_4 \cdot 7H_2O$ (0.134 g, 0.5 mmol), Na_3PO_4 (0.066 g, 0.4 mmol), and 99% D_2O in a 100 mL volumetric flask. Appropriate volumes of host and guest solutions were prepared in the normal fashion by dissolving the appropriate mass of host or guest in a volumetric flask using the above prepared stock buffer solution. The pH of the solution was measured and adjusted to pH 11.9 ± 0.05 (pD 11.5 ± 0.05) by titrating in 5 M NaOD or 5 M DCl as appropriate. All ^{1}H NMR titrations were performed on a

Bruker 500.13 MHz instrument operating at 25 °C, using residual H_2O (δ 4.79) as an internal standard.

Diffusion-Ordered Spectroscopy NMR (DOSY-NMR) data were acquired using a Bruker Avance 500 MHz Instrument operating at 25 °C using 10 mM phosphate buffered D_2O at pD 11.5. Using Topspin 1.2, the LEDBGPG2S (2D sequence for diffusion measurement using stimulated echo and LED using bipolar gradient pulses for diffusion using 2 spoil gradients) was employed with an exponential ramp of the gradient strength from 2–95%, and using the following parameters:

p1 = 7.250 μs
p2 = 14.500 μs
p19 = 600.000 μs
δ = 0.004 s
Δ = 0.200 s
d1 = 2.00 s
d16 = 0.1 ms
ns = 32
TD1 = 128

DOSY NMR processing was performed using MNova 14 using a *k* factor of 0.996, and utilising the Peak Fit method with a single decay component and 64 points in the diffusion dimension.

Each host-guest system required specific conditions to fit within the instrumental limitations inherent of ITC. Specifically, the concentrations for host and guest, injection procedure, and DP (Differential Power) value used for each host-guest pair is listed below.

3-Hydroxy-2-naphthoic acid (G1):
TEMOA **1**: DP = 25 μcal s^{-1}. The ITC titration experiment utilized a 35-injection procedure of 1.5 mM guest solution titrated into 0.15 mM host solution. V_1 = 3.0 μL; $V_2 – V_7$ = 5.0 μL; $V_8 – V_{12}$ = 7 μL; $V_{13} – V_{35}$ = 9 μL. $[H]_{final}$ = 0.0968 mM, $[G]_{final}$ = 0.532 mM.

TEEtOA **2**: DP = 15 μcal s^{-1}. The ITC titration experiment utilized a 28-injection procedure of 20 mM guest solution titrated into 0.10 mM host solution. V_1 = 2.0 μL; $V_2 – V_{28}$ = 9.0 μL. $[H]_{final}$ = 0.0101 mM, $[G]_{final}$ = 0.0395 mM.

***p*-Bromophenol (G2):**
TEMOA **1**: DP = 25 μcal s^{-1}. The ITC titration experiment utilized a 35-injection procedure of 1.5 mM guest solution titrated into 0.15 mM host solution. V_1 = 3.0 μL; $V_2 – V_7$ = 5.0 μL; $V_8 – V_{12}$ = 7 μL; $V_{13} – V_{35}$ = 9 μL. $[H]_{final}$ = 0.0968 mM, $[G]_{final}$ = 0.532 mM.

TEEtOA **2**: DP = 15 μcal s^{-1}. The ITC titration experiment utilized a 28-injection procedure of 7.5 mM guest solution titrated into 0.10 mM host solution. V_1 = 2.0 μL; $V_2 – V_{28}$ = 9.0 μL. $[H]_{final}$ = 0.0671 mM, $[G]_{final}$ = 2.47 mM.

Cyclopentylacetic acid (G3):
TEMOA **1**: DP = 25 μcal s^{-1}. The ITC titration experiment utilized a 35-injection procedure of 15 mM guest solution titrated into 0.15 mM host solution. V_1 = 3.0 μL; $V_2 – V_7$ = 5.0 μL; $V_8 – V_{12}$ = 7 μL; $V_{13} – V_{35}$ = 9 μL. $[H]_{final}$ = 0.0968 mM, $[G]_{final}$ = 5.32 mM.

Piperonylic acid (G4):
TEMOA **1**: DP = 25 μcal s^{-1}. The ITC titration experiment utilized a 35-injection procedure of 1.5 mM guest solution titrated into 0.15 mM host solution. V_1 = 3.0 μL; V_2 – V_7 = 5.0 μL; V_8 – V_{12} = 7 μL; V_{13} – V_{35} = 9 μL. $[H]_{final}$ = 0.0968 mM, $[G]_{final}$ = 0.532 mM.

TEEtOA **2**: DP = 15 μcal s^{-1}. The ITC titration experiment utilized a 28-injection procedure of 20 mM guest solution titrated into 0.10 mM host solution. V_1 = 2.0 μL; V_2 – V_{28} = 9.0 μL. $[H]_{final}$ = 0.0671 mM, $[G]_{final}$ = 6.58 mM.

***p*-Toluic acid (G5):**
TEMOA **1**: DP = 25 μcal s^{-1}. The ITC titration experiment utilized a 35-injection procedure of 1.2 mM guest solution titrated into 0.15 mM host solution. V_1 = 3.0 μL; V_2 – V_7 = 5.0 μL; V_8 – V_{12} = 7 μL; V_{13} – V_{35} = 9 μL. $[H]_{final}$ = 0.0968 mM, $[G]_{final}$ = 0.426 mM.

Variable Temperature (VT) NMR Data

The in–out dynamics of the pendent ethyl groups in **2** were probed using VT ^{1}H NMR spectroscopy. The experiment was performed on a 1 mM solution of **2** in 10 mM phosphate-buffered D_2O at pD 11.5 prepared as described above, using a Bruker Avance 500 NMR spectrometer whose probe temperature was calibrated against the Δδ difference between the residual CD_2HOH signals in 99.8% CD_3OD (–10 – 30°C) or the CH_2–OH signals in 80% ethylene glycol in DMSO-*d*$_6$ (30–60 °C).

Table S2: Chemical shift changes as a function of temperature for 2 from 30–60 °C

Temperature (°C)	⟨Δδ⟩ ± 1σ [a] (ppm)	Δδ H_d	Δδ H_b	Δδ $H_{c'}$	Δδ $H_{c''}$
30	0	0	0	0	0
35	0.0938 ± 0.0122	0.0924	0.0941	0.0778	0.1079
40	0.1791 ± 0.0126	0.1799	0.1863	0.1533	0.1738
45	0.2568 ± 0.0134	0.2578	0.2695	0.2236	0.3088
50	0.3022 ± 0.0140	0.3019	0.3169	0.2625	0.3608
55	0.3798 ± 0.0149	0.3777	0.3982	0.3305	0.4552
60	0.4666 ± 0.0171	0.4593	—[b]	0.4042	0.5610

[a] The ⟨Δδ⟩ was calculated for protons distal to the protons of interest, i.e., H_g, H_j, H_f, H_e, H_a, H_l, and H_m, with the zero point at 30 °C. The errors denote one standard deviation.
[b] Signal was underneath the H_2O peak and is thus unresolved.

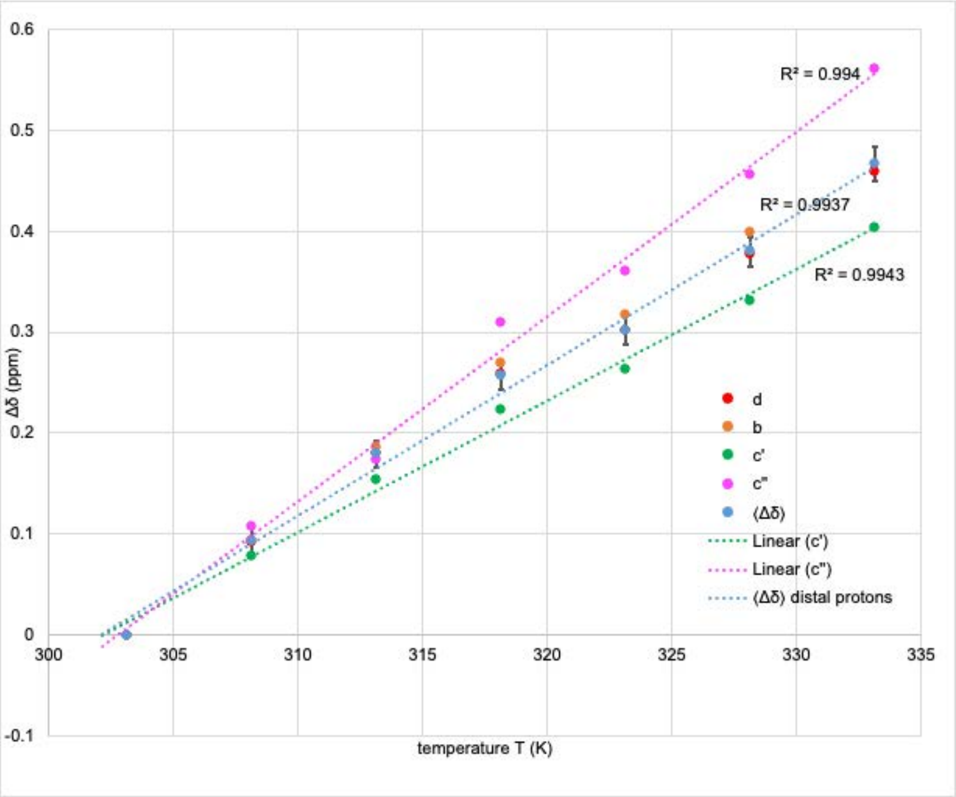

Figure S32: Plot of Δδ as a function of temperature from 30–60 °C. The blue line indicates the average of the Δδ for distal protons (H_j, H_g, H_f, H_e, H_a, H_l, H_m) ; the green and pink lines show the deviation of the $H_{c'}$ and $H_{c''}$ with increasing temperature.

F. ITC and NMR results

The figures below show an example thermogram and binding curve of one titration experiment for each host–guest pair.

3-Hydroxy-2-naphthoic acid (G1)

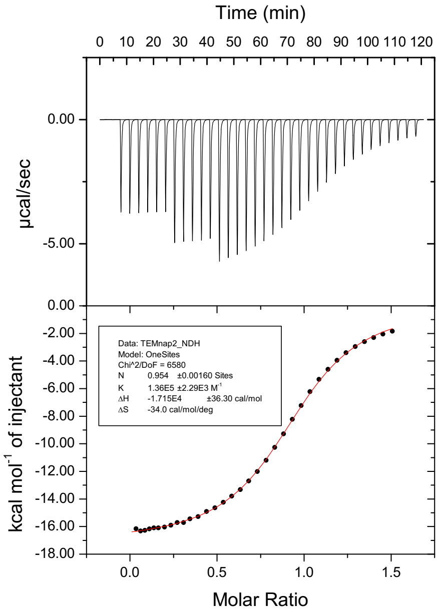

Figure S33: ITC thermogram and 1:1 binding fit for TEMOA–G1 complexation. A 1.5 mM solution of G1 was titrated into a 0.15 mM solution of TEMOA equilibrated at 25 °C. Both host and guest were in 10 mM phosphate buffer, pH 11.5.

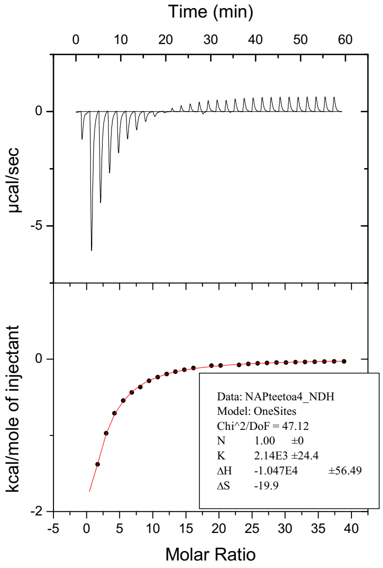

Figure S 34: ITC thermogram and 1:1 binding fit for TEEtOA–G1 complexation. A 20.0 mM solution of G1 was titrated into a 0.10 mM solution of TEEtOA equilibrated at 25 °C. Both host and guest were in 10 mM phosphate buffer, pH 11.5

p-Bromophenol (G2)

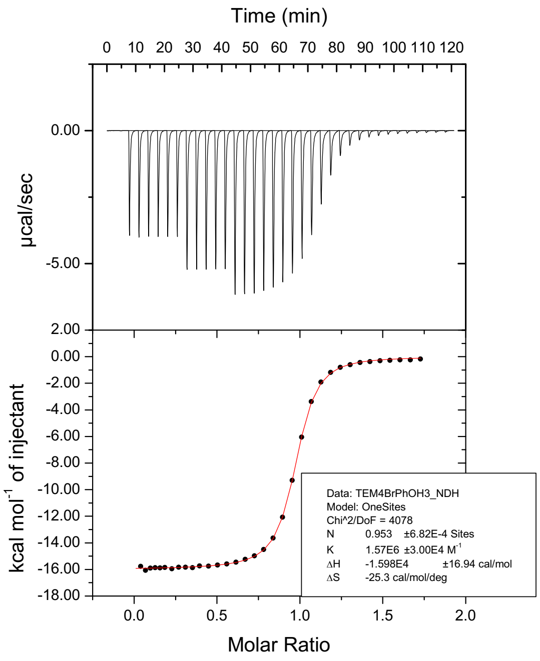

Figure S35: ITC thermogram and 1:1 binding fit for TEMOA–G2 complexation. A 1.5 mM solution of G2 was titrated into a 0.15 mM solution of TEMOA equilibrated at 25 °C. Both host and guest were in 10 mM phosphate buffer, pH 11.5.

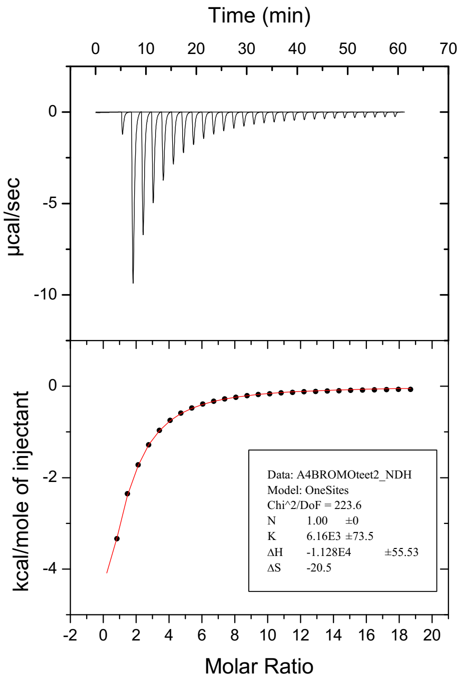

Figure S36: ITC thermogram and 1:1 binding fit for TEEtOA–G2 complexation. A 7.5 mM solution of G2 was titrated into a 0.10 mM solution of TEEtOA equilibrated at 25 °C. Both host and guest were in 10 mM phosphate buffer, pH 11.5.

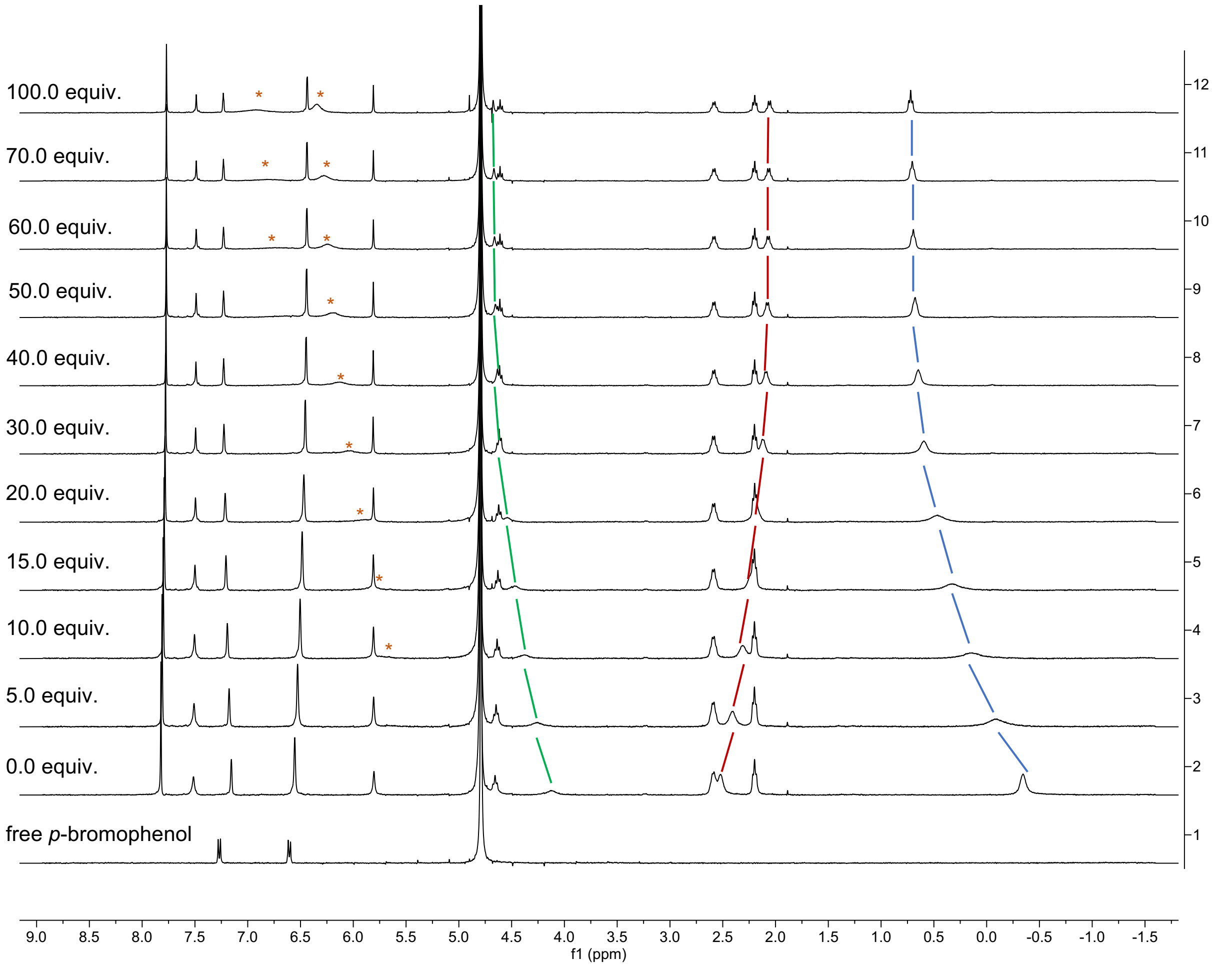

Figure S37: ^{1}H NMR stack showing the addition of aliquots of 500 mM **G2** into a 1.0 mM solution of TEEtOA, **2**. Both solutions were in 10 mM pD 11.4 phosphate-buffered D_2O. Blue, red, and green lines illustrate the shifts in $H_{c''}$, $H_{c'}$, and H_b, respectively, as the titration progresses (See Fig S21 for reference). Orange asterisks indicate emergence and exchange of guest peaks in the bound and free state.

Cyclopentylacetic acid (G3)

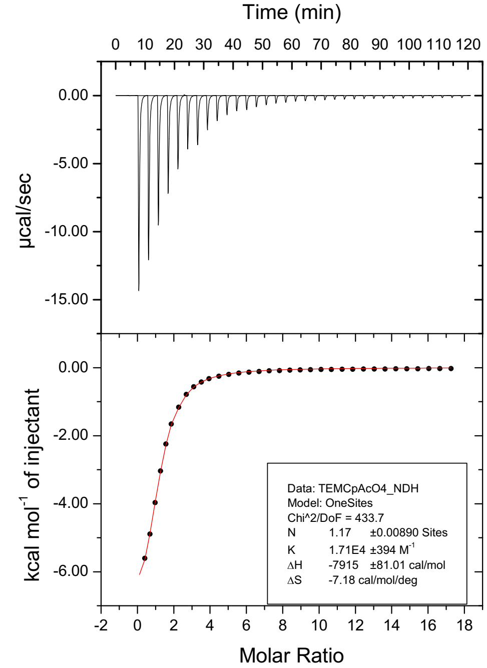

Figure S38: ITC thermogram and 1:1 binding fit for TEMOA–G3 complexation. A 15 mM solution of G3 was titrated into a 0.15 mM solution of TEMOA equilibrated at 25 °C. Both host and guest were in 10 mM phosphate buffer, pH 11.5.

Piperonylic acid (G4)

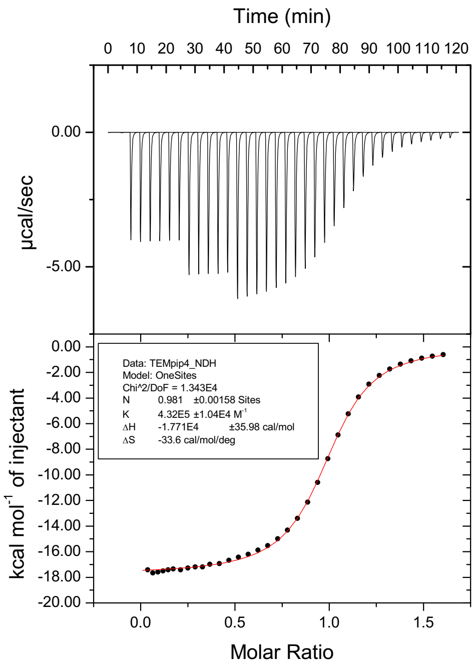

Figure S39: ITC thermogram and 1:1 binding fit for TEMOA–G4 complexation. A 1.5 mM solution of G4 was titrated into a 0.15 mM solution of TEMOA equilibrated at 25 °C. Both host and guest were in 10 mM phosphate buffer, pH 11.5.

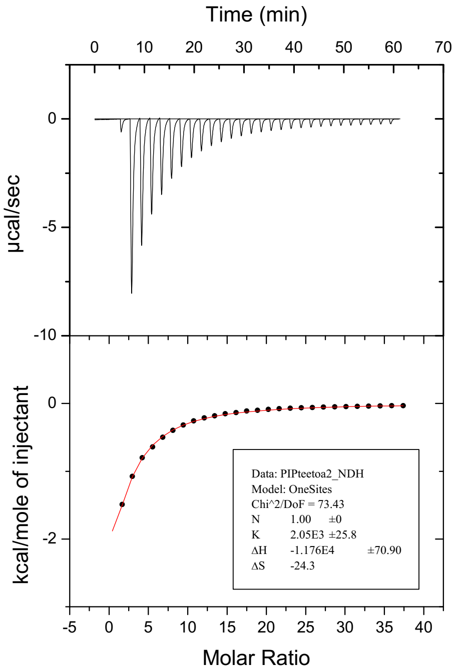

Figure S40: ITC thermogram and 1:1 binding fit for TEEtOA–G4 complexation. A 20.0 mM solution of G4 was titrated into a 0.10 mM solution of TEEtOA equilibrated at 25 °C. Both host and guest were in 10 mM phosphate buffer, pH 11.5.

p-Toluic acid (G5)

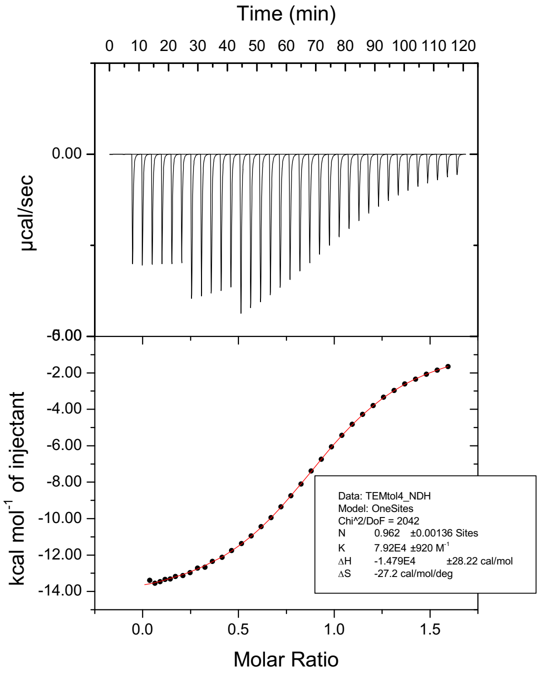

Figure S41: ITC thermogram and 1:1 binding fit for TEMOA–G5 complexation. A 1.2 mM solution of G5 was titrated into a 0.15 mM solution of TEMOA equilibrated at 25 °C. Both host and guest were in 10 mM phosphate buffer, pH 11.5.

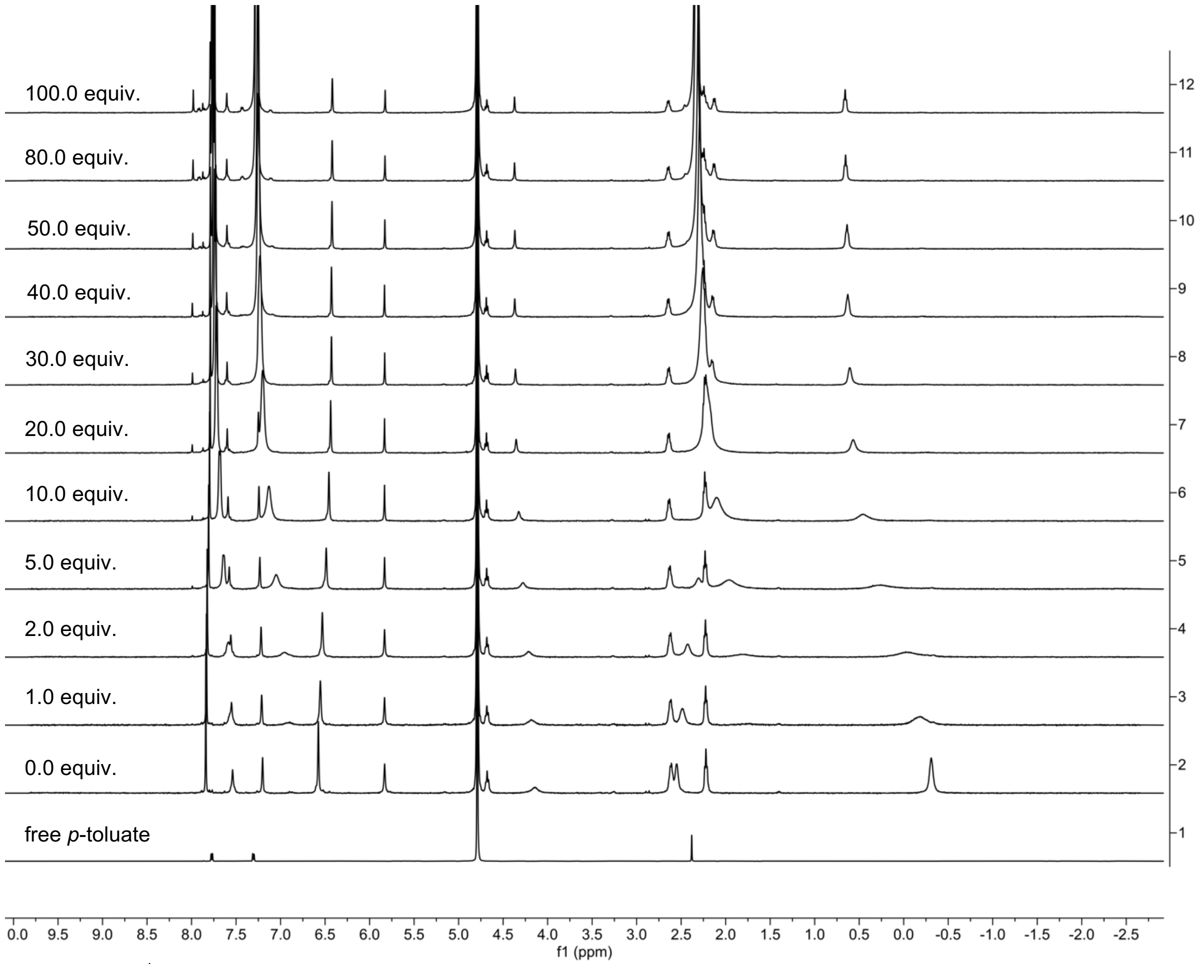

Figure S42: ^{1}H NMR stack showing the addition of aliquots of 500 mM **G5** into a 1.0 mM solution of **TEEtOA**, **2**. Both solutions were in 10 mM pD 11.4 phosphate-buffered D_2O.

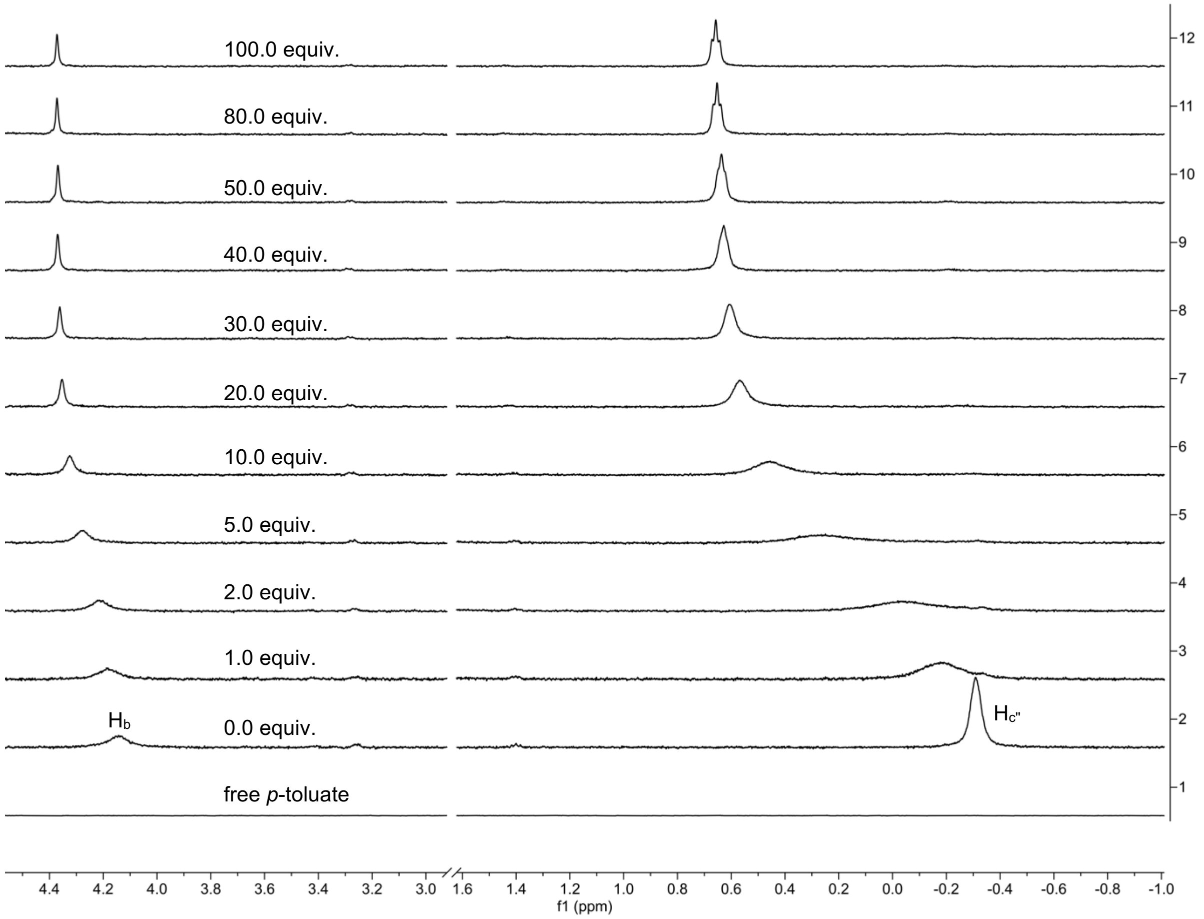

Figure S43: Host region between –1.0 – 1.6 ppm, and 3.0 – 4.6 ppm showing the shifting of **TEEtOA** host peaks H_b and $H_{c''}$ (see Figure S13) as a function of **G5**.

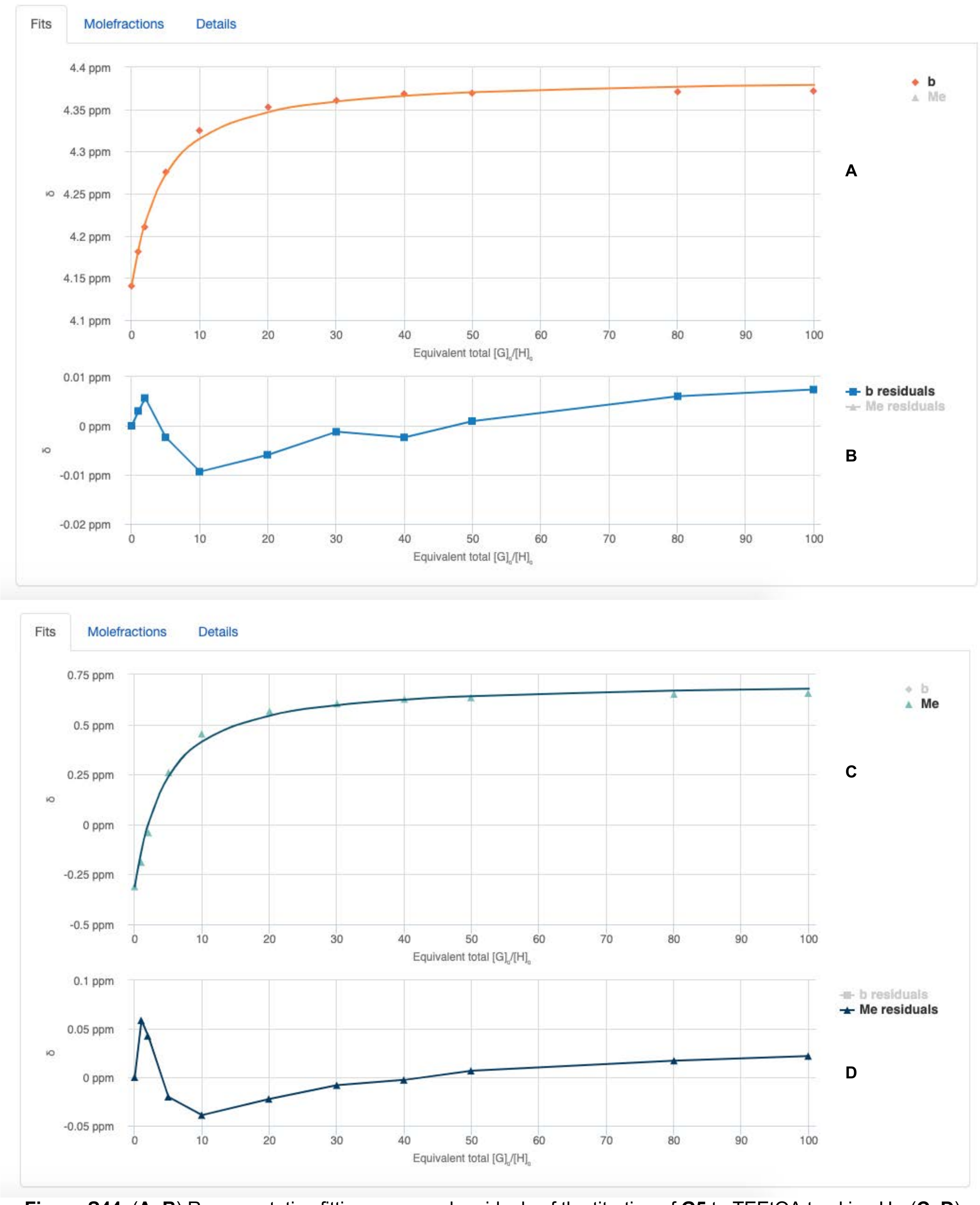

Figure S44: (**A**, **B**) Representative fitting curve and residuals of the titration of **G5** to TEEtOA tracking H_b. (**C**, **D**) Representative fitting curve and residuals of the titration of **G5** to **TEEtOA** tracking $H_{c''}$. Curve and residuals were calculated using the online BindFit software.[12, 13]

G. Crystal structure of 4-ethyl-3,5-dimethoxybenzoic acid, c

A colourless plate-like specimen of 4-ethyl-3,5-dimethoxybenzoic acid **c**, approximate dimensions 0.031 mm × 0.144 mm × 0.236 mm, was used for the X-ray crystallographic analysis. The X-ray intensity data were measured on a Bruker D8 VENTURE PHOTON 100 CMOS system equipped with a INCOATEC IμS micro-focus source (Cu-K_α, λ = 1.54178 Å) and a mirror monochromator.

A total of 1960 frames were collected. The total exposure time was 21.78 hours. The frames were integrated with the Bruker SAINT software package using a narrow-frame algorithm. The integration of the data using a triclinic unit cell yielded a total of 3833 reflections to a maximum θ angle of 71.98° (0.81 Å resolution), of which 1949 were independent (average redundancy 1.967, completeness = 92.2%, R_{int} = 4.23%, R_{sig} = 5.89%) and 1471 (75.47%) were greater than $2\sigma(F^2)$. The final cell constants of a = 4.5802(4) Å, b = 7.9822(7) Å, c = 15.3535(14) Å, α = 79.400(6)°, β = 86.352(6)°, γ = 76.197(6)°, volume = 535.70(8) $Å^3$, are based upon the refinement of the XYZ-centroids of 2508 reflections above 20 σ(I) with 11.59° < 2θ < 144.4°. Data were corrected for absorption effects using the multi-scan method (SADABS). The ratio of minimum to maximum apparent transmission was 0.742. The calculated minimum and maximum transmission coefficients (based on crystal size) are 0.8290 and 0.9750.

The final anisotropic full-matrix least-squares refinement on F^2 with 142 variables converged at R_1 = 6.11%, for the observed data and wR_2 = 14.65% for all data. The goodness-of-fit was 1.052. The largest peak in the final difference electron density synthesis was 0.195 $e^-/Å^3$ and the largest hole was –0.184 $e^-/Å^3$ with an RMS deviation of 0.047 $e^-/Å^3$. On the basis of the final model, the calculated density was 1.303 g cm^{-3} and F(000), 224 e^-.

The weighted R-factor wR and goodness of fit S are based on F^2 conventional R-factors R are based on F, with F set to zero for negative F^2. The threshold expression of $F^2 > 2\sigma(F^2)$ is used only for calculating R-factors(gt) etc. and is not relevant to the choice of reflections for refinement. R-factors based on F^2 are statistically about twice as large as those based on F, and R-factors based on ALL data will be even larger. H-atoms attached to carbon were placed in calculated positions (C–H = 0.95–0.98 Å) and included as riding contributions with isotropic displacement parameters 1.2–1.5 times those of the attached atoms. That attached to oxygen was placed in a position obtained from a difference map and refined with a DFIX 0.87 0.01 instruction. The methyl groups based on C9 and C10 are rotationally disordered and were refined with AFIX 127 instructions. One reflection obscured by the beamstop was omitted from the final refinement.

Table S3: Sample and crystal data for 4-ethyl-3,5-dimethoxybenzoic acid **c**.

**Identification code**	BCG032	
**Chemical formula**	$C_{11}H_{14}O_4$	
**Formula weight**	210.22 g/mol	
**Temperature**	150(2) K	
**Wavelength**	1.54178 Å	
**Crystal size**	0.031 × 0.144 × 0.236 mm	
**Crystal habit**	colourless plate	
**Crystal system**	triclinic	
**Space group**	P –1	
**Unit cell dimensions**	a = 4.5802(4) Å	α = 79.400(6)°
	b = 7.9822(7) Å	β = 86.352(6)°
	c = 15.3535(14) Å	γ = 76.197(6)°
**Volume**	535.70(8) $Å^3$	
**Z**	2	
**Density (calculated)**	1.303 g/cm^3	
**Absorption coefficient**	0.827 mm^{-1}	
**F(000)**	224	

Table S4: Data collection and structure refinement for 4-ethyl-3,5-dimethoxybenzoic acid, **c**.

**Diffractometer**	Bruker D8 VENTURE PHOTON 100 CMOS	
**Radiation source**	INCOATEC IµS micro-focus source (Cu-Kα, λ = 1.54178 Å)	
**2θ range for data collection**	5.80 – 71.98°	
**Index ranges**	–5≤h≤5, –9≤k≤9, –18≤l≤17	
**Reflections collected**	3833	
**Independent reflections**	1949 [R_{int} = 0.0423]	
**Coverage of independent reflections**	92.2%	
**Absorption correction**	multi-scan	
**Max. and min. transmission**	0.9750 and 0.8290	
**Refinement method**	Full-matrix least-squares on F^2	
**Refinement program**	*SHELXL-2018/1*[14]	
**Function minimized**	Σ w(F_o^2 - F_c^2)^2	
**Data / restraints / parameters**	1949 / 1 / 142	
**Goodness-of-fit on F^2**	1.052	
**Final R indices**	1471 data; I>2σ(I)	R_1 = 0.0611, wR_1 = 0.1339
	all data	R_2 = 0.0931, wR_2 = 0.1465
**Weighting scheme**	w = 1/[σ^2(F_o^2)+(0.0717P)^2+0.5831P]<br>where P = (F_o^2+2F_c^2)/3	
**Largest diff. peak and hole**	0.386 and –0.258 $eÅ^{-3}$	
**RMS deviation from mean**	0.057 $eÅ^{-3}$	

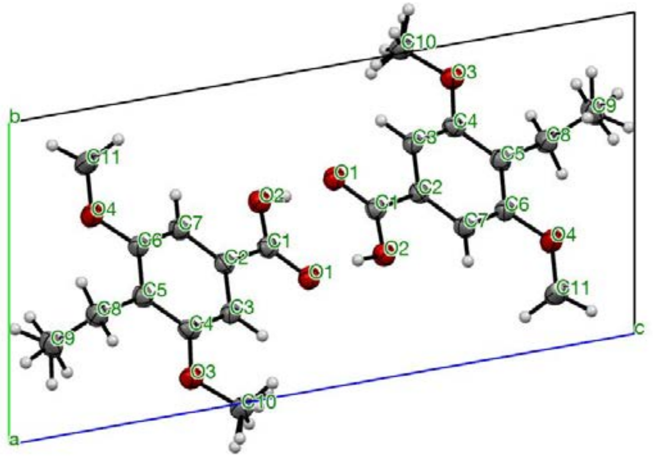

Figure S45: ORTEP thermal ellipsoid plot of the unit cell of **c** along the crystallographic *a* axis at a 50% probability level.

H. Computational results

The relative energies (energy profile) of the conformations of tetra-*endo*-tetra acid **2'** were calculated using a gas-phase semi-empirical restricted Hartree-Fock PM6[15] model on Spartan'14.[16] The host molecule **2'** (Figure S37) used in the calculations is a derivative of **2**, such that the pendent propanoic acid moieties at the base were truncated to methyl groups to simplify the calculation. Geometry optimisation calculations started at a conformation such that all pendent ethyl groups at the portal of the basket were pointing in (dihedral angle φ ≐ 0°). Subsequent optimisation calculations involved a dihedral move of all four ethyl groups by 5° in the same direction relative to the C_{4v} symmetry axis.

Table S 5: Torsion angles and relative energies in kJ mol^{-1} from 0° to 360°.

Torsion φ (°)	rel. E (kJ/mol)	Torsion φ (°)	rel. E (kJ/mol)	Torsion φ (°)	rel. E (kJ/mol)
0	0	140	16.37	280	46.72
5	1.22	145	12.45	285	46.13
10	4.24	150	9.79	290	45.05
15	8.82	155	8.52	295	43.5
20	14.31	160	8.43	300	41.54
25	19.77	165	9.01	305	39.21
30	24.53	170	9.73	310	36.56
35	28.61	175	10.22	315	33.6
40	32.19	180	10.33	320	30.31
45	35.38	185	10.03	325	26.62
50	38.19	190	9.39	330	22.42
55	40.64	195	8.68	335	17.57
60	42.68	200	8.24	340	12.27
65	44.32	205	8.54	345	7.18
70	45.51	210	9.92	350	3.13
75	46.24	215	12.58	355	0.66
80	46.5	220	16.41	360	0.02
85	46.32	225	21.2		
90	45.74	230	26.48		
95	44.8	235	31.45		
100	43.54	240	35.45		
105	41.96	245	38.33		
110	40.14	250	40.64		
115	37.91	255	42.54		
120	35.06	260	44.16		
125	31.18	265	45.46		
130	26.33	270	46.34		
135	21.18	275	46.79		

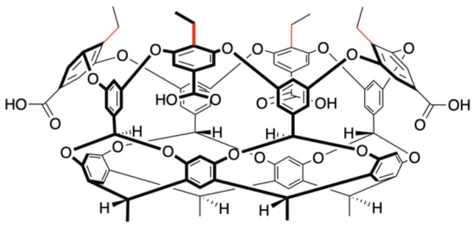

Figure S46: TEEtOA derivative **2'** used in calculations. Bonds rotated are highlighted in red.

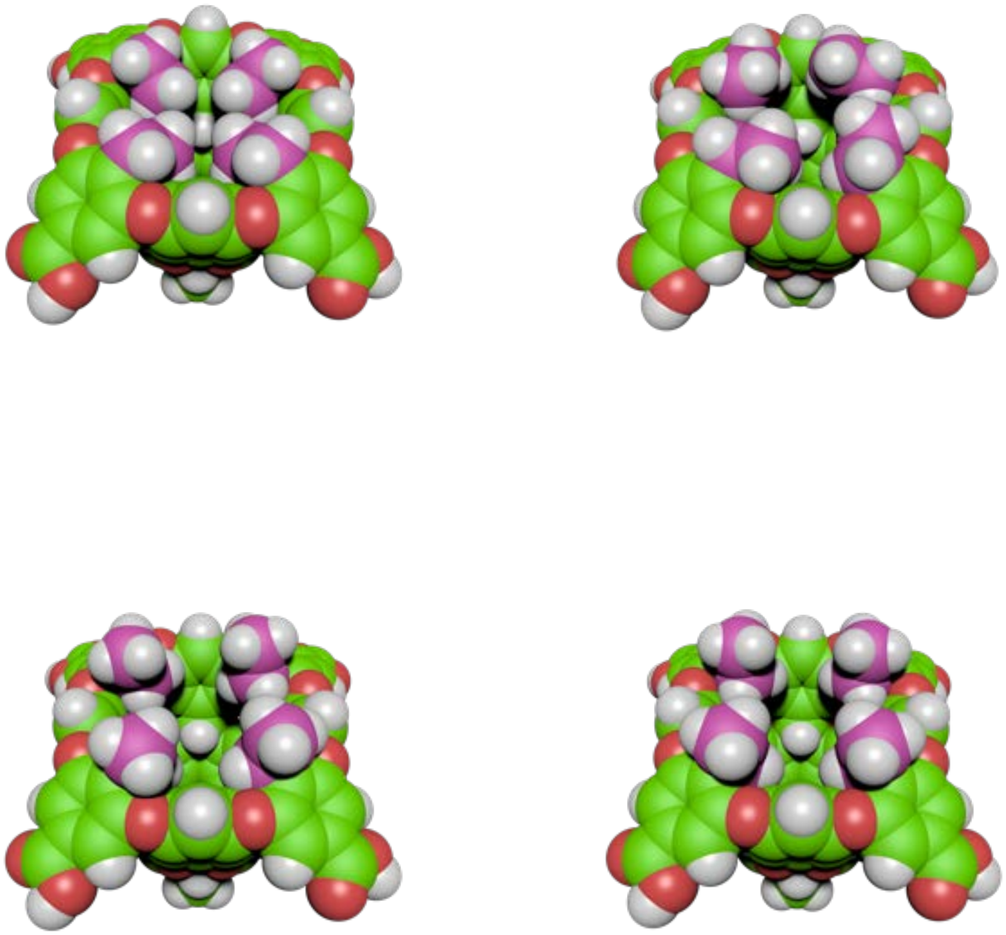

Figure S47: (top right to bottom left): Representative van der Waals structures of **2'** at different critical points in the energy profile as the four ethyl groups are simultaneously rotated out of the pocket: 0°, 80°, 160°, and 180°. The ethyl groups undergoing rotation are highlighted in pink. van der Waals structures were generated using the ePMV plugin for Cinema4D.[17]

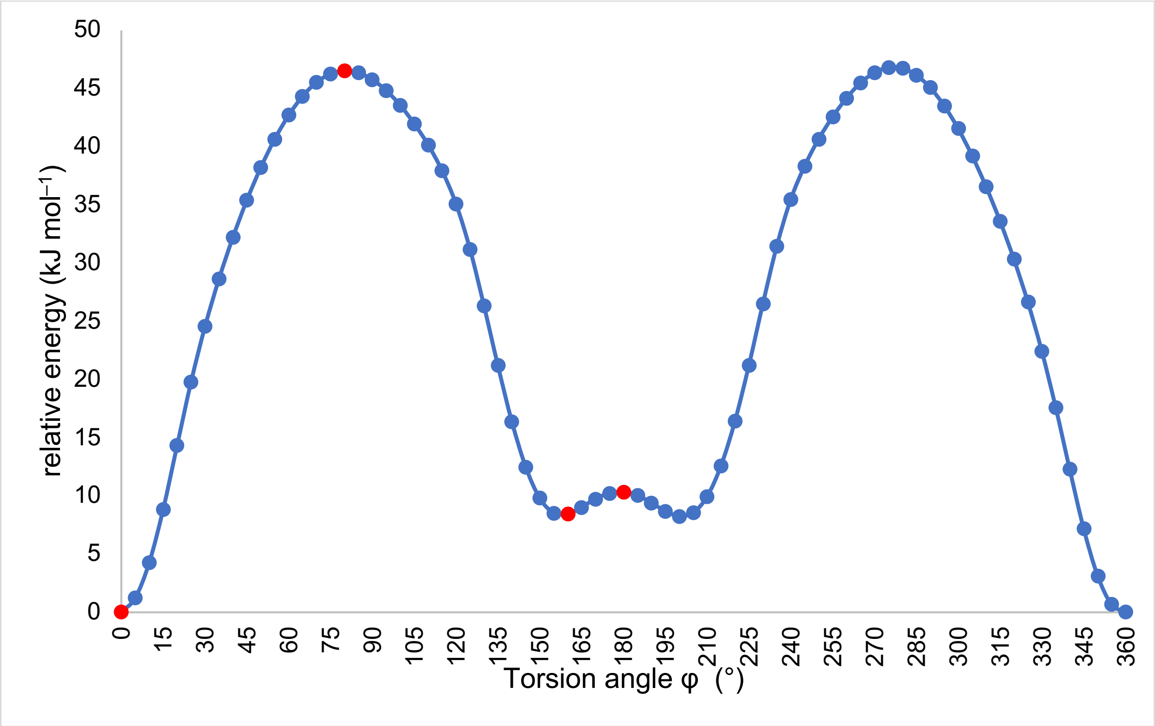

Figure S48: Plot of relative energies of **2'** versus torsion angle φ. Critical points whose coordinates are listed below are highlighted in red.

Table S 6: Optimised (PM6) Cartesian coordinates of **2'** at φ = 0°.

H	4.519	-0.764	0.893
C	3.97	-0.673	1.828
C	2.598	-0.443	4.274
C	3.405	-1.787	2.449
C	3.803	0.565	2.452
C	3.121	0.712	3.675
C	2.713	-1.705	3.673
H	2.068	-0.355	5.224
C	2.096	-2.962	4.254
H	2.72	-3.839	3.893
C	2.951	2.1	4.259
H	3.829	2.724	3.899
C	0.708	-3.13	3.668
C	-1.79	-3.411	2.439
C	0.562	-3.809	2.443
C	-0.448	-2.609	4.267
C	-1.709	-2.722	3.664
C	-0.675	-3.974	1.818
H	-0.36	-2.081	5.219
H	-0.765	-4.521	0.881
C	1.696	2.717	3.674
C	-0.571	3.805	2.446
C	0.433	2.603	4.272
C	1.781	3.408	2.45
C	0.668	3.972	1.825
C	-0.721	3.125	3.67
H	0.342	2.074	5.223
H	0.762	4.52	0.89
C	-2.111	2.956	4.25
H	-2.734	3.834	3.888
C	-2.966	-2.106	4.245
H	-3.843	-2.73	3.882
C	-3.134	-0.717	3.663
C	-3.414	1.784	2.44
C	-3.811	-0.568	2.437
C	-2.613	0.437	4.265
C	-2.726	1.7	3.666
C	-3.976	0.67	1.815
H	-2.087	0.347	5.217

H	-4.522	0.763	0.878
C	2.979	2.121	5.786
H	2.167	1.543	6.238
C	2.115	-2.993	5.781
H	3.143	-2.916	6.158
O	-3.547	3.005	1.762
O	-1.678	4.333	1.767
O	-4.338	-1.676	1.758
O	3.541	-3.007	1.771
O	1.672	-4.336	1.767
O	-3.009	-3.545	1.759
C	2.368	-3.335	0.996
H	1.729	-2.435	0.837
C	-3.336	-2.371	0.987
H	-2.436	-1.731	0.83
O	4.331	1.674	1.776
C	3.332	2.369	1.002
H	2.433	1.73	0.841
O	3.003	3.543	1.775
C	-2.372	3.333	0.993
H	-1.732	2.434	0.834
C	3.924	2.789	-0.325
C	4.699	3.339	-2.977
C	3.406	3.917	-0.966
C	4.827	1.935	-0.962
C	5.196	2.228	-2.283
C	3.804	4.168	-2.288
H	2.737	4.592	-0.434
H	5.25	1.087	-0.425
C	-2.788	3.923	-0.336
C	-3.332	4.696	-2.991
C	-1.933	4.826	-0.972
C	-3.915	3.405	-0.98
C	-4.162	3.801	-2.303
C	-2.222	5.193	-2.294
H	-1.086	5.25	-0.434
H	-4.591	2.736	-0.448
C	-3.922	-2.789	-0.343
C	-4.689	-3.335	-2.999
C	-4.824	-1.934	-0.982

C	-3.402	-3.916	-0.984
C	-3.796	-4.165	-2.308
C	-5.188	-2.225	-2.305
H	-5.249	-1.086	-0.445
H	-2.735	-4.591	-0.45
C	2.79	-3.923	-0.332
C	3.342	-4.692	-2.986
C	3.919	-3.403	-0.971
C	1.936	-4.825	-0.972
C	2.23	-5.19	-2.294
C	4.17	-3.798	-2.293
H	4.592	-2.736	-0.435
H	1.087	-5.249	-0.437
O	6.057	1.443	-3.037
O	-1.436	6.054	-3.047
O	-5.222	3.332	-3.065
O	5.233	-3.328	-3.051
O	-6.046	-1.439	-3.061
O	-3.325	-5.225	-3.068
O	1.447	-6.049	-3.05
O	3.335	5.229	-3.048
C	-5.922	2.207	-2.586
C	-7.502	0.034	-1.846
C	-5.499	0.924	-2.982
C	-7.086	2.435	-1.845
C	-7.874	1.336	-1.483
C	-6.33	-0.143	-2.586
H	-7.377	3.454	-1.564
H	-8.12	-0.828	-1.567
C	-2.201	-5.925	-2.585
C	-0.03	-7.504	-1.838
C	-0.916	-5.502	-2.978
C	-2.431	-7.088	-1.844
C	-1.332	-7.876	-1.477
C	0.15	-6.332	-2.578
H	-3.45	-7.378	-1.565
H	0.832	-8.121	-1.556
C	5.932	-2.204	-2.568
C	7.509	-0.032	-1.819
C	7.092	-2.433	-1.823

C	5.51	-0.92	-2.964
C	6.339	0.147	-2.563
C	7.88	-1.334	-1.456
H	7.382	-3.452	-1.543
H	8.125	0.83	-1.536
C	2.21	5.929	-2.569
C	0.036	7.507	-1.828
C	0.927	5.507	-2.967
C	2.437	7.09	-1.825
C	1.338	7.878	-1.462
C	-0.14	6.336	-2.57
H	3.456	7.38	-1.543
H	-0.826	8.124	-1.548
H	1.537	-2.182	6.235
C	-3	-2.129	5.772
C	-2.135	2.985	5.777
H	3.925	1.71	6.163
H	-1.559	2.174	6.232
H	-1.725	3.932	6.154
H	-2.922	-3.159	6.147
H	-3.947	-1.718	6.146
C	9.129	-1.512	-0.674
C	-1.511	-9.128	-0.699
C	-9.127	1.513	-0.705
C	1.514	9.129	-0.681
O	9.919	-0.677	-0.302
O	-0.676	-9.918	-0.328
O	-9.917	0.677	-0.337
O	0.677	9.919	-0.312
H	-4.98	-3.543	-4.027
H	-3.539	4.99	-4.018
H	4.995	3.548	-4.004
H	3.553	-4.985	-4.012
C	-0.698	-4.268	-3.799
H	0.188	-4.41	-4.458
H	-1.565	-4.119	-4.483
C	4.278	-0.703	-3.788
H	4.422	0.184	-4.447
H	4.131	-1.57	-4.471
C	0.712	4.273	-3.791

H	1.581	4.125	-4.472
H	-0.172	4.416	-4.452
C	-4.264	0.708	-3.802
H	-4.115	1.576	-4.484
H	-4.406	-0.177	-4.463
C	3.035	-0.509	-2.922
H	3.135	0.362	-2.262
H	2.841	-1.385	-2.292
H	2.149	-0.349	-3.547
C	0.516	3.032	-2.923
H	1.391	2.839	-2.29
H	0.359	2.144	-3.547
H	-0.357	3.132	-2.266
C	-3.024	0.513	-2.932
H	-2.136	0.354	-3.554
H	-3.127	-0.359	-2.274
H	-2.832	1.389	-2.3
C	-0.506	-3.027	-2.93
H	0.365	-3.129	-2.27
H	-1.383	-2.835	-2.3
H	-0.346	-2.14	-3.553
H	1.703	-3.94	6.155
H	-2.189	-1.552	6.229
H	-3.165	2.907	6.151
H	2.9	3.15	6.162
O	-2.838	-9.34	-0.399
H	-2.976	-10.177	0.127
O	9.341	-2.838	-0.373
H	10.176	-2.977	0.156
O	2.839	9.341	-0.377
H	2.976	10.177	0.15
O	-9.34	2.839	-0.402
H	-10.177	2.976	0.123

Table S 7: Optimised (PM6) Cartesian coordinates of **2'** at φ = 80°.

H	4.518	-0.757	0.924
C	3.97	-0.666	1.859
C	2.598	-0.441	4.305
C	3.406	-1.782	2.479
C	3.801	0.57	2.484
C	3.12	0.715	3.708
C	2.715	-1.702	3.703
H	2.068	-0.353	5.256
C	2.099	-2.959	4.285
H	2.725	-3.836	3.923
C	2.948	2.104	4.291
H	3.825	2.729	3.931
C	0.711	-3.129	3.7
C	-1.785	-3.413	2.469
C	0.567	-3.808	2.475
C	-0.445	-2.609	4.298
C	-1.706	-2.724	3.695
C	-0.669	-3.974	1.848
H	-0.359	-2.082	5.25
H	-0.758	-4.52	0.912
C	1.692	2.719	3.705
C	-0.576	3.804	2.478
C	0.429	2.603	4.303
C	1.776	3.409	2.48
C	0.662	3.971	1.857
C	-0.725	3.124	3.702
H	0.34	2.074	5.255
H	0.755	4.519	0.921
C	-2.115	2.953	4.282
H	-2.739	3.83	3.919
C	-2.963	-2.11	4.277
H	-3.84	-2.735	3.913
C	-3.133	-0.721	3.695
C	-3.415	1.779	2.47
C	-3.811	-0.573	2.47
C	-2.614	0.434	4.296
C	-2.728	1.696	3.696
C	-3.977	0.664	1.846
H	-2.087	0.346	5.249

H	-4.522	0.755	0.909
C	2.975	2.125	5.818
H	2.164	1.546	6.27
C	2.119	-2.991	5.812
H	3.148	-2.911	6.189
O	-3.551	2.999	1.791
O	-1.685	4.331	1.801
O	-4.336	-1.683	1.793
O	3.544	-3.001	1.799
O	1.678	-4.333	1.801
O	-3.004	-3.549	1.788
C	2.372	-3.333	1.026
H	1.731	-2.436	0.863
C	-3.335	-2.375	1.017
H	-2.438	-1.734	0.857
O	4.329	1.681	1.811
C	3.331	2.374	1.033
H	2.434	1.732	0.868
O	2.997	3.546	1.804
C	-2.376	3.331	1.023
H	-1.734	2.434	0.861
C	3.929	2.796	-0.29
C	4.74	3.35	-2.93
C	3.375	3.892	-0.958
C	4.88	1.973	-0.898
C	5.264	2.268	-2.214
C	3.788	4.145	-2.273
H	2.659	4.535	-0.449
H	5.324	1.147	-0.343
C	-2.795	3.928	-0.302
C	-3.342	4.736	-2.945
C	-1.97	4.878	-0.909
C	-3.889	3.373	-0.972
C	-4.139	3.785	-2.289
C	-2.261	5.26	-2.226
H	-1.146	5.322	-0.353
H	-4.533	2.657	-0.464
C	-3.928	-2.796	-0.308
C	-4.729	-3.346	-2.952
C	-4.876	-1.971	-0.919

C	-3.371	-3.891	-0.975
C	-3.78	-4.142	-2.292
C	-5.256	-2.264	-2.236
H	-5.322	-1.146	-0.364
H	-2.657	-4.535	-0.464
C	2.796	-3.928	-0.298
C	3.353	-4.731	-2.94
C	3.893	-3.371	-0.963
C	1.973	-4.876	-0.91
C	2.269	-5.257	-2.226
C	4.147	-3.781	-2.279
H	4.535	-2.657	-0.451
H	1.146	-5.321	-0.357
O	6.168	1.5	-2.941
O	-1.492	6.164	-2.952
O	-5.141	3.26	-3.093
O	5.152	-3.256	-3.079
O	-6.157	-1.496	-2.966
O	-3.254	-5.146	-3.094
O	1.502	-6.159	-2.957
O	3.265	5.149	-3.076
C	-5.853	2.147	-2.602
C	-7.48	0.06	-1.708
C	-5.522	0.845	-3.016
C	-6.958	2.432	-1.785
C	-7.773	1.386	-1.349
C	-6.374	-0.175	-2.52
H	-7.176	3.47	-1.502
H	-8.11	-0.77	-1.365
C	-2.141	-5.856	-2.6
C	-0.055	-7.481	-1.7
C	-0.839	-5.525	-3.012
C	-2.427	-6.96	-1.781
C	-1.381	-7.774	-1.343
C	0.181	-6.377	-2.514
H	-3.465	-7.178	-1.499
H	0.774	-8.11	-1.355
C	5.862	-2.143	-2.584
C	7.487	-0.058	-1.68
C	6.964	-2.43	-1.762

C	5.533	-0.841	-2.997
C	6.384	0.179	-2.497
C	7.778	-1.384	-1.322
H	7.182	-3.467	-1.48
H	8.115	0.771	-1.335
C	2.15	5.859	-2.585
C	0.062	7.484	-1.689
C	0.849	5.529	-3.001
C	2.434	6.962	-1.764
C	1.387	7.776	-1.328
C	-0.172	6.38	-2.505
H	3.471	7.18	-1.479
H	-0.768	8.113	-1.347
H	1.54	-2.18	6.266
C	-2.996	-2.134	5.804
C	-2.14	2.982	5.809
H	3.922	1.715	6.195
H	-1.563	2.171	6.264
H	-1.731	3.929	6.186
H	-2.917	-3.163	6.178
H	-3.944	-1.724	6.178
C	8.964	-1.635	-0.466
C	-1.632	-8.962	-0.489
C	-8.962	1.635	-0.497
C	1.635	8.963	-0.472
O	9.757	-0.843	-0.016
O	-0.84	-9.758	-0.043
O	-9.758	0.843	-0.051
O	0.842	9.758	-0.026
H	-5.039	-3.559	-3.973
H	-3.553	5.048	-3.966
H	5.054	3.564	-3.95
H	3.568	-5.042	-3.96
C	-0.431	-4.426	-3.947
H	0.439	-3.884	-3.508
H	-0.026	-4.911	-4.87
C	4.436	-0.433	-3.934
H	3.893	0.437	-3.496
H	4.923	-0.027	-4.855
C	0.444	4.43	-3.937

H	0.041	4.915	-4.861
H	-0.427	3.888	-3.501
C	-4.421	0.439	-3.949
H	-4.905	0.034	-4.873
H	-3.879	-0.432	-3.511
C	3.429	-1.5	-4.331
H	2.837	-1.845	-3.475
H	3.923	-2.383	-4.764
H	2.73	-1.118	-5.084
C	1.513	3.422	-4.328
H	2.397	3.916	-4.76
H	1.133	2.723	-5.082
H	1.856	2.832	-3.471
C	-3.413	1.507	-4.341
H	-2.712	1.125	-5.093
H	-2.824	1.85	-3.483
H	-3.906	2.39	-4.774
C	-1.499	-3.418	-4.341
H	-1.843	-2.827	-3.484
H	-2.383	-3.912	-4.773
H	-1.117	-2.718	-5.094
H	1.709	-3.938	6.186
H	-2.187	-1.556	6.26
H	-3.17	2.902	6.182
H	2.895	3.154	6.193
O	-2.973	-9.102	-0.215
H	-3.16	-9.894	0.361
O	9.106	-2.977	-0.197
H	9.897	-3.165	0.381
O	2.976	9.105	-0.198
H	3.162	9.897	0.38
O	-9.104	2.977	-0.224
H	-9.897	3.164	0.351

Table S 8: Optimised (PM6) Cartesian coordinates of **2'** at φ = 160°.

H	4.522	-0.76	0.92
C	3.972	-0.668	1.854
C	2.599	-0.44	4.299
C	3.408	-1.783	2.475
C	3.803	0.569	2.477
C	3.121	0.716	3.7
C	2.715	-1.701	3.699
H	2.069	-0.351	5.25
C	2.099	-2.959	4.278
H	2.724	-3.835	3.917
C	2.949	2.104	4.283
H	3.827	2.729	3.923
C	0.711	-3.13	3.693
C	-1.787	-3.414	2.464
C	0.565	-3.808	2.468
C	-0.446	-2.61	4.292
C	-1.706	-2.724	3.689
C	-0.671	-3.976	1.843
H	-0.359	-2.082	5.244
H	-0.761	-4.524	0.908
C	1.693	2.72	3.7
C	-0.574	3.806	2.472
C	0.43	2.604	4.297
C	1.778	3.412	2.476
C	0.664	3.975	1.852
C	-0.724	3.125	3.695
H	0.34	2.075	5.248
H	0.758	4.524	0.918
C	-2.114	2.953	4.275
H	-2.738	3.83	3.913
C	-2.965	-2.11	4.27
H	-3.841	-2.734	3.905
C	-3.134	-0.72	3.688
C	-3.417	1.78	2.466
C	-3.812	-0.572	2.462
C	-2.615	0.434	4.29
C	-2.728	1.697	3.691
C	-3.979	0.667	1.841
H	-2.088	0.344	5.243

H	-4.526	0.76	0.905
C	2.978	2.125	5.811
H	2.166	1.547	6.264
C	2.118	-2.991	5.805
H	3.146	-2.913	6.183
O	-3.547	3	1.786
O	-1.682	4.329	1.788
O	-4.333	-1.679	1.778
O	3.541	-3.002	1.794
O	1.675	-4.33	1.787
O	-3.004	-3.545	1.781
C	2.366	-3.323	1.02
H	1.726	-2.423	0.869
C	-3.325	-2.369	1.01
H	-2.425	-1.728	0.861
O	4.326	1.678	1.796
C	3.321	2.368	1.025
H	2.422	1.728	0.873
O	2.998	3.544	1.797
C	-2.37	3.323	1.017
H	-1.729	2.423	0.867
C	3.904	2.784	-0.306
C	4.686	3.345	-2.953
C	3.4	3.923	-0.938
C	4.794	1.92	-0.951
C	5.166	2.219	-2.269
C	3.805	4.182	-2.256
H	2.741	4.603	-0.4
H	5.205	1.062	-0.422
C	-2.783	3.904	-0.317
C	-3.339	4.679	-2.966
C	-1.917	4.791	-0.962
C	-3.921	3.398	-0.95
C	-4.178	3.801	-2.268
C	-2.214	5.16	-2.282
H	-1.059	5.203	-0.433
H	-4.602	2.741	-0.411
C	-3.904	-2.784	-0.324
C	-4.677	-3.343	-2.974
C	-4.792	-1.919	-0.972

C	-3.398	-3.923	-0.955
C	-3.799	-4.181	-2.274
C	-5.158	-2.218	-2.292
H	-5.204	-1.061	-0.444
H	-2.741	-4.603	-0.415
C	2.784	-3.903	-0.313
C	3.349	-4.676	-2.961
C	3.925	-3.398	-0.941
C	1.92	-4.789	-0.962
C	2.222	-5.157	-2.281
C	4.186	-3.799	-2.259
H	4.605	-2.742	-0.399
H	1.06	-5.201	-0.436
O	6.007	1.429	-3.038
O	-1.421	5.999	-3.051
O	-5.267	3.366	-3.01
O	5.278	-3.364	-2.996
O	-5.997	-1.426	-3.063
O	-3.363	-5.271	-3.014
O	1.431	-5.994	-3.055
O	3.372	5.272	-2.996
C	-5.95	2.219	-2.56
C	-7.511	0.012	-1.883
C	-5.488	0.945	-2.948
C	-7.14	2.421	-1.856
C	-7.919	1.304	-1.524
C	-6.312	-0.139	-2.587
H	-7.459	3.432	-1.579
H	-8.121	-0.863	-1.63
C	-2.216	-5.953	-2.563
C	-0.009	-7.511	-1.881
C	-0.941	-5.486	-2.942
C	-2.418	-7.145	-1.863
C	-1.301	-7.923	-1.529
C	0.143	-6.311	-2.581
H	-3.43	-7.467	-1.592
H	0.866	-8.12	-1.627
C	5.96	-2.218	-2.543
C	7.516	-0.011	-1.854
C	7.147	-2.42	-1.834

C	5.498	-0.943	-2.928
C	6.321	0.141	-2.562
C	7.924	-1.304	-1.496
H	7.466	-3.432	-1.559
H	8.124	0.864	-1.596
C	2.224	5.955	-2.548
C	0.016	7.513	-1.871
C	0.95	5.49	-2.933
C	2.424	7.146	-1.845
C	1.307	7.924	-1.513
C	-0.135	6.314	-2.574
H	3.436	7.466	-1.57
H	-0.86	8.123	-1.618
H	1.539	-2.181	6.26
C	-2.999	-2.133	5.796
C	-2.139	2.983	5.802
H	3.925	1.715	6.187
H	-1.561	2.172	6.257
H	-1.729	3.93	6.179
H	-2.921	-3.163	6.171
H	-3.947	-1.723	6.17
C	9.201	-1.454	-0.753
C	-1.451	-9.205	-0.795
C	-9.199	1.453	-0.786
C	1.455	9.205	-0.777
O	9.986	-0.601	-0.413
O	-0.598	-9.992	-0.458
O	-9.986	0.6	-0.45
O	0.602	9.991	-0.441
H	-4.973	-3.557	-4
H	-3.551	4.977	-3.991
H	4.986	3.56	-3.977
H	3.565	-4.973	-3.986
C	-0.756	-4.211	-3.707
H	-1.297	-3.386	-3.193
H	0.316	-3.918	-3.72
C	4.229	-0.758	-3.704
H	3.399	-1.296	-3.196
H	3.939	0.315	-3.722
C	0.767	4.217	-3.702

H	-0.306	3.926	-3.721
H	1.305	3.39	-3.19
C	-4.215	0.761	-3.716
H	-3.926	-0.312	-3.737
H	-3.387	1.295	-3.201
C	4.383	-1.272	-5.134
H	4.655	-2.338	-5.147
H	5.167	-0.728	-5.675
H	3.452	-1.159	-5.7
C	1.283	4.365	-5.133
H	0.743	5.151	-5.676
H	1.164	3.433	-5.696
H	2.35	4.629	-5.146
C	-4.359	1.281	-5.146
H	-3.43	1.152	-5.711
H	-4.612	2.352	-5.154
H	-5.153	0.753	-5.688
C	-1.267	-4.353	-5.14
H	-2.331	-4.632	-5.157
H	-0.716	-5.127	-5.688
H	-1.159	-3.415	-5.695
H	1.706	-3.939	6.179
H	-2.189	-1.556	6.253
H	-3.168	2.904	6.176
H	2.899	3.154	6.186
O	-2.771	-9.455	-0.495
H	-2.89	-10.312	0
O	9.447	-2.773	-0.45
H	10.301	-2.892	0.051
O	2.775	9.454	-0.476
H	2.893	10.311	0.021
O	-9.446	2.773	-0.482
H	-10.302	2.891	0.016

Table S 9: Optimised (PM6) Cartesian coordinates of **2'** at φ = 180°.

H	4.523	-0.756	0.92
C	3.973	-0.665	1.854
C	2.599	-0.438	4.298
C	3.41	-1.78	2.474
C	3.802	0.572	2.476
C	3.119	0.718	3.7
C	2.716	-1.699	3.697
H	2.068	-0.35	5.248
C	2.101	-2.957	4.277
H	2.727	-3.833	3.916
C	2.946	2.107	4.282
H	3.823	2.732	3.923
C	0.713	-3.129	3.692
C	-1.783	-3.416	2.462
C	0.569	-3.808	2.466
C	-0.444	-2.61	4.29
C	-1.704	-2.726	3.687
C	-0.667	-3.977	1.841
H	-0.357	-2.082	5.242
H	-0.756	-4.525	0.906
C	1.69	2.721	3.698
C	-0.578	3.805	2.469
C	0.427	2.605	4.295
C	1.774	3.412	2.474
C	0.66	3.975	1.849
C	-0.728	3.124	3.693
H	0.336	2.076	5.246
H	0.753	4.523	0.914
C	-2.118	2.952	4.272
H	-2.743	3.828	3.909
C	-2.963	-2.112	4.267
H	-3.838	-2.737	3.903
C	-3.134	-0.723	3.685
C	-3.418	1.777	2.461
C	-3.811	-0.576	2.459
C	-2.616	0.432	4.287
C	-2.731	1.694	3.688
C	-3.979	0.662	1.837
H	-2.089	0.344	5.24

H	-4.525	0.754	0.901
C	2.974	2.128	5.809
H	2.162	1.549	6.262
C	2.12	-2.989	5.804
H	3.148	-2.91	6.182
O	-3.551	2.996	1.781
O	-1.686	4.327	1.786
O	-4.332	-1.685	1.777
O	3.545	-2.999	1.793
O	1.68	-4.33	1.788
O	-3	-3.549	1.778
C	2.371	-3.323	1.019
H	1.73	-2.424	0.866
C	-3.324	-2.373	1.007
H	-2.425	-1.732	0.857
O	4.325	1.682	1.797
C	3.321	2.371	1.025
H	2.422	1.729	0.871
O	2.994	3.546	1.795
C	-2.374	3.322	1.013
H	-1.732	2.423	0.861
C	3.906	2.787	-0.306
C	4.689	3.343	-2.954
C	3.387	3.916	-0.947
C	4.81	1.933	-0.942
C	5.184	2.231	-2.261
C	3.791	4.17	-2.266
H	2.715	4.588	-0.415
H	5.232	1.084	-0.406
C	-2.787	3.905	-0.32
C	-3.337	4.686	-2.969
C	-1.932	4.81	-0.954
C	-3.913	3.385	-0.963
C	-4.165	3.788	-2.282
C	-2.227	5.183	-2.274
H	-1.085	5.233	-0.417
H	-4.586	2.714	-0.432
C	-3.905	-2.788	-0.326
C	-4.681	-3.341	-2.976
C	-4.809	-1.934	-0.963

C	-3.384	-3.915	-0.967
C	-3.785	-4.169	-2.286
C	-5.179	-2.23	-2.283
H	-5.232	-1.086	-0.427
H	-2.714	-4.588	-0.434
C	2.79	-3.905	-0.312
C	3.351	-4.681	-2.961
C	3.921	-3.386	-0.949
C	1.936	-4.806	-0.953
C	2.236	-5.177	-2.272
C	4.178	-3.787	-2.268
H	4.593	-2.717	-0.413
H	1.084	-5.227	-0.421
O	6.051	1.452	-3.014
O	-1.446	6.049	-3.026
O	-5.229	3.325	-3.043
O	5.247	-3.326	-3.022
O	-6.044	-1.45	-3.037
O	-3.321	-5.233	-3.045
O	1.457	-6.039	-3.03
O	3.33	5.236	-3.025
C	-5.931	2.199	-2.573
C	-7.516	0.024	-1.849
C	-5.497	0.914	-2.954
C	-7.106	2.426	-1.851
C	-7.898	1.327	-1.497
C	-6.333	-0.153	-2.571
H	-7.404	3.446	-1.579
H	-8.137	-0.838	-1.577
C	-2.193	-5.934	-2.574
C	-0.017	-7.517	-1.85
C	-0.909	-5.495	-2.949
C	-2.42	-7.112	-1.856
C	-1.32	-7.902	-1.502
C	0.16	-6.331	-2.567
H	-3.44	-7.413	-1.588
H	0.846	-8.136	-1.578
C	5.946	-2.198	-2.551
C	7.526	-0.02	-1.827
C	7.122	-2.423	-1.83

C	5.508	-0.914	-2.93
C	6.342	0.155	-2.547
C	7.911	-1.321	-1.475
H	7.422	-3.442	-1.558
H	8.145	0.844	-1.555
C	2.202	5.938	-2.556
C	0.026	7.523	-1.838
C	0.918	5.502	-2.938
C	2.428	7.114	-1.835
C	1.328	7.906	-1.484
C	-0.15	6.339	-2.558
H	3.447	7.412	-1.562
H	-0.837	8.144	-1.568
H	1.54	-2.179	6.258
C	-2.997	-2.135	5.794
C	-2.143	2.982	5.799
H	3.92	1.718	6.186
H	-1.566	2.171	6.254
H	-1.735	3.929	6.176
H	-2.918	-3.165	6.169
H	-3.945	-1.726	6.168
C	9.177	-1.495	-0.72
C	-1.496	-9.171	-0.75
C	-9.164	1.503	-0.74
C	1.503	9.173	-0.729
O	9.972	-0.657	-0.364
O	-0.658	-9.964	-0.392
O	-9.958	0.667	-0.383
O	0.665	9.967	-0.374
H	-4.977	-3.553	-4.002
H	-3.547	4.984	-3.995
H	4.988	3.555	-3.979
H	3.566	-4.978	-3.986
C	-0.695	-4.244	-3.745
H	-1.482	-3.496	-3.505
H	0.275	-3.775	-3.471
C	4.257	-0.702	-3.729
H	3.51	-1.489	-3.488
H	3.788	0.268	-3.458
C	0.705	4.252	-3.736

H	-0.267	3.786	-3.466
H	1.488	3.501	-3.492
C	-4.25	0.7	-3.757
H	-3.784	-0.273	-3.489
H	-3.498	1.482	-3.518
C	4.566	-0.726	-5.226
H	4.995	-1.69	-5.53
H	5.288	0.056	-5.498
H	3.661	-0.564	-5.82
C	0.733	4.559	-5.232
H	-0.048	5.281	-5.507
H	0.571	3.653	-5.826
H	1.698	4.986	-5.536
C	-4.565	0.724	-5.252
H	-3.661	0.562	-5.85
H	-4.995	1.688	-5.556
H	-5.287	-0.058	-5.522
C	-0.715	-4.55	-5.243
H	-1.674	-4.99	-5.549
H	0.076	-5.26	-5.517
H	-0.564	-3.641	-5.835
H	1.709	-3.937	6.178
H	-2.188	-1.557	6.251
H	-3.173	2.902	6.172
H	2.893	3.157	6.185
O	-2.822	-9.397	-0.462
H	-2.959	-10.245	0.045
O	9.4	-2.821	-0.423
H	10.247	-2.956	0.088
O	2.829	9.396	-0.435
H	2.965	10.243	0.074
O	-9.385	2.829	-0.446
H	-10.231	2.966	0.064

I. Molecular dynamics simulations

General considerations

Molecular dynamics (MD) simulations were performed using the GROMACS 2016.3 simulation package.[18, 19] Cavitands **1** and **2**, and guest G2 were modelled using the Generalized AMBER Force-Field (GAFF)[20] with charges obtained from AM1-BCC calculations.[21] The eight carboxylic acid groups on each of the host molecules were deprotonated such that the net charge on the host is –8e, matching the expected protonation state of the molecules at the experimental conditions. The charges were then balanced using 8 sodium cations as counterions. Guest **G2** was the guest employed in these simulations, and its protonation state matches that of experimental conditions, and balanced with sodium as a counterion. Water was modelled using the TIP4P-Ew potential.[22] Charges on the cavitands and their counterions were scaled to 75% of their full value to account for polarisation effects. Not all charges were scaled, however; the scaled charges were localised around the atoms carrying the negative charge. Simulations were conducted in the isothermal–isobaric ensemble at 25 °C and 1 bar—the Nosé–Hoover thermostat[23, 24] and the Rahman–Parrinello barostat[25] were used to maintain the temperature and pressure, respectively. Non-bonded Lennard-Jones interactions were truncated beyond a separation of 9 Å, with a mean-field dispersion correction for longer-range contributions to the energy and pressure. Electrostatic interactions were evaluated using Particle Mesh Ewald Summation with a real space cutoff of 9 Å.[26] Bonds involving hydrogens for the cavitands and guest were constrained using the LINCS algorithm,[27] while water was held rigid using SETTLE.[28] The equations of motion were integrated using a time step of 2 fs.

Pocket hydration state

Cavitands **1** and **2** were each placed in a bath of 2500 water molecules to probe the hydration number probability distribution in the individual host pockets. The simulations were conducted for 20 ns following at least 4 ns for equilibration. In addition to the equilibrium conformation of **2**, i.e., all ethyl groups pointing inwards, five additional conformations were investigated to probe the wetting/de-wetting of the pocket as a function of conformation: one ethyl group out (**2-1o**), two adjacent ethyl groups out (**2-2o-*cis***), two opposite ethyl groups out (**2-2o-*trans***), three ethyl groups out (**2-3o**), and four ethyl groups out (**2-4o**). Dihedral restraints were applied to the relevant atoms to generate the individual conformations.

Volume calculations

The partial molar volume ($\overline{V}_i$) of **1**, **2**, and **2-4o** were calculated by immersing each host in a bath of 2500 water molecules and calculating the volume difference between the average simulation box volume of the system and the average simulation box volume of pure water with the same number of water molecules. The internal volumes of **1**, **2**, and **2-4o** were determined using Monte Carlo integration by randomly inserting 10^5 points into a box bounding the empty host, determining the fraction of points inside the cavity of the host bounded by the van der Waals volume of the cavitands per simulation snapshot, and averaging over 50000 simulation snapshots.

Potential of mean force calculations

The potential of mean force (PMF) between **2** and **G2** were evaluated along a trajectory which lies colinear with the idealised C_4 symmetry axis of **2**. The complex between **G2∈2** was solvated by 2500 water molecules in a cubic simulation box, and the cavitand was aligned with the *z*-axis of the box by applying restraint potentials on dummy atoms which lie on the C_4 axis of the cavitand. The bottom dummy atom was determined as the centre of the ring of carbon atoms attached to proton H_a, and the top dummy atom was determined to be the centre of the ethereal oxygen atoms at the portal of the cavity (see Fig. **S14**). The bottom dummy atom was spatially

restrained with a harmonic force constant of 1 × 10^5 kJ mol^{-1} nm^{-2}; the vector connecting the top dummy atom and the bottom dummy atom to the *z*-axis was fixed using a harmonic angular constraint with a force constant of 5 × 10^4 kJ mol^{-1} nm^{-2}. The centre of the aromatic ring of **G2** was restrained to the C_4 axis of **2** using a harmonic potential with a force constant of 1 × 10^5 kJ mol^{-1} nm^{-2} acting normal to the C_4 axis. The PMF was determined using umbrella sampling[29] over a series of 35 overlapping windows spanning from bulk water to the bottom of the host pocket. A force constant of 1.5 × 10^4 kJ mol^{-1} $Å^{-2}$ was used for the harmonic umbrella potential, with successive minima evenly spaced at 0.5 Å increments. Each simulation window was equilibrated for 2 ns followed by a 10 ns production run. Post-simulation analyses used 10000 saved configurations, and the PMFs were reconstructed using the Weighted Histogram Analysis Method (WHAM).[30]

Table S10: Hydration and volume parameters of hosts 1, 2, and the various conformations of 2. Reported errors represent 1σ.

Cavitand	average water number ⟨*n*⟩	Partial molar volume $\bar{V}_i$, (cm^3 mol^{-1})	Pocket volume ($Å^3$)
**1**	1.650 ± 0.370	1223 ± 6	280.5 ± 1.4
**2**	0.027 ± 0.038	1293 ± 3	266.0 ± 3.3
**2-1o**	0.067 ± 0.031	—*	—*
**2-2o-*cis***	0.419 ± 0.242	—*	—*
**2-2o-*trans***	0.059 ± 0.046	—*	—*
**2-3o**	0.753 ± 0.059	—*	—*
**2-4o**	2.362 ± 0.432	1287 ± 6	283.1 ± 1.2

J. References

1. Kakwere, H.; Payne, R. J.; Jolliffe, K. A.; Perrier, S. Self-Assembling Macromolecular Chimeras: Controlling Fibrillization of a Β-Sheet Forming Peptide by Polymer Conjugation. *Soft Matter* **2011,** *7* (8).

2. House, H. O.; Chu, C.-Y.; Wilkins, J. M.; Umen, M. J. Chemistry of Carbanions. Xxvii. Convenient Precursor for the Generation of Lithium Organocuprates. *J. Org. Chem.* **2002,** *40* (10), 1460-1469.

3. Wright, A. C.; Du, Y. E.; Stoltz, B. M. Small-Scale Procedure for Acid-Catalyzed Ketal Formation. *J. Org. Chem.* **2019,** *84* (17), 11258-11260.

4. Das, P.; McNulty, J. Dichotomous Reactivity in the Reaction of Triethyl- and Triphenylphosphane Hbr Salts with Dimethyl Acetals: A Novel Entry to Α-Alkoxy-Functionalized Ylides and General Synthesis of Vinyl Ethers and Alkoxy Dienes. *Eur. J. Org. Chem.* **2010,** *2010* (19), 3587-3591.

5. Azzena, U.; Idini, M. V.; Pilo, L. Synthesis of Antibiotic Stilbenes by Reductive Metalation of 3,4,5-Trimethoxybenzaldehyde Dimethyl Acetal. *Synth. Commun.* **2003,** *33* (8), 1309-1317.

6. Sun, H.; Gibb, C. L. D.; Gibb, B. C. Calorimetric Analysis of the 1 : 1 Complexes Formed between a Water-Soluble Deep-Cavity Cavitand, and Cyclic and Acyclic Carboxylic Acids. *Supramol. Chem.* **2008,** *20* (1-2), 141-147.

7. Turnbull, W. B.; Daranas, A. H. On the Value of C: Can Low Affinity Systems Be Studied by Isothermal Titration Calorimetry? *J. Am. Chem. Soc.* **2003,** *125* (48), 14859-14866.

8. Tellinghuisen, J. Isothermal Titration Calorimetry at Very Low C. *Anal. Biochem.* **2008,** *373* (2), 395-397.

9. Gibb, C. L.; Gibb, B. C. Binding of Cyclic Carboxylates to Octa-Acid Deep-Cavity Cavitand. *J. Comput.-Aided Mol. Des.* **2014,** *28* (4), 319-325.

10. Sullivan, M. R.; Sokkalingam, P.; Nguyen, T.; Donahue, J. P.; Gibb, B. C. Binding of Carboxylate and Trimethylammonium Salts to Octa-Acid and Temoa Deep-Cavity Cavitands. *J. Comput.-Aided Mol. Des.* **2017,** *31* (1), 21-28.

11. Suating, P.; Nguyen, T. T.; Ernst, N. E.; Wang, Y.; Jordan, J. H.; Gibb, C. L. D.; Ashbaugh, H. S.; Gibb, B. C. Proximal Charge Effects on Guest Binding to a Non-Polar Pocket. *Chem. Sci.* **2020,** *11* (14), 3656-3663.

12. Thordarson, P. Bindfit. www.supramolecular.org.

13. Brynn Hibbert, D.; Thordarson, P. The Death of the Job Plot, Transparency, Open Science and Online Tools, Uncertainty Estimation Methods and Other Developments in

Supramolecular Chemistry Data Analysis. *Chem. Commun. (Cambridge, U. K.)* **2016,** *52* (87), 12792-12805.

14. Sheldrick, G. M. Crystal Structure Refinement with Shelxl. *Acta Crystallogr., Sect. C: Cryst. Struct. Commun.* **2015,** *71* (Pt 1), 3-8.

15. Stewart, J. J. Optimization of Parameters for Semiempirical Methods V: Modification of Nddo Approximations and Application to 70 Elements. *J. Mol. Model.* **2007,** *13* (12), 1173-1213.

16. Deppmeier, B. J.; Driessen, A. J.; Hehre, T. S.; Hehre, W. J.; Johnson, J. A.; Klunzinger, P. E.; Leonard, J. M.; Pham, I. N.; Pietro, W. J.; Yu, J. *Spartan '14*, version 14.119p1.1.2; Wavefunction, Inc.: Irvine, Ca, 2014.

17. Johnson, G. T.; Autin, L.; Goodsell, D. S.; Sanner, M. F.; Olson, A. J. Epmv Embeds Molecular Modeling into Professional Animation Software Environments. *Structure* **2011,** *19* (3), 293-303.

18. Abraham, M. J.; Murtola, T.; Schulz, R.; Páll, S.; Smith, J. C.; Hess, B.; Lindahl, E. Gromacs: High Performance Molecular Simulations through Multi-Level Parallelism from Laptops to Supercomputers. *SoftwareX* **2015,** *1-2*, 19-25.

19. Páll, S.; Abraham, M. J.; Kutzner, C.; Hess, B.; Lindahl, E., Tackling Exascale Software Challenges in Molecular Dynamics Simulations with Gromacs. In *Solving Software Challenges for Exascale*, 2015; pp 3-27.

20. Siu, S. W.; Pluhackova, K.; Bockmann, R. A. Optimization of the Opls-Aa Force Field for Long Hydrocarbons. *J. Chem. Theory Comput.* **2012,** *8* (4), 1459-1470.

21. Wang, J.; Wolf, R. M.; Caldwell, J. W.; Kollman, P. A.; Case, D. A. Development and Testing of a General Amber Force Field. *J. Comput. Chem.* **2004,** *25* (9), 1157-1174.

22. Horn, H. W.; Swope, W. C.; Pitera, J. W.; Madura, J. D.; Dick, T. J.; Hura, G. L.; Head-Gordon, T. Development of an Improved Four-Site Water Model for Biomolecular Simulations: Tip4p-Ew. *J. Chem. Phys.* **2004,** *120* (20), 9665-9678.

23. Nosé, S. A Unified Formulation of the Constant Temperature Molecular Dynamics Methods. *J. Chem. Phys.* **1984,** *81* (1), 511-519.

24. Hoover, W. G. Canonical Dynamics: Equilibrium Phase-Space Distributions. *Phys. Rev. A: At., Mol., Opt. Phys.* **1985,** *31* (3), 1695-1697.

25. Parrinello, M.; Rahman, A. Polymorphic Transitions in Single-Crystals - a New Molecular-Dynamics Method. *Journal of Applied Physics* **1981,** *52* (12), 7182-7190.

26. Darden, T.; York, D.; Pedersen, L. Particle Mesh Ewald: Ann·Log(N) Method for Ewald Sums in Large Systems. *J. Chem. Phys.* **1993,** *98* (12), 10089-10092.

27. Hess, B.; Bekker, H.; Berendsen, H. J. C.; Fraaije, J. G. E. M. Lincs: A Linear Constraint Solver for Molecular Simulations. *J. Comput. Chem.* **1997,** *18* (12), 1463-1472.
28. Miyamoto, S.; Kollman, P. A. Settle - an Analytical Version of the Shake and Rattle Algorithm for Rigid Water Models. *J. Comput. Chem.* **1992,** *13* (8), 952-962.
29. Torrie, G. M.; Valleau, J. P. Nonphysical Sampling Distributions in Monte Carlo Free-Energy Estimation: Umbrella Sampling. *J. Comput. Phys.* **1977,** *23* (2), 187-199.
30. Kumar, S.; Rosenberg, J. M.; Bouzida, D.; Swendsen, R. H.; Kollman, P. A. The Weighted Histogram Analysis Method for Free-Energy Calculations on Biomolecules. I. The Method. *J. Comput. Chem.* **1992,** *13* (8), 1011-1021.
